# MCMSeq: Bayesian hierarchical modeling of clustered and repeated measures RNA sequencing experiments

**DOI:** 10.1186/s12859-020-03715-y

**Published:** 2020-08-28

**Authors:** Brian E. Vestal, Camille M. Moore, Elizabeth Wynn, Laura Saba, Tasha Fingerlin, Katerina Kechris

**Affiliations:** 1 Center for Genes, Environment and Health, National Jewish Health, 1400 Jackson St, Denver, 80206 CO USA; 2 Department of Biostatistics and Informatics, University of Colorado, Anschutz Medical Campus, Aurora, CO USA; 3 Department of Pharmaceutical Sciences, University of Colorado Skaggs School of Pharmacy and Pharmaceutical Sciences, Aurora, CO USA

**Keywords:** RNA-Seq, Markov chain Monte Carlo, Longitudinal data, Correlated data

## Abstract

**Background:**

As the barriers to incorporating RNA sequencing (RNA-Seq) into biomedical studies continue to decrease, the complexity and size of RNA-Seq experiments are rapidly growing. Paired, longitudinal, and other correlated designs are becoming commonplace, and these studies offer immense potential for understanding how transcriptional changes within an individual over time differ depending on treatment or environmental conditions. While several methods have been proposed for dealing with repeated measures within RNA-Seq analyses, they are either restricted to handling only paired measurements, can only test for differences between two groups, and/or have issues with maintaining nominal false positive and false discovery rates. In this work, we propose a Bayesian hierarchical negative binomial generalized linear mixed model framework that can flexibly model RNA-Seq counts from studies with arbitrarily many repeated observations, can include covariates, and also maintains nominal false positive and false discovery rates in its posterior inference.

**Results:**

In simulation studies, we showed that our proposed method (MCMSeq) best combines high statistical power (i.e. sensitivity or recall) with maintenance of nominal false positive and false discovery rates compared the other available strategies, especially at the smaller sample sizes investigated. This behavior was then replicated in an application to real RNA-Seq data where MCMSeq was able to find previously reported genes associated with tuberculosis infection in a cohort with longitudinal measurements.

**Conclusions:**

Failing to account for repeated measurements when analyzing RNA-Seq experiments can result in significantly inflated false positive and false discovery rates. Of the methods we investigated, whether they model RNA-Seq counts directly or worked on transformed values, the Bayesian hierarchical model implemented in the mcmseq R package (available at https://github.com/stop-pre16/mcmseq) best combined sensitivity and nominal error rate control.

## Background

As the cost of sequencing decreases, the complexity and size of RNA sequencing (RNA-Seq) experiments are rapidly growing. In particular, paired, longitudinal and other correlated study designs are becoming commonplace [1, 2]. Longitudinal studies of how gene expression changes over the disease course and under differing treatment and environmental conditions are critical to understanding how disease evolves in individual patients and for developing accurate biomarkers of disease progression and treatment response. However, the most popular RNA-Seq data analysis tools are not equipped to analyze correlated and longitudinal data [3].

Failing to account for correlation between repeated measurements can result in misleading inferences [4]. For example, when estimating changes in gene expression over time, if there is strong within-subject correlation, estimated standard errors will be too large if correlation is ignored, reducing power to detect differential expression. As well-developed statistical methods and software tools are lacking, some have proposed analyzing correlated RNA-Seq data with DESeq2 or edgeR by including all levels of the clustering variable as fixed effects in the regression model [3, 5]. In this case, the number of parameters necessary to account for correlation is equal to the number of subjects or clusters. This may result in a model that is too complex for the number of observations in the study, leading to overfitting. When overfitting is present, some of the findings of the analysis come from fitting noise, resulting in spurious associations that cannot be reproduced in future studies [6]. Cui *et al* (2016) also noted that this strategy led to inflated false positive rates [3]. In addition, if a regression parameter for each subject is included in a model, other effects of interest, such as group differences at individual time points, may not be estimable. Consequently, a method that can analyze RNA-Seq data in a flexible manner while including covariates and accounting for repeated measures is needed.

As RNA-Seq data are over-dispersed counts, traditional linear mixed models, which assume a normal distribution, are inappropriate. In non-correlated settings, RNA-Seq data are typically modeled using a negative binomial (NB) distribution in a generalized linear model framework [7*,*^8^]. Since estimation of the dispersion parameter can be unstable or biased, particularly for data with low mean counts and small sample sizes [9*–*13], specialized RNA-Seq analysis packages, such as DESeq2 and edgeR, shrink estimates of dispersion parameters for individual genes towards a common estimate [14*–*16].

The NB generalized linear mixed model (NBGLMM) is an extension of NB regression that allows for the inclusion of random effects to account for correlation between observations [4*,*^17^], and would seem a natural choice for the analysis of correlated RNA-Seq data. However, NBGLMMs do not have closed form solutions, and thus numerical optimization algorithms must be used to maximize the likelihood to obtain parameter estimates [17]. With small to modest sample sizes, achieving a reliable level of convergence can be even more problematic than in traditional NB regression. There are also concerns about inflated false positive rates for both NB generalized linear models (NBGLM) and NBGLMMs, since standard errors for regression coefficients are estimated assuming that the NB dispersion parameter is known rather than estimated from the data [18].

There have been several recent developments in the analysis of correlated RNA-Seq data. For studies of related individuals, MACAU (Mixed model Association for Count data via data AUgmentation) [19] uses a Poisson mixed model to account for relatedness and population structure within an RNA-Seq experiment by allowing the user to supply a known kinship matrix. Since the correlation structure is specified a priori, this method is not designed to analyze data from longitudinal studies where the expected correlation among samples is unknown. PLNSeq [20] conducts differential expression analysis on RNA-Seq data generated from correlated samples, assuming the marginal distribution of the read counts is the compounding of the Poisson and the lognormal distributions; however, the method cannot adjust for covariates and can only be used to analyze data from single group study designs. The authors have also published a similar model using a Poisson Gamma formulation of the NB [21].

PairedFB is a Bayesian model that can test for differential expression between two groups when there are paired observations [22]. However, this model is limited to paired observations and does not allow for additional covariates. ShrinkBayes is another Bayesian approach which uses a NB hierarchical model to account for clustering and to share information across genes when estimating dispersion parameters [23]. Spike and slab shrinkage priors are used in an attempt to control false discovery rates (FDRs). The main drawbacks of this approach are that it is computationally intensive, it can be difficult for those unfamiliar with the method to develop reasonable priors (e.g. choose the form of prior for NB dispersion parameter) or make other modeling choices, and others have noted inflated error rates in some applications [22*,*^24^]. The VarSeq method from the tcgsaseq R package uses a variance component score test to identify gene sets whose expression varies over time and can account for repeated measures [25]. While it is possible to apply these tests to individual genes, the method was developed and validated specifically for gene sets and testing characteristics for performing gene-wise tests is unknown. Lastly, tools from the limma R package, which was originally developed for the analysis of microarray experiments but can be applied to RNA-Seq data via the VOOM transformation, have also been proposed for situations with repeated measures [26*–*28]. In this process, the average correlation between repeated or clustered observations across all genes is computed, and this value is then accounted for during the linear model fits in a standard limma analysis. While mixed models are fit to estimate the duplicate-correlation measure, final inference is performed using standard limma regression models.

In this work, we propose to model correlated and longitudinal RNA-Seq data using a simple Bayesian NB hierarchical model. This model allows for the inclusion of random effects that can be used to account for correlation between samples, which may occur in studies where subjects are followed over time, in studies of related individuals, such as paired siblings, or when multiple samples are taken from the same subject (for example, RNA-Seq on paired blood and biopsy samples). We use non-informative priors with large variances, making prior selection straightforward. If desired, information can be borrowed across genes when developing the prior for the dispersion parameter in a way that mimics the information sharing in edgeR and DESeq2. Unlike frequentist methods of estimation, estimates of variability for parameters and statistical tests account for the fact that dispersion parameters are estimated, resulting in better controlled false positive rates than frequentist NBGLMMs. We calculate a contour probability from the posterior distribution, a corollary to a two-sided *p*-value, which can undergo common FDR corrections. These methods are implemented in the mcmseq R package, which fits models with a Markov-chain Monte Carlo (MCMC) algorithm that has been optimized in C++ and can be run in parallel to speed computation time. We illustrate our methods in the analysis of publicly available longitudinal whole-blood RNA-Seq data from subjects with latent tuberculosis (TB) infection.

## Methods

### MCMSeq: a Bayesian NBGLMM for RNA-Seq data

**Fig. 1:**
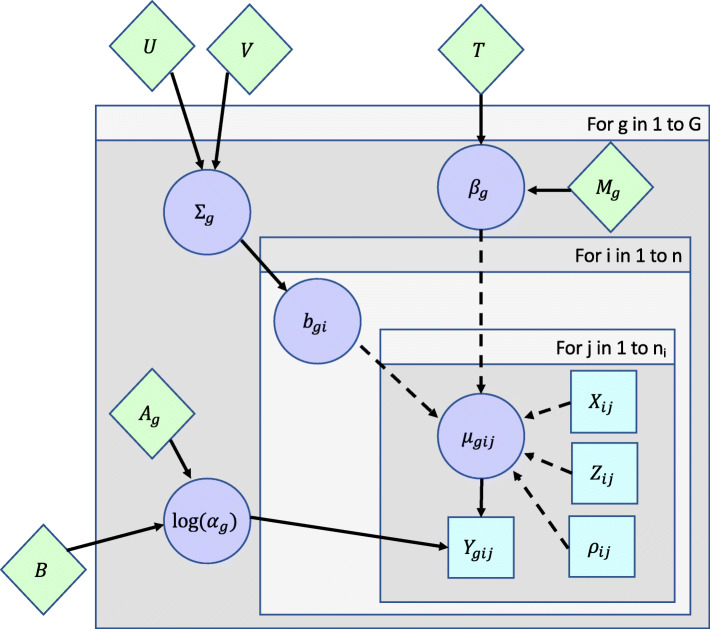
Directed acyclic graph of the MCMSeq Model. Squares represent observed data, while circles represent model parameters to be estimated. Diamonds represent prior parameters and hyper-parameters. Solid arrows indicate stochastic dependence, while dashed arrows indicate deterministic dependence

Similar to other commonly used RNA-Seq analysis tools, we model the observed data with a NB distribution using a log link function. To account for correlation, we allow random effects to be included in the model in a NBGLMM framework. Let *Y*_*gij*_ be the expression of gene *g* from subject *i* at time point or observation *j*, then

1 $$\begin{array}{@{}rcl@{}}  Y_{gij} &\sim& \mathcal{NB}\left(\mu_{gij}, \alpha_{g}\right) \\ \log\left(\mu_{gij}\right) &=& X_{ij}\boldsymbol{\beta_{g}} + Z_{ij}\boldsymbol{b_{gi}} + \rho_{ij}\\ \boldsymbol{b_{gi}} &\sim& \mathcal{N}\left(0, \Sigma_{g}\right)  \end{array} $$

where *μ*_*gij*_ is the mean expression of gene *g*∈{1,⋯,*G*} in subject *i*∈{1,⋯,*I*} at observation *j*∈{1,⋯,*n*_*i*_} and *α*_*g*_ is the dispersion parameter for gene *g*, which is related to the variance of *Y*_*gij*_ by Var (*Y*_*gij*_) = $\mu _{gij} + \alpha _{g} \mu _{gij}^{2}$. ***β***_***g***_ is a *p* x 1 vector of fixed effect regression coefficients for gene *g*, *X*_*ij*_ is a 1 x *p* row vector of fixed effects covariates for subject *i* at observation *j*, ***b***_***gi***_ is a *q* x 1 vector of random effects for gene *g* and subject *i*, *Z*_*ij*_ is a 1 x *q* row vector of random effect covariates for subject *i* at observation *j*, and *ρ*_*ij*_ is an offset for subject *i* at observation *j*, which can be used to account for differences in sequencing depth between samples. A graphical representation of the hierarchical model is presented in Fig. 1. We assume that for each gene, the subject-specific random effects (e.g. random intercepts, random slopes or time effects for individuals or clusters) are normally distributed with mean 0 and variance *Σ*_*g*_. In addition to accounting for repeated measures made on subjects in longitudinal studies, random effects can be used to account for correlation in other study designs, such as studies of related family members or paired siblings or studies in which RNA-Seq is performed on multiple types of samples (e.g. blood and biopsied tissue) from the same subject. This flexible framework also allows for the inclusion of both categorical and continuous covariates, where the latter can be modeled in a number of different ways (e.g. linear relationships, higher order polynomial terms, or even regression splines).

### Prior specification

We fit the NBGLMM as a Bayesian hierarchical model using MCMC. The Bayesian NBGLMM is completed by specifying priors (and hyper-priors) for ***β***_***g***_, *Σ*_*g*_, and *α*_*g*_:

$$\begin{array}{@{}rcl@{}} \boldsymbol{\beta_{g}} &\sim& \mathcal{MVN}\left(M_{g}, T\right)  \\ \Sigma_{g} &\sim& \mathcal{IW}(U, V) \\ \log\left(\alpha_{g}\right) &\sim& \mathcal{N}\left(A_{g}, B\right)  \end{array} $$

where *M*_*g*_ is a vector of length *p* containing the prior means for the regression coefficients for gene *g*, *T* is a *p* x *p* covariance matrix, $\mathcal {MVN}$ is the multivariate normal distribution, and $\mathcal {IW}$ is the inverse Wishart distribution with *q* x *q* scale matrix *U* and *V*>*q*−1 degrees of freedom. *A*_*g*_ is the prior mean of the log normal distribution for the dispersion parameter for gene *g* and *B* is the prior variance, which is common across genes.

In practice, we set the first element of *M*_*g*_ (the prior mean for the intercept) to be the log of the mean number of counts across samples for that gene; all other elements of *M*_*g*_ are set to 0. This prior is uninformative, as it assumes that observations come from a common distribution with a single mean and that there are no differences between groups or time points. For *T*, we use a diagonal matrix with *s*^2^ on the diagonal, and choose *s*^2^, the prior variance, to be large. We have used a prior variance of 7^2^=49 for all analyses in this paper. The influence of these hyperparameters on inference is well known. Choosing non-zero means for regression coefficients is uncommon and we do not recommend it; this could result in increases in type 1 errors for truly null genes and could also increase type 2 errors for differentially expressed genes if the differential expression is in the opposite direction of the assumed prior mean. For the prior variance *T*, choosing smaller values increases the influence of the prior on inference; assuming the priors for the regression coefficients are centered at zero, this could lead to a loss of power. The influence of prior hyper-parameters on inference diminishes with increasing sample size.

We specify *U* as a *q* x *q* identity matrix and *V*=*q*+1. For models including a single random effect for each subject (i.e. *q*=1 as in our simulation study using a random intercept for each subject), we assume *Σ*_*g*_ has an inverse gamma prior with parameters *U* and *V*. The prior becomes more diffuse and uninformative as *U* and *V* decrease; therefore, we set these parameters to small values (e.g. 0.01) in order to be uninformative. We investigated the influence of choice of *U* and *V* in our simulation study in Supplementary materials Section 2.3.5 and found values of 0.1, 0.01, 0.001 all maintained false discovery rates at or below their nominal levels; however, the most informative prior (value of 0.1) resulted in a loss of power for between subject tests and slightly inflated type 1 error rates for within subject and interaction tests. Therefore, we recommend values of 0.01 and smaller.

To stabilize estimation of the dispersion parameter at small sample sizes, we share information across genes when specifying the log normal prior for the dispersion parameter in a similar spirit to the dispersion estimation in the DESeq2 pipeline [15]. First, for each gene, we calculate the average log(CPM) across samples and a naïve method-of-moments estimate of the dispersion parameter. The naïve estimate is calculated by first scaling the data to the median library size and then calculating the mean, *m*_*g*_, and variance, *v*_*g*_ of the scaled counts for each gene. The naïve dispersion estimate for each gene is given by $\frac {v_{g} - m_{g}}{m_{g}^{2}}$. Locally weighted polynomial regression (LOESS) is then used to model the relationship between the log of the method-of-moments dispersion estimates and the average log(CPM). *A*_*g*_ is then set to the expected log(dispersion) at the average log(CPM) for gene *g* based on the LOESS regression. *B* is set to (*k*·*τ*)^2^ where *k* is an inflation factor and *τ* is the residual standard deviation from the LOESS fit. The method-of-moments dispersion estimates are over-estimates since they do not take into account fixed or random effects that explain variability in the data. Therefore, we typically choose *k*>1 in order to include a larger range of potential dispersion values in our prior. In practice, we have found a *k* of 2 tends to work well and use *k*=2 for all analyses presented in this paper (See Supplementary material Section 2.3.6 for simulation studies showing the influence of *k* on testing characteristics). Type 1 error rates and FDR increase with k, such that values of *k*<2 tend to be more conservative and values of *k*>2 are more liberal. As sample size increases, the dispersion prior has less influence and all values of *k* perform similarly.

### Posterior estimation and convergence criteria

Posterior estimation is performed with a hybrid Metropolis-Hastings/Gibbs MCMC sampler (see Supplementary materials Section 1.1 for detailed steps). A weighted least squares (WLS) proposal, as introduced by Gamerman [29], is used to jointly update ***β***_***g***_ and ***b***_***g***_; *α*_*g*_ is updated using a random walk proposal and *Σ*_*g*_ can be updated using an appropriate Gibbs step. In this framework, the posterior distribution of regression coefficients naturally incorporates the uncertainty associated with simultaneous estimation of the dispersion parameter. Access to the full posterior distribution also makes inference about quantities that involve complex combinations of model parameters (e.g. the heritability calculation from Rudra et al.) straightforward [30].

Convergence of the chain for each gene is examined in two ways: acceptance rate and Geweke statistics comparing the first 20% of the chain (after discarding burn-in) to the final 50% of the chain [31]. For each gene, acceptance probabilities are computed for the vector of regression coefficients ***β***_***g***_ and the dispersion parameter *α*_*g*_; *Σ*_*g*_ is always accepted and thus ignored for this diagnostic. Additionally, *p*-values for the Geweke statistics (i.e. t-tests) are calculated for all model parameters, and are then adjusted for multiple comparisons across genes using the Benjamini-Hochberg method [32]. We deemed any gene that had an acceptance rate <10% or an adjusted Geweke p-vlaue <0.05 for any model parameter to be a convergence failure. See Supplementary materials Section 1.3 for more details.

### Posterior inference and adjustments for multiple comparisons

To perform posterior inference on individual regression parameters (or linear combinations of regression parameters), there are multiple potential approaches. One option is to use Bayes factors to compare the likelihood of a given parameter value in the prior versus the posterior distribution. A major drawback of this approach is that adjusting for multiple comparisons when using Bayes factors is not well characterized. Alternatively, we use the contour probability originally proposed by Box and Tiao [33], which can be thought of as the Bayesian corollary to a two-sided *p*-value for the null hypothesis that a parameter *θ* equals a given value *θ*_0_ (See Supplementary Fig. 1). Formally, the contour probability *p*_*c*_ is defined as

2 $$ p_{c} = 1 - P\left(p(\theta|Y) > p(\theta_{0}|Y)\right)  $$

Since the NBGLMM does not have a closed-form posterior distribution, Monte Carlo estimation is required. One strategy for computing *p*_*c*_ in this scenario is to construct a smoothed approximation of the posterior distribution using kernel density estimation with the MCMC sample {*θ*^(1)^,⋯,*θ*^*k*^}, and to then estimate *p*_*c*_ as the proportion of samples for which *p*(*θ*_0_|*Y*)>*p*(*θ*^(*k*)^|*Y*). However, the results are dependent on the choice of kernel function and smoothing bandwidth [34*,*^35^]. To avoid this problem, we instead take a simplified approach by estimating the contour probability as

3 $$ p_{c} = 2 \cdot \frac{1}{k} \sum\limits_{j=1}^{k} I_{\left(\theta^{(j)} \cdot \hat{\theta}\right) < 0}  $$

where *I* is an indicator function and $\hat {\theta }$ is the posterior median of *θ*. That is, we estimate the contour probability as twice the proportion of MCMC samples that lie on the opposite side of 0 as the posterior median, which is twice the posterior probability of the null in a one-sided hypothesis testing framework (See Supplementary Fig. 2).

The final step is to account for multiple comparisons. A common Bayesian FDR control approach is to construct a set of features (i.e. genes) for which the average posterior probability of being differentially expressed is above some threshold (or equivalently, that the average probability of being equally expressed is below some threshold) [36]. In this context with contour probabilities and a target FDR level *α*, the procedure would entail sorting the gene-specific $p_{c}^{(g)}$’s for a given parameter in ascending order, and then finding the cutoff point $l^{*} = \max \left \{l: \frac {1}{l} {\sum \nolimits }_{j=1}^{l} p_{c}^{(j)} < \alpha \right \}$. We investigated using this strategy, but found it resulted in substantial FDR inflation (see Supplementary Table 2), and thus quickly abandoned it. Instead we apply a Benjamini-Hochberg procedure for controlling FDRs to the contour probabilities. While the Benjamini-Hochberg method was developed for application to *p*-values, the relationship between one-sided posterior probabilities and one-sided *p*-values has been discussed by Casella and Berger [37]. Moreover, in our simulations, contour probabilities behaved very much like traditional *p*-values for features that showed no true differences; their distributions were nearly uniform (Supplementary Fig. 3). We found this method controlled FDRs very well in our simulation studies and has been used to generate all results presented in this paper. For more details see Supplementary materials Section 1.2.

### Implementation

The MCMSeq method is implemented in the mcmseq R package, which contains a detailed vignette that illustrates these methods on a simulated data set. The package is written in C++ via the Rcpp, RcppArmadillo, and RcppParallel R packages, the last of which allows the chains for each gene to be automatically run in parallel to shorten computation time [38*–*40]. In the fitting function, users can specify all hyper-parameters in the priors, though non-informative values that generally worked well in the simulation studies and real data applications are provided as defaults.

While MCMC samplers for Bayesian hierarchical negative binomial models can be developed and fit with other packages or software, such as STAN [41], OpenBugs [42], or SAS software’s MCMC procedure, these are general Bayesian model fitting tools that have not been optimized for high-throughput analysis of RNA-Seq data. As such, the user would be required to write additional code to specify the model, fit the model to each gene, and to summarize results over all genes. In addition, since these are general tools, samplers may not be the most efficient for fitting NBGLMMs. For example, we explored fitting our proposed model to a single feature in the N=3 simulated datasets using the rstan package [43]. This took approximately 2.5 minutes; for a typical RNA-Seq dataset of 15,000 genes, this would result in 620 hours of computation time, compared to approximately 30 minutes for the same dataset using mcmseq. The mcmseq package makes fitting Bayesian NBGLMMs more accessible by automating the process of model fitting and summarizing results over tens of thousands of genes typically found in an RNA-Seq analysis.

### Simulation study

#### Data generation

To evaluate the testing characteristics of MCMSeq and to compare to other potential analysis strategies, we performed a simulation study. Datasets were simulated using a two group design (e.g. treatment and control) with paired observations (e.g. baseline and follow-up) on each subject. Count data were simulated from a NB distribution as follows:

4 $$\begin{array}{@{}rcl@{}}  Y_{gij} &\sim& \mathcal{NB}\left(\mu_{gij}, \alpha_{g}\right)  \\ \log\left(\mu_{gij}\right) &=& \beta_{g0} + \beta_{g1} I_{T_{i}} + \beta_{g2} I_{F_{ij}} + \beta_{g3}I_{T_{i}} I_{F_{ij}} + b_{gi}  \\ b_{gi} &\sim& \mathcal{N}\left(0, \sigma_{gb}^{2}\right) \end{array} $$

where $I_{T_{i}}$ is an indicator function that equals 1 if subject *i* is in the treatment group, $I_{F_{ij}}$ is a similar indicator for if observation *j* is the follow-up, and *b*_*gi*_ is the random intercept for gene *g* and subject *i*. Simulated data sets were generated with a combination of null and differentially expressed (mixed effect sizes) features, as summarized in Table 1. In order to mimic real data as much as possible, values for *β*_*g*0_, *α*_*g*_, and $\sigma _{gb}^{2}$ were drawn from an empirical distribution of mean counts per million (CPM), dispersion (*α*_*g*_), and random intercept variance $\left (\sigma _{gb}^{2}\right)$ observed across human samples in several real RNA-Seq data sets that had repeated measures [1*,*^2^] (Supplementary materials Section 2.1).

**Table 1:** Summary of simulated datasets

**Number of datasets**	10
**Number of features per dataset**	15,000
**Sample sizes**	3, 5, and 10 per group
	(6, 10 and 20 total)
**Model parameters**	
*β*_*g*1_: Difference in log(expression) between	0 (all features)
treatment and control at baseline	
*β*_*g*2_: Change in log(expression) over time	0 (all features)
in the control group	
*β*_*g*3_: Difference in change in log(expression)	0 (80% of features)
over time between the treatment and	ES^1^ (10% of features), -ES (10% of features)
control groups	
*β*_*g*0_, *α*_*g*_, $\sigma _{gb}^{2}$	Drawn from an empirical distribution based on human
	samples in real RNA-Seq data sets with repeated measures
**Additional comparisons**	
*β*_*g*1_+*β*_*g*3_: difference in log(expression)	0 (80% of features)
between control and treatment at	ES (10% of features), -ES (10% of features)
follow-up	
*β*_*g*2_+*β*_*g*3_: Change in log(expression)	0 (80% of features)
over time in the treatment group	ES (10% of features), -ES (10% of features)

^1^ES is drawn from a Gamma distribution with mode of log(2) and a standard deviation of 0.5

#### Methods compared and implementation

We analyzed each simulated data set using the methods described in Table 2 (see also Supplementary materials Section 2.2). Genes were filtered from the analysis if >75% of the samples had less than 1 CPM (i.e. at least *m* samples must have a CPM >1 where *m* is the number of samples in the smallest experimental unit of interest). We also tested the sensitivity of our simulation results to additional filtering schemes and found similar testing characteristics and relative performance of the methods. See Supplementary materials Section 2.3.7 for full details. To account for differences in library size between samples, we used the default median of ratios method for DESeq2 and the trimmed mean of M values for edgeR. For MCMSeq, CPGLMM, NBGLMM and ShrinkBayes, we took the natural log of the DESeq2 median of ratios values as an offset. VarSeq, limma, and LMM use transformed counts and do not require an offset. For edgeR and DESeq2, models were fit ignoring correlation (dropping the random effect terms from Eq. 4) and accounting for correlation by replacing the random effects *b*_*gi*_ from Eq. 4 with fixed effects (edgeR* and DESeq2*). This requires removing both the intercept (*β*_*g*0_) and the main effect of treatment (*β*_*g*1_) from the model, as they are not estimable. For all other methods, both fixed and random effects were included in the model as specified in Eq. 4. A summary of the practical and theoretical advantages and disadvantages for each of the methods and their software implementations can be found in Supplementary Table 1.

**Table 2:** Summary of methods compared

**Method**	**R-Package**	**Description**	**Reference**
MCMSeq	mcmseq	Bayesian negative binomial hierarchical model, assuming the following	
		hyper-parameters: *T*=7^2^*I*_4_, where *I*_4_ is a 4 x 4 identity matrix, *U*=	
		*V*=0.01, *A*_*g*_= centered on empirical prior as described in 2.3, $B=4\cdot \sigma ^{2}_{re}$	
		Chains were run for 30,000 iterations and 10% was discarded as burn-in.	
CPGLMM ^*‡*^	cplm	Compound Poisson generalized linear mixed model fit using maximum	[44]
		likelihood.	
DESeq2*	DESeq2	DESeq2 analysis using default settings. To account for correlation,	[15]
		subjects are included as fixed effects in the model by replacing the	
		random effects in Equation 4 with fixed effects.	
DESeq2	DESeq2	DESeq2 analysis using default settings, including only fixed effects in	[15]
		Equation 4. Correlation is ignored.	
edgeR*	edgeR	edgeR analysis using default settings. To account for correlation,	[14]
		subjects are included as fixed effects in the model by replacing the	
		random effects in Equation 4 with fixed effects.	
edgeR	edgeR	edgeR analysis using default settings, including only fixed effects in	[14]
		Equation 4. Correlation is ignored.	
limma	limma	Count data were transformed for analysis with limma using voom.	[26]
		The duplicateCorrelation function using subject id as the blocking	[27]
		factor was used to account for correlation.	
LMM	lmerTest	Data were first transformed using DESeq2’s variance stabilizing	[45]
		transformation. Linear mixed models, which assume a normally	
		distributed errors, were then fit to the transformed counts.	
MACAU	MACAU2	Poisson GLMM with random effects to account for over-dispersion and	[19]
		correlation among observations. A known covariance matrix must be supplied;	
		we used the covariance of the centered and scaled gene expression matrix as	
		shown in the third example of [19].	
NBGLMM	glmmADMB	Frequentist negative binomial generalized linear mixed model fit using	[46]
		maximum likelihood.	
ShrinkBayes	ShrinkBayes	Bayesian hierarchical model using shrinkage priors to control false	[23]
		discovery rates. We used a negative binomial distribution for the count	
		data and shrinkage priors for all model parameters involved in statistical	
		tests. All other parameters were set to default values.	
VarSeq ^*‡*^	tcgsaseq	Model free method for identifying gene sets whose expression changes	[25]
		over time. Counts are transformed to log(CPM) for analysis. The	
		asymptotic test was used to generate *p*-values.	

^*‡*^Results presented in Supplementary materials, Section 2.3

We compare the performance of these analysis strategies in terms of type 1 error rate, FDR, and power (recall) for three statistical tests. First, *β*_*g*1_+*β*_*g*3_=0 tests for differences in expression between the treatment and the control group at follow-up. We refer to this as a “between-subject” test, as inference involves only independent observations. Second, *β*_*g*2_+*β*_*g*3_=0 tests for a change in expression over time in the treatment group. We refer to this as a “within-subject” test as inference involves the paired observations. Lastly *β*_*g*3_=0 tests if there is a difference in the change in expression over time between the treatment and control groups. We refer to this as an “interaction” test, which depends on both between- and within-subject information. For each simulation scenario, we calculate type 1 error rate, FDR, and power for each contrast within each dataset and then average across the 10 datasets. We evaluate these characteristics over a range of significance thresholds (Table 3). To evaluate power and FDR, we first applied a Benjamini-Hochberg correction to the *p*-values for each contrast within each dataset[32]. However, this correction was not applied to results from ShrinkBayes, as ShrinkBayes reports Bayesian FDR values rather than *p*-values.

**Table 3:** Testing characteristics compared

Characteristic	Description	Significance Thresholds	P-Value
Type 1 Error Rate	$\frac {\text {N false positives}} {\text {N null genes}}$	0.0001, 0.01, 0.05, 0.1	Unadjusted
(False Positive Rate)		for unadjusted *p*-values	
False Discovery Rate	$\frac {\text {N false positives}} {\text {N significant genes}}$	0.01, 0.05, 0.1	FDR Adjusted
Power (Recall)	$\frac {\text {N true positives}} {\text {N Differntially Expressed Genes}}$	0.01, 0.05, 0.1	FDR Adjusted

### Real data analysis

We apply the analysis strategies described above to a publicly available longitudinal RNA-Seq dataset of 112 whole blood samples from 40 subjects in recent contact with patients with active TB infection (GEO Datasets: GSE107994, GSE107993). The cohort was recruited between September 2015 and September 2016 at the Glenfield Hospital, University Hospitals of Leicester NHS Trust, Leicester, UK. The study design and methods have been described in detail elsewhere [1]. Subjects were followed for a median of 86 days and had a median of 3 observations per subject.

Over the course of the study, 9 subjects developed active TB infection and were classified as TB progressors (TBP). Of the remaining 31 subjects, 16 tested positive on the M. tuberculosis antigen-specific interferon-gamma (IFN- *γ*) release assay (IGRA) and were classified as having latent TB infection (LTBI). The remaining 15 subjects tested negative on the IGRA and were classified as controls. Previous analyses of the data evaluated a risk score based on a panel of 20 genes designed to distinguish LTBI from TBP and found that among subjects that remained healthy over the course of the study, risk scores were stable over time, while for most subjects that developed active TB, risk scores increased over time. In addition, it was noted that at baseline (first study observation after recruitment) the risk score was, on average, slightly elevated in the LTBI and TBP groups compared to the control group [1].

#### Data pre-processing and implementation

Count tables and metadata were downloaded from the GEO DataSets website. The data included 17,793 genes with at least 1 non-zero count. Before analysis, we filtered out lowly expressed genes using the same general strategy as for the simulated data. Here, this meant all genes that did not have greater than 1 CPM in at least 9 of the 112 samples (9 was the sample size in the smallest experimental group of interest) were dropped, which reduced the total number of genes analyzed to 15,993.

The goal of our analysis is to compare longitudinal changes in gene expression between subjects that develop active TB infection and others. As the number and timing of observations differed greatly between subjects, we chose to include time as a continuous predictor in our models. Since there were relatively few observations per subject, with 80% of all subjects having 3 or fewer observations and with 20% of controls, 56% of progressors and 44% of LTBI having 2 or fewer observations, we decided to model time using a linear effect. For studies with larger numbers of repeated measures, regression splines or polynomials could be used to model potentially non-linear changes over time. We included group by time interactions in the model to allow the 3 groups to have different changes in expression over time, in addition to different expression at baseline. We used the following basic regression model:

5 $$\begin{array}{@{}rcl@{}}  \log\left(\mu_{gij}\right) &=& \beta_{g0} + \beta_{g1} I_{L_{i}} + \beta_{g2} I_{P_{i}} + \\ && \beta_{g3}t_{ij} + \beta_{g4}t_{ij} I_{L_{i}} + \beta_{g5}t_{ij} I_{P_{i}} + b_{gi} +\rho_{ij}  \end{array} $$

where *t*_*ij*_ is the number of days since baseline for subject *i* at observation *j*, $I_{L_{i}}$ is an indicator function that equals 1 if subject *i* is in the LTBI group, $I_{P_{i}}$ is a similar indicator for if subject *i* is in the TBP group, *b*_*gi*_ is the random intercept for gene *g* and subject *i*, and *ρ*_*ij*_ is an offset. In this model, $e^{\beta _{g0}}\phantom {\dot {i}\!}$ represents the mean expression of gene *g* in the control group at baseline, *β*_*g*1_ is the log(fold change) comparing the LTBI group to the control group at baseline, and *β*_*g*2_ is the log(fold change) comparing the TBP group to the control group at baseline. *β*_*g*3_ is the change in log expression per day for the control group, *β*_*g*4_ is the difference in the change in log(expression) per day between the LTBI and controls groups, and *β*_*g*5_ is the difference in the change in log(expression) per day between the TBP and controls groups. Additional contrasts of interest include *β*_*g*3_+*β*_*g*4_ (change per day in the LTBI group), *β*_*g*3_+*β*_*g*5_ (change per day in the TBP group), *β*_*g*2_−*β*_*g*1_ (difference between TBP and LTBI groups at baseline), and *β*_*g*5_−*β*_*g*4_ (the difference in change per day between TBP and LTBI groups). All models were fit as described in the “Simulation study” section. The Benjamini Hochberg method was used to adjust *p*-values for all methods except ShrinkBayes, which reports a Bayesian FDR [32]. Numbers of models that failed to converge and number of significant genes for regression coefficients and contrasts of interest were compared between the analysis strategies.

#### Functional enrichment analyses

To better understand the genes that significantly changed over time in the TBP group, we performed functional enrichment analysis on the differentially expressed genes (DEGs) identified with the MCMSeq method using Panther 14.0 with the GO Biologic Process Complete annotation database. We used a 0.05 FDR level to identify GO terms that were significantly enriched. We further filtered results to include only GO terms with at least 50 genes, ≥ 10% overlap of the GO terms with the DEG list, and a raw *p*-value of less than 0.001.

In addition, cross-sectional microarray [47*,*^48^] and RNA-Seq studies of whole blood gene expression [49], have identified sets of genes with differential expression between LTBI and active TB groups. To understand if these genes also show changes over time in subjects that develop active TB, we performed an enrichment analysis. Fisher’s exact test was used to determine if the cross-sectional gene sets were over-represented in the MCMSeq DEG list for changes in expression over time in the TBP group. To calculate the expected number of overlapping genes, we multiplied the number of DEG’s by the size of the gene set and divided by the total number of genes analyzed (15,993).

## Results

### Simulation results

#### Convergence

Across all sample sizes, NBGLMM and LMM methods had substantial proportions of models that failed to converge (Table 4), ranging from ≈4*%* to ≈20*%* of all genes tested at a given sample size. DESeq2 ignoring correlation had minor convergence issues at smaller sample sizes. MCMSeq had minor issues at the largest sample size, but the number of genes affected was <1*%*. Features that failed to converge in both MCMSeq and DESeq2 ignoring correlation shared similar characteristics: small counts, large dispersion, large random intercept variance, repeated zero values, and fewer unique count values. The NBGLMM had the opposite pattern, with the genes that failed to converge tending to have larger counts, smaller dispersion, and smaller random intercept variance. For the LMM, there were no obvious distinguishing features of the genes that failed to converge (Supplementary Table 3).

**Table 4:** Number of models that failed to converge out of the 150,000 simulated genes in the null + mixed effect size simulation

Method	N = 3	N = 5	N = 10
MCMSeq	184	335	1,526
DESeq2*	0	0	0
DESeq2	1,295	979	0
edgeR*	0	0	0
edgeR	0	0	0
limma	0	0	0
LMM	32,060	23,300	14,150
MACAU	39	5	2
NBGLMM	6,113	9,992	11,995
ShrinkBayes	0	0	0

#### Type 1 error rates

**Fig. 2:**
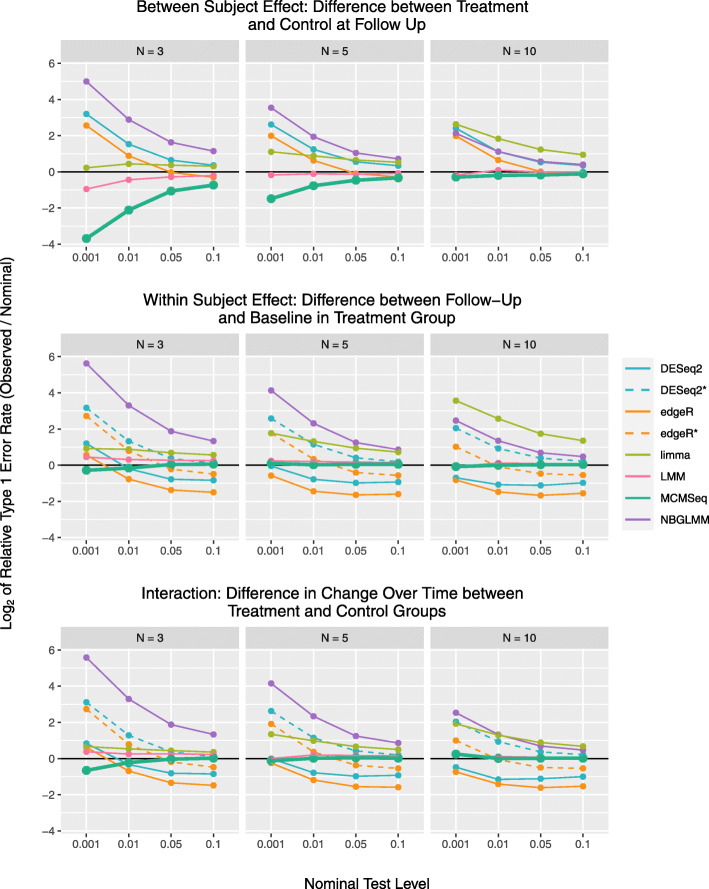
Relative type 1 error rates (false positive rates) for the 3 tests of interest. Relative rates are plotted on the log2 scale, for the 4 different testing thresholds at each of the 3 sample sizes examined. Values greater than 0 indicate type 1 error rates higher than nominal levels (e.g. a value of 1 means the observed rate was double what one would expect), while values less than 0 indicate conservative behavior (e.g. -1 means observed rate was half of what is expected). Values in the figure are based on the average rate calculated across the 10 data sets simulated at each sample size. Supplementary Fig. 5 shows the variability in observed rates for each of the methods. Results are consistent across the data sets, indicating the mean of the results is an accurate reflection of each method’s performance

In Fig. 2, we compare type 1 error rates between analysis strategies using just the null genes (also see Supplementary Fig. 6 and Supplementary Table 4). ShrinkBayes was not evaluated in terms of type 1 error, since it only reports Bayesian FDR’s. Estimated error inflation is based on averages across the 10 simulated data sets, with each data set tending to produce consistent results (see Supplementary Fig. 5). For “between-subject effects,” such as differences between groups at a single time point, MCMSeq and the LMM on transformed counts have observed rates at or slightly below the nominal levels. Conversely, the NBGLMM and DESeq2 (ignoring correlation) have inflated type 1 error rates. For these methods, type 1 error rate inflation worsens with smaller significance thresholds, though this behavior is attenuated with increasing sample size. edgeR ignoring correlation maintains 0.05 and 0.1 significance levels, but has inflated type 1 error rates for 0.001 and 0.01 levels. MACAU had significant inflation at *n*=3 and *n*=5, but was overly conservative at *n*=10. At *n*=3, limma performed reasonably well, but as the sample size increased, the type 1 error inflation worsened to the point of being one of the most inflated methods at *n*=10.

For “within-subject effects,” such as changes over time within a single group, NBGLMM and DESeq2 including subject as a fixed effect have severely inflated type 1 error rates. Conversely, edgeR and DESeq2 ignoring correlation are overly conservative, with observed rates below the nominal level. edgeR including subjects as fixed effects shows a mix of conservative and anti-conservative behavior. Again, MACAU has inflated rates at smaller sample sizes but is overly conservative at *n*=10. The pattern for limma is similar to the between-subject test, with observed rates slightly inflated at *n*=3, but worsening behavior with increasing sample size. MCMSeq and LMM were consistently closest to nominal levels out of all the methods. Again, increasing sample size typically improved performance across all methods (limma being the noticeable exception), and brings the type 1 error rates very close to nominal levels for the MCMSeq and LMM methods. Overall, similar results were seen for the interaction term.

The results for CPGLMM and VarSeq are presented only in the supplementary materials as the results for the CPGLMM were virtually identical to the NBGLMM, and VarSeq either found no significant features or had highly inflated error rates.

#### False discovery rates

**Fig. 3:**
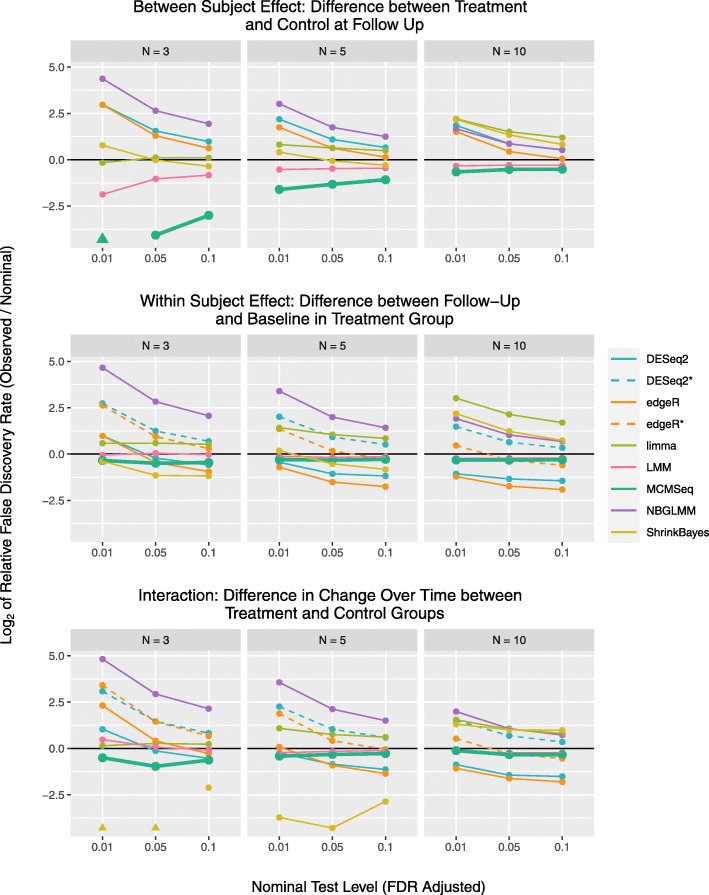
Relative FDRs for the 3 tests of interest. Relative rates are plotted on the log2 scale, for the 3 different testing thresholds at each of the 3 sample sizes examined. Values greater than 0 indicate FDRs higher than nominal levels (e.g. a value of 1 means the observed rate was double what one would expect), while values less than 0 indicate conservative behavior (e.g. -1 means observed rate was half of what is expected). Values in the figure are based on the average rate calculated across the 10 data sets simulated at each sample size. Triangular plotting symbols indicate that no differentially expressed genes were identified at the given threshold

Figure 3 compares the relative FDR (again averaged across the 10 simulated data sets) between the methods. In general, the patterns of behavior for FDRs mimic what was observed for type 1 error rates; if a method has inflated type 1 error rates, it also has inflated FDRs. Also similar to the type 1 error results, most methods tend to be closer to the nominal rates with increasing sample size (also see Supplementary Figs. 7-8 and Supplementary Table 5).

When testing within-subject and interaction effects, DESeq2 and edgeR ignoring correlation are conservative, especially at the larger sample sizes. However, when including subjects as fixed effects, DESeq2 has severely inflated FDRs, while edgeR displays inflation at *n*=3 per group, but conservative behavior at *n*=10 per group. At all sample sizes and testing thresholds, the observed FDRs for the NBGLMM were inflated. ShrinkBayes is conservative at small sample sizes, but has FDR inflation at 10 subjects per group, showing worse performance with increasing sample size. MACAU has similar behavior to its type 1 error profile, with FDR inflation at *n*=3 and very conservative behavior at the largest sample size. Again, limma performs best at *n*=3 and has worsening FDR inflation with increasing sample size. The LMM and MCMSeq again came closest to the nominal levels with both tending to be slightly conservative at the lower sample sizes.

#### Power

**Fig. 4:**
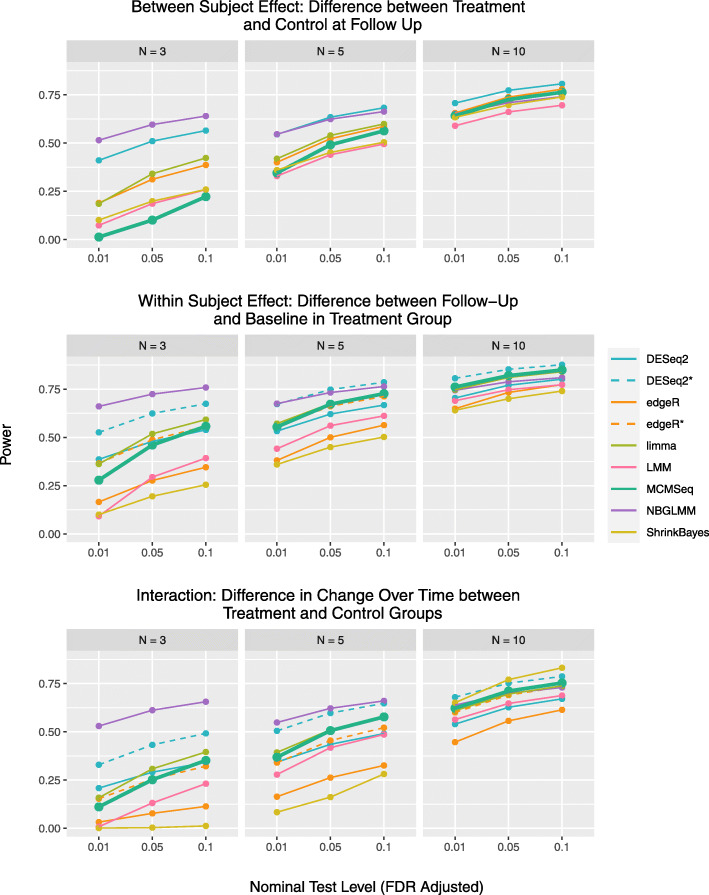
Statistical power (recall) for the 3 tests of interest. Values in the figure are based on the average power calculated across the 10 data sets simulated at each sample size

Power (recall) for the various methods can be seen in Fig. 4 (see also Supplementary Figs. 9-10 and Supplementary Table 6). For between-subject tests with 3 subjects per group, MCMSeq has the lowest power to detect DEGs, which is expected given its conservative performance in terms of FDR and type 1 error rate for between subject effects. However, as sample size increases, MCMSeq’s performance substantially improves, having higher power than the LMM, ShrinkBayes and NBGLMM methods at 10 subjects per group, despite the fact that ShrinkBayes and the NBGLMM have inflated type 1 error and FDRs. At 10 subjects per group, the methods with higher power than MCMseq fail to control type 1 error rates and FDRs at their nominal levels.

For within-subject tests, ShrinkBayes has the lowest power, followed by edgeR ignoring correlation and the LMM. The conservative behavior of edgeR may be driven by failing to account for the paired structure of the data, while the poor performance of the LMM may be driven by the large number of models that failed to converge. For the interaction test, at 3 and 5 subjects per group, ShrinkBayes has the lowest power, but at N=10 per group has the highest power (though FDR was also very inflated). The power for MACAU remains relatively constant across the various sample sizes, increasing only marginally from *n*=3 to *n*=10. For limma, power is typically about average for all of the methods, in spite of having significant type 1 error and FDR inflation at *N*=5 and *N*=10. For both the within subject and interaction tests, the relative performance of the MCMSeq method improves with increasing sample size. Again, at 10 subjects per group, the only methods with higher power than MCMseq fail to control type 1 error rates and/or FDRs at their nominal levels.

**Fig. 5:**
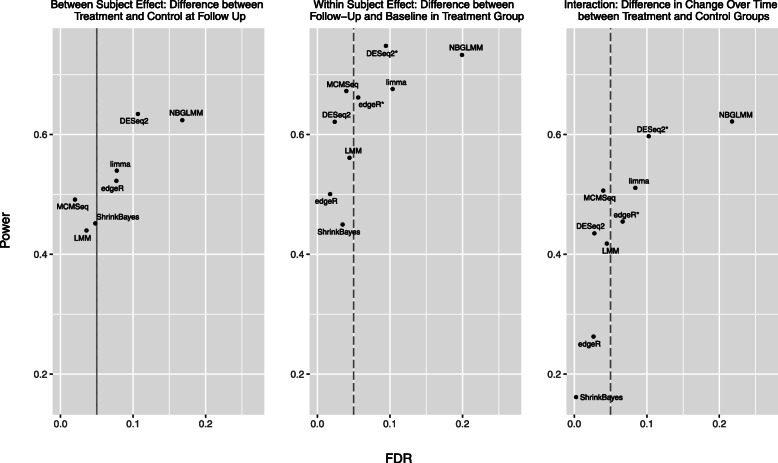
Scatter plot of power versus observed FDR for the 3 contrasts of interest in the *n*=5 simulated datasets. Significance was determined at the 0.05 FDR level. Points that lie to the left of the dashed vertical line represent methods that have an observed FDR less than the nominal rate of 5%, while points to the right represent methods with FDR inflation

Figure 5 shows the relationship between FDR and power for the various methods in the *n*=5 simulated datasets at the commonly used 0.05 FDR level (See Supplementary Fig. 11 for *N*=3 and *N*=10). Here we see that for each type of test, MCMSeq has the highest power of the methods that maintain the nominal FDR level. Again, the only methods that have higher power also have substantially inflated FDRs.

### Real data results

#### Model convergence and stability

The NBGLMM had the largest number of models that failed to converge (≈8.4%), followed by the LMM (≈2.5%). Convergence rates were higher in the TB dataset compared to our simulation studies. This difference is likely due to the larger number of observations (112) and subjects (N=40, median of 3 observations per subject) compared to our simulations, which included a maximum of 40 observations. To explore this, we subsampled 2 observations from 5 subjects in both the Control and Latent groups in the TB dataset to create a scenario similar to our 5 subject per group simulations. We found that convergence rates were similar to our simulation study, with 18.1% of LMMs, 17.6% of NBGLMMs, 0.4% of DESeq models, and <1*%* of MCMSeq models failing to converge. For MCMSeq, we also ran the sampler four separate times to evaluate the consistency of the results, as this method is based on a stochastic estimation process. In general, we found the results were highly consistent between the multiple runs, as the Spearman correlation of *p*-values was at least 0.9996 between any combination of runs, and typically over 99% of differential expression calls for the contrasts we tested were congruent. More details can be found in Supplementary Tables 10 and 11.

#### Number of significant genes

For brevity, we focus our discussion on the edgeR, edgeR*, limma, LMM, MCMSeq, NBGLMM and ShrinkBayes methods; however, full results for all methods can be found in the Supplementary materials (Supp. Table 9). CPGLMM results were similar to the NBGLMM. VarSeq and MACAU did not find any differentially expressed genes for any of the contrasts testing for changes over time or differences in changes over time between the groups. The numbers of significant genes at the 0.05 FDR level identified using each analysis method are presented in Table 5. For between-subject contrasts, such as differences between groups at baseline, edgeR and ShrinkBayes found the highest number of significant genes, followed by the NBGLMM and limma. This is not surprising as these methods had inflated FDR for between subject effects in our simulations, even at the largest sample size. LMM and MCMSeq found over 50% fewer DEGs between the TBP and LTBI and TBP and control groups compared to these methods. Previous analyses of a 20 gene TB risk score showed no or small differences between TBP and LTBI and TBP and control groups at baseline [1].

**Table 5:** Number of significant genes by contrast in the tuberculosis dataset. 15,993 genes were included in the analysis. Genes were called significant if the FDR adjusted *p*-value was <0.05

	edgeR	edgeR ^∗^	limma	LMM	MCMSeq	NBGLMM	ShrinkBayes^1^
Number Failed to Converge	0	0	0	392	132	1348	0
**Baseline Differences**							
Latent vs. Control	279	-	11	2	3	9	416, 407
Progressor vs. Control	2221	-	1009	752	650	1130	1821, 1013
Progressor vs. Latent	1643	-	552	299	256	667	1369, 708
**Changes Over Time**							
Control	1	0	0	0	0	0	2977
Latent	2	0	0	0	3	2	3171
Progressor	664	790	2460	1413	1463	1878	7848
**Differences in Changes**							
**Over Time**							
Latent vs. Control	0	0	0	0	3	0	3331, 3230
Progressor vs. Control	16	272	310	537	635	794	7199, 6429
Progressor vs. Latent	170	278	1852	825	852	1328	6681, 7390

^1^In order to test all contrasts of interest, ShrinkBayes models were fit three times, using a different reference group each time. This resulted in some contrasts being evaluated twice. As ShrinkBayes is a stochastic method, the number of significant genes can differ between model fits. We present the results from both models when tests were performed multiple times

For within-subject tests of changes in gene expression over time, changes in gene expression were expected in the TBP group, which developed active TB over the course of the study. Expression was expected to be relatively stable in the control and LTBI groups. As expected, most methods identified few or no genes that significantly changed over time in both the control and LTBI groups. However, ShrinkBayes found nearly 3,000 genes with significant changes over time in these groups. For the TBP group, ShrinkBayes found the largest number of significant genes, with over 5,000 more DEGs than NBGLMM and limma, which had the next highest numbers. In our simulation studies, the NBGLMM and limma had inflated FDRs, even at 10 subjects per group. The large number of additional significant genes found with ShrinkBayes may reflect the pattern of increased FDR with increasing sample sizes noted in the simulation study. For the TBP group, edgeR identified fewer genes than the MCMSeq and LMM methods. edgeR ignoring correlation identified fewer genes than when subjects were included as fixed effects, which also aligns with our simulation results showing that failing to account for correlation caused DESeq2 and edgeR to produce conservative results for tests of within-subject effects. The results for the interaction effects (differences in change over time between groups) largely follow the same patterns as the within subject tests for change in gene expression over time.

We also calculated pairwise Jaccard indices and Kappa agreement statistics to compare the similarity of differential expression calls between methods (Supplementary materials Section 3.2). MCMSeq had the highest agreement with LMM, followed by CPGLMM and NBGLMM. MCMSeq had the lowest agreement with VarSeq and MACAU, which did not find any genes with significant changes in expression over time or with differential changes over time between groups.

#### Enrichment analysis

Results from MCMSeq for the top GO terms are presented in Table 6 and full results can be found in the Supplementary materials as a.csv file. We found 575 significantly enriched GO terms at the 0.05 FDR level. After filtering, 308 GO terms were identified as significantly enriched. The results were enriched for genes relating to immune response, pathways associated with myeloid cell function or activation, T and B cell activation and more general inflammation processes, and both Type I interferon and interferon gamma signaling. Similar results have been reported in cross sectional studies of TB infection [1*,*^50^*,*^51^]. In addition, the MCMSeq results for longitudinal changes in expression in the TBP group were enriched for genes associated with active TB infection in cross-sectional studies, indicating that expression of many of these genes is also changing within subjects as they progress from latent to active TB.

**Table 6:** Functional enrichment analysis

	Description	N Genes	N Genes	N	Fold
		in Set	in DEG List	Expected	Enrichment
**GO Terms**					
0019221	cytokine-mediated	659	110	42.28	2.6
	signaling pathway				
0043312	neutrophil degranulation	483	88	30.99	2.84
0045088	regulation of innate	434	75	27.84	2.69
	immune response				
0001817	regulation of cytokine	676	98	43.37	2.26
	production				
0002684	positive regulation of	1125	137	72.17	1.9
	immune system process				
0010608	post-transcriptional	512	77	32.85	2.34
	regulation of gene				
	expression				
0016032	viral process	690	93	44.27	2.1
1903706	regulation of hemopoiesis	442	69	28.36	2.43
0051248	negative regulation of	1087	128	69.74	1.84
	protein metabolic process				
0033554	cellular response to stress	1662	175	106.63	1.64
0046649	lymphocyte activation	369	60	23.67	2.53
0002683	negative regulation of	434	66	27.84	2.37
	immune system process				
0034660	ncRNA metabolic process	542	76	34.77	2.19
0034341	response to interferon-	182	38	11.68	3.25
	gamma				
**Previously Published Gene Sets**					
Blankley		327	123	30.4	4.1
Kafouru		18	13	1.7	7.8
Zak		14	9	1.3	6.9

Top GO terms were selected as follows: the top 50 GO terms were selected by *p*-value

The list was then reduced to include only the most specific subclass in an ontology

All results included had BH adjusted *p*-values <0.0001

## Discussion

Longitudinal RNA-Seq experiments have the potential to be a powerful tool for understanding how transcriptional changes that occur over time are related to disease progression, changes in treatment, or changes in environmental exposures. However, currently available RNA-Seq specific analysis tools are not equipped to handle the modeling requirements necessary for these types of data. Of the methods we tested, the LMM and MCMSeq performed best in terms of maintaining type 1 error rates and FDRs at or near their nominal levels for tests of between-subject, within-subject, and interaction effects. In general, MCMSeq tended to be slightly more conservative than the LMM. At higher sample sizes both methods neared nominal rates. Even though the LMM and MCMSeq methods had similar type 1 error rates at 10 samples per group, MCMSeq had much higher power. One concern using the LMM is the large number of models that failed to converge in our simulation study, even after filtering out lowly expressed genes.

Of the methods tested that explicitly model RNA-Seq counts, only MCMSeq was able to combine both good statistical power with control of error rates at the nominal levels. In contrast, using both edgeR or DESeq2, which are not designed to handle repeated measures, lead to sub-optimal performance in terms of low power (ignoring correlation in within-subject and interaction tests) or inflated type 1 error rates and FDRs (including subjects as fixed effects). While limma performed relatively well at small sample sizes, false discovery and type 1 error rates became increasingly inflated as sample size increased, having one of the highest levels of error inflation in our *N*=10 simulations. VarSeq, a model-free method based on a variance component score test that can accommodate repeated measures, was validated for testing gene sets rather than individual genes. In our simulations, it alternated between having low power to detect differential expression and having very inflated error rates, depending on the combination of contrast tested and sample size. MACAU had inflated false discovery and type 1 error rates at *N*=3, but become overly conservative at larger sample sizes.

Both the NBGLMM and CPGLMM consistently had highly inflated type 1 error rates and FDRs when fit under a frequentist paradigm. This could, in part, be a result of the statistical tests for these models being designed for larger sample sizes, while many of the other RNA-Seq specific methods we examined have some modifications due to the expectation of low sample sizes. Lastly, ShrinkBayes is similar to MCMSeq in that both take a Bayesian approach to fitting hierarchical NB models. However, ShrinkBayes had mixed behavior, with a lack of power at the smallest sample size investigated that transitioned into inflated FDRs at the largest sample size. The Bayesian FDR used in ShrinkBayes was originally validated in a simulation using a 2 group comparison with N=8 per group, without correlation or repeated measures [23]. While the Bayesian FDR may perform well in this scenario, it did not maintain nominal FDRs in our simulations with paired data, varying sample sizes, and differing effect sizes. Additionally, as others have noted, we encountered installation and memory issues when attempting to run ShrinkBayes on our personal computers (Windows desktop, Mac Pro desktop, MacBook Pro laptops) [22]. We were able to get the method to run on a Linux HPC, but were forced to break the analysis up into smaller chunks. Implementation of the ShrinkBayes method was non-trivial given its computationally intensive nature, the large number of modeling options, and the limited documentation for the software package.

MCMSeq did struggle with power at the smaller sample sizes for the between-subject test. This is not unexpected as this type of test does not require the extra complexity of the mixed model since there are no repeated measures involved in this contrast. Of the methods examined, MCMSeq was best able to adequately control type 1 error rates and FDRs while maintaining good power for the tests that involved changes over time.

The results from applying the various methods to the TB data set largely mirror the behavior observed in the simulations as the methods that generally had inflated error rates tended to find the largest number of significant features (e.g. CPGLMM, NBGLMM). Methods that showed conservative behavior at larger sample sizes, such as VarSeq and MACAU, failed to find any genes that significantly changed over time. For the two methods that were best able to maintain type 1 error rates in our simulation study (MCMSeq and the LMM), MCMSeq found more significant genes for tests of changes over time and differences in changes over time between groups, which aligns with the results of our simulation study, where MCMSeq had more power than the LMM.

In our application, ShrinkBayes was an outlier, especially for the “changes over time” and “differences in changes over time” contrasts. For changes over time in both the control and latent groups, all of the other methods find no or very few genes showing significant changes. However, ShrinkBayes finds about 3,000 genes for both tests. The same is true for comparing the change over time in the latent group to the change over time in the control group. ShrinkBayes also finds several thousand more significant genes than all the other methods for changes over time in the TB progression group. This could be a continuation of the behavior seen in the simulation study where ShrinkBayes had significant FDR inflation at the largest sample size, which is smaller than the number of subjects in the TB dataset. However, as others have noted, ShrinkBayes produces a large number of integrated nested Laplace approximation (INLA) errors during model fitting which the ShrinkBayes documentation instructs users to ignore [22]. Therefore, it is also possible that these anomalous results are due to problems in model fitting. ShrinkBayes also showed inconsistent results in that the number of significant genes for a given test depended on the parametrization. For example, testing for baseline differences between treatment and control groups in the TB data resulted in 963 significant genes for one parametrization and 1,561 for a different parametrization, a relative difference of about 50%.

While fitting the NBGLMM using MCMC in MCMSeq is computationally intensive and run times are significantly longer than using either edgeR or DESeq2 (1.4 and 2.3 minutes respectively to analyze all contrasts for a single *n*=10 simulated data set), our implementation available in the mcmseq R package (196 min) was actually faster than fitting the frequentist NBGLMM (803 min) or the CPGLMM (492 min). It is also faster than ShrinkBayes (474 min), which uses Laplace approximations rather than MCMC, but involves computationally expensive steps needed to estimate the more complicated priors utilized in their model. Moreover, the fitting function in mcmseq allows the user to specify contrasts so as to test combinations of the regression parameters. The frequentist NBGLMM and CPGLMM both had to be fit multiple times to test all of the contrasts we were interested in for both the relatively simple design of the simulated data sets and the more complicated study design of the TB data. ShrinkBayes also suffers from this issue and had to be fit twice for the simulations and three times for the TB data, leading to much longer run times to complete the whole analysis. Additionally, due to the way genes are analyzed and summarized in parallel, MCMSeq does not require large amounts of RAM to be run. For example, the analysis of the TB dataset required only around 300 MB of RAM at its peak consumption (See Supplementary Section 2.3.8 for more information).

In general, our results are consistent with another recently published methodology for analyzing RNA-Seq data with repeated measures [52]. Their strategy largely mirrors our use of the VST paired with fitting normal LMMs, with the major differences being the transformation used (VOOM versus the VST) and the specification of the correlation structure (auto-regressive versus compound symmetric as implied by the use of a random intercept in our implementation). They also found that methods that do not account for the correlation induced by repeated measures could not match those that do.

One limitation of the current implementation of the mcmseq R package is that only a single random intercept can be included in the models. While a random intercept is a relatively simple treatment of the correlation structure, it is a quite commonly used convention in the face of repeated measures, especially when the number of repeated measures is small. While we recognize this is a limitation of our current implementation, accounting for correlation even with a simple random intercept is an improvement over models that do not account for correlation. We plan to add the capability to include a random slope in the near future and will investigate auto-correlation. Accounting for auto-correlation in our GLMM framework will be a non-trivial extension, since unlike linear mixed models for normally distributed outcomes, there is not a covariance matrix for the errors and correlation must be accounted for through specially constructed random effect priors [53].

Model mis-specification is an issue for all RNASeq analysis methods, as omission of important fixed or random effect covariates or incorrect distributional assumptions can impact testing characteristics. Others have investigated the impact of model mis-specification in log-linear generalized linear mixed models for count data in a frequentist context, and found that under-specification of the random effects can result in underestimated standard errors for fixed effects [54]. The magnitude of the underestimation is dependent on several factors, including the sample size, random slope variance and the number of repeated measurements per subject, with the amount of underestimation increasing with increasing variance and number of repeated measurements. In studies with a small samples size and few repeated measurements, as is common in RNA-Seq, there is limited information to estimate complex random effect or covariance structures, such that using a simpler (e.g. random intercept only) random effects structure may not have a substantial influence on inference.

While in the future we intend to extend the package to allow for multiple levels of clustering and additional random effects, such as a random slope, the current version should prove adequate for many studies with repeated or paired measurements. We also hope to add features allowing users to simultaneously test multiple fixed effects regression parameters (i.e. ANOVA type tests). Another limitation is the precision with which the MCMC *p*-value (i.e. contour probability) can be calculated. Since a Monte Carlo estimate is used to compute the tail probability, the precision is determined by the number of MCMC iterations that are run. We found that chains of length 30k were generally sufficient for type 1 error and FDR control, but if greater accuracy is needed the only solution is to run longer chains. Using normal approximations for the posteriors of regression coefficients was investigated, but this strategy was unable to maintain nominal error rates, even at *n*=10. We also explored using Integrated Nested Laplace Approximations (INLA) as an alternative approach to fitting the Bayesian hierarchical model utilized in MCMSeq. Using the INLA R package we found that both type 1 errors and FDR rates were inflated, similar to the inflation we noted when using normal approximations based on the MCMC output. This is not surprising as INLA is an approximate method as compared to MCMC, which is asymptotically exact. While the custom sampler implemented in the mcmseq R package was faster than INLA at the sample sizes we tested, we are interested in further exploring both INLA and variational methods in future work. Though our motivation for developing MCMSeq was to create a tool for analyzing longitudinal RNA-Seq experiments, we have also included functions in the mcmseq R package that can fit NBGLMs that do not include random effects. In a similar set of simulations that did not include random effects, we found that using MCMC to fit a NBGLM gave similar improvements for maintaining nominal FDRs and type 1 error rates, likely due to incorporating uncertainty in the dispersion parameter into the posteriors of the regression coefficients (data not shown).

## Conclusions

Failing to account for repeated measurements when analyzing RNA-Seq experiments can result in significantly inflated false positive and false discovery rates. The most used RNA-Seq analysis software cannot inherently account for repeated measures, and though some workarounds have been proposed, our simulations show they are not particularly effective. Ultimately, the Bayesian hierarchical model underlying MCMSeq was best able model RNA-Seq expression from study designs with repeated/clustered measurements based on its ability to combine good power with maintenance of nominal false positive and false discovery rates. Additionally, MCMSeq’s NBGLMM framework is able to flexibly model mean expression with the inclusion of covariates, and it can handle arbitrarily many repeated measurements which can also vary in number between individuals. Finally, the implementation found in the mcmseq R package makes utilizing Bayesian NBGLMMs more accessible by automating and parallelizing the process of fitting and summarizing results from these models over the tens of thousands of genes typically analyzed in an RNA-Seq experiment.

## Supplementary information

**Additional file 1** Supplementary materials. Supplementary methods, results, tables, and figures.

## Data Availability

Datasets used in the simulation studies are available from the corresponding author upon request. The real RNA-Seq data was originally published in [1], and was downloaded for this application from the GEO DataSets website (GEO Datasets: GSE107994, GSE107993)

## Abbreviations

ANOVA: Analysis of Variance
CPM: Counts per Million
CP: Compound Poisson
CPGLMM: Compound Poisson Generalized Linear Mixed Model
DEG: Differentially Expressed Gene
FDR: False Discovery Rate
GO: Gene Ontology
GLM: Generalized Linear Model
GLMM: Generalized Linear Mixed Model
IGRA: interferon-gamma (IFN- *γ*) release assay
INLA: Integrated Nested Laplace Approximation
LTBI: Latent Tuberculosis Infection
LMM: Linear Mixed Model
LOESS: Locally weighted polynomial regression
MCMC: Markov-chain Monte Carlo
NB: Negative Binomial
NBGLMM: Negative Binomial Generalized Linear Mixed Model
NBGLM: Negative Binomial Generalized Linear Model
RAM: Random Access Memory
RNA-Seq: RNA Sequencing
TB: Tuberculosis
TBP: Tuberculosis Progressor
WLS: Weighted Least Squares
