# Supplementary material for: MCMSeq: Bayesian hierarchical modeling of clustered and repeated measures RNA sequencing experiments
Source: BMC Bioinformatics. 2020 Aug 28;21:375. doi: 10.1186/s12859-020-03715-y (PMC7455910; doi:10.1186/s12859-020-03715-y)
Supplement: Supplementary file 1 — Additional file 1 Supplementary materials. Supplementary methods, results, tables, and figures. [file 12859_2020_3715_MOESM1_ESM.pdf]

MCMSeq: Bayesian Hierarchical Modeling of Clustered and Longitudinal RNA Sequencing Experiments

Supplementary Materials

1 Methods

Table 1: Methods Comparison: Key features of different analysis methods.

	MCMSeq	CPGLMM	DESeq2	DESeq2*	edgeR	edgeR*	limma	LMM	MACAU	NBGLMM	ShrinkBayes	VarSeq
Count based model	✓	✓	✓	✓	✓	✓		✓		✓	✓	✓
Accounts for repeated measures	✓	✓					✓	✓	✓	✓	✓	✓
Allows correlation to vary by gene	✓	✓						✓		✓	✓	✓
RNASeq analysis package available	✓		✓	✓	✓		✓		✓		✓	✓
Fit models once to perform testing	✓		✓	✓	✓	✓	✓	✓				
Fit simulated dataset of 15000 genes in < 10 minutes			✓	✓	✓	✓	✓	✓				✓
Converges for > 95% of genes in all simulation scenarios	✓	✓	✓	✓	✓	✓	✓		✓	✓	✓	✓
Maintains nominal FDR for all simulation scenarios	✓							✓				

1.1 MCMC Sampler

Let $\boldsymbol{y}_g = \{y_{g1,1} \dots y_{gij} \dots y_{gI,n_I}\}'$ be the vector of counts for gene g, $\boldsymbol{X} = \{X_{1,1}, \dots X_{I,n_I}\}'$ be the fixed effects design matrix, and $\boldsymbol{Z} = \{Z_{1,1}, \dots Z_{I,n_I}\}'$ be the random effects design matrix, where $i \in \{1 \dots I\}$ indexes subject and $j \in \{1 \dots n_i\}$ indexes the repeated observations for subject i. Let $f(\boldsymbol{y}|\boldsymbol{\beta}_g, b_1, \dots b_I, \alpha_g)$ be the negative binomial likelihood. Given the current set of values at iteration s of the MCMC algorithm, a set of values for the $s+1$ iteration is simulated as follows:

1. Jointly update the fixed and random effects coefficients, $\boldsymbol{\beta}_g$ and $\boldsymbol{b}_g = \{b_{g1} \dots b_{gI}\}'$, using a Metropolis-Hastings step with a weighted least squares proposal.

   - Calculate $\tilde{\boldsymbol{y}}_g = \log(\boldsymbol{\mu}_g^{(s)}) + \frac{\boldsymbol{y}_g - \boldsymbol{\mu}_g^{(s)}}{\boldsymbol{\mu}_g^{(s)}}$ and $\boldsymbol{W}_g^{-1} = \alpha_g^{(s)} + \frac{1}{\boldsymbol{\mu}_g^{(s)}}$. Note that $\boldsymbol{\mu}_g^{(s)} = \exp(\boldsymbol{X}\boldsymbol{\beta}_g^{(s)} + \boldsymbol{Z}\boldsymbol{b}_g^{(s)})$.

   - The proposal distribution for the coefficients is multivariate normal with mean M and covariance C, where $M = (\boldsymbol{R}_0^{-1} + [\boldsymbol{X}, \boldsymbol{Z}]'\boldsymbol{W}[\boldsymbol{X}, \boldsymbol{Z}])^{-1}([\boldsymbol{X}, \boldsymbol{Z}]'\boldsymbol{W}\tilde{\boldsymbol{y}})$ and $C = (\boldsymbol{R}_0^{-1} + [\boldsymbol{X}, \boldsymbol{Z}]'\boldsymbol{W}[\boldsymbol{X}, \boldsymbol{Z}])^{-1}$. $\boldsymbol{R}_0$ is a $(p+I)$ x $(p+I)$ diagonal covariance matrix with s^2, the prior mean for the fixed effects, on the first p elements of the diagonal, and Σ_g, the random effect variance, on the remaining I elements of the diagonal.

   - $\boldsymbol{\beta}_g^*$ and $\boldsymbol{b}_g^*$ are drawn from the proposal distribution, and the acceptance probability is calculated as:

   $$a\{(\boldsymbol{\beta}_g^*, \boldsymbol{b}_g^*), (\boldsymbol{\beta}_g^{(s)}, \boldsymbol{b}_g^{(s)})\} = \frac{f(\boldsymbol{y}|\boldsymbol{\beta}_g^*, \boldsymbol{b}_g^*, \dots)p(\boldsymbol{\beta}_g^*)p(\boldsymbol{b}_g^*)q(\boldsymbol{\beta}_g^{(s)}, \boldsymbol{b}_g^{(s)})}{f(\boldsymbol{y}|\boldsymbol{\beta}_g^{(s)}, \boldsymbol{b}_g^{(s)}, \dots)p(\boldsymbol{\beta}_g^{(s)})p(\boldsymbol{b}_g^{(s)})q(\boldsymbol{\beta}_g^*, \boldsymbol{b}_g^*)}$$

   where $p(\boldsymbol{\beta}_g)$ is the pdf of the multivariate normal prior for $\boldsymbol{\beta}_g$, $p(\boldsymbol{b}_g)$ is the normal prior for the random effects, with mean 0 and variance $\Sigma_g^{(s)}$, and q is the pdf of the multivariate normal proposal distribution with mean M and variance C.

   - γ is then sampled from a $U(0,1)$ distribution. If $a > \gamma$, then $\boldsymbol{\beta}_g^{(s+1)} = \boldsymbol{\beta}_g^*$ and $\boldsymbol{b}_g^{(s+1)} = \boldsymbol{b}_g^*$. Else $\boldsymbol{\beta}_g^{(s+1)} = \boldsymbol{\beta}_g^{(s)}$ and $\boldsymbol{b}_g^{(s+1)} = \boldsymbol{b}_g^{(s)}$.

2. Update $\boldsymbol{\Sigma}_g$, the random intercept variance, by sampling from the conditionally conjugate inverse gamma distribution with parameters $U + \frac{I}{2}$ and $V + \sum_{i=1}^{I} \frac{b_{gi}^2}{2}$ in a

Gibbs step.

3. Update $\log(\alpha_g)$ using a random walk Metropolis Hastings step.

   - Draw $\log(\alpha_g)^*$ from a normal distribution with mean $\log(\alpha_g)^{(s)}$ and variance σ^2_{rw}.
   - The acceptance probability is calculated as:

$$a\{\log(\alpha_g)^{(s)}, \log(\alpha_g)^*\} = \frac{f(\boldsymbol{y}|\log(\alpha_g)^*, \dots)p(\log(\alpha_g)^*)}{f(\boldsymbol{y}|\log(\alpha_g)^{(s)}, \dots)p(\log(\alpha_g)^{(s)})}$$

   where $p(\log(\alpha_g))$ is the pdf of the normal prior for $\log(\alpha_g)$.

   - γ is then sampled from a $U(0,1)$ distribution. If $a > \gamma$, then $\log(\alpha_g)^{(s+1)} = \log(\alpha_g)^*$. Else $\log(\alpha_g)^{(s+1)} = \log(\alpha_g)^{(s)}$.

1.2 MCMSeq P-Values: Contour Probabilities

The MCMC p-values returned as part of the `mcmseq` R package are estimated contour probabilities [1] that a specific parameter is equal to 0. We estimate this quantity using the MCMC sample by first calculating the proportion of MCMC iterations that lie on the opposite side of 0 as the posterior median (Supplemental Figure 1). This tail probability is then multiplied by 2 to get the final estimate. We then treat these values as corollaries to frequentist p-values from two-sided tests that the parameter is equal to 0.

MCMC p-values are twice the posterior probability of the null in a one-sided hypothesis testing framework. Casella and Berger [2] discussed the relationship between one sided posterior probabilities and one-sided p-values. Considering a 1-sided test of $\theta > 0$, for normal priors centered at 0, the infimum of $P(\theta \geq 0|X)$ over all $\mathrm{N}(0, \sigma^2)$ priors is equal to

the 1-sided frequentist p-value, given the condition of monotone likelihood ratio. $P(\theta \geq 0|X)$ will always be greater than or equal to the frequentist one-sided p-value and will approach the p-value from above as the prior variance increases. Therefore, using normal priors, centered at 0 with large variance, $P(\theta \geq 0|X) \approx p(X)$, where $p(X)$ is the 1-sided p-value. 2-sided p-values can be obtained by multiplying the 1-sided p-value by 2. Therefore, $2P(\theta \geq 0|X) \approx 2p(X) =$ 2-sided p-value.

The distribution of p-values from the null genes for all methods tested are displayed in Supplementary Figure 3 (a-b). We expect p-values to have a uniform distribution under the null. The estimated contour probabilities, as obtained from the MCMC sample tail probabilities, are noticeably more uniform than most of the other methods. MCMC p-values behave as frequentist p-values in terms of distribution and type 1 error rates in our simulation studies; therefore performing multiple comparisons adjustments from Benjamini and Hochberg to control FDR is appropriate.

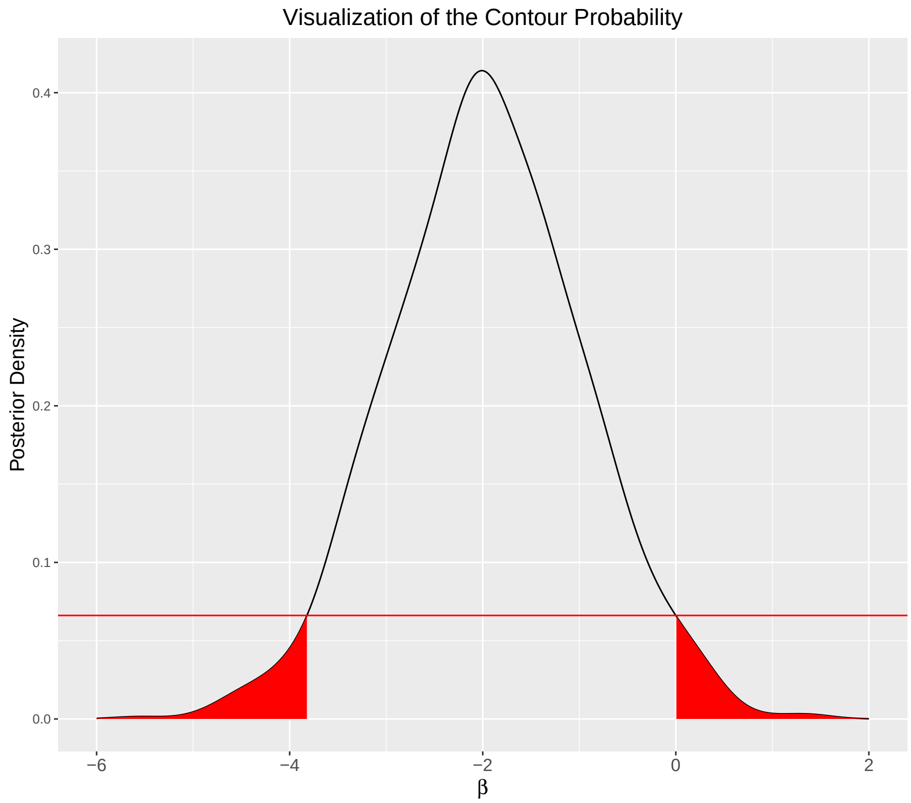

Figure 1: Schematic of the contour probability that $\beta = 0$. The horizontal line is the posterior density at $\beta = 0$, and the contour probability is the cumulative density of the distribution where the density is below this value (highlighted in red).

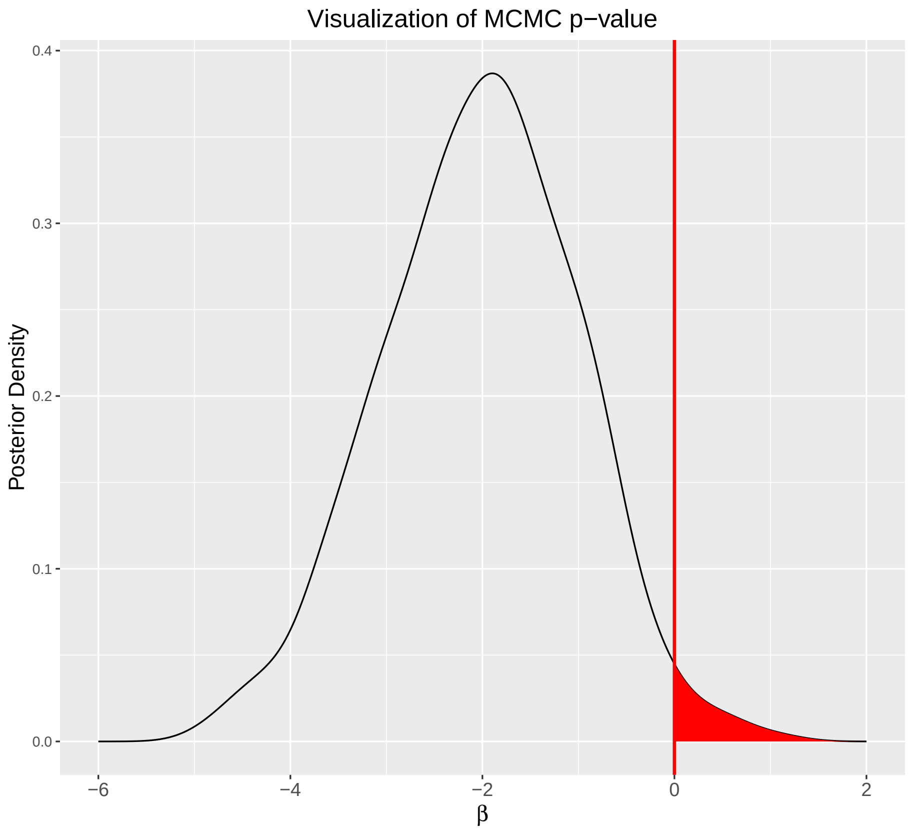

Figure 2: Schematic of the MCMSeq p-value. Two times the highlighted tail probability is used to estimate the contour probability of Box and Tiao.

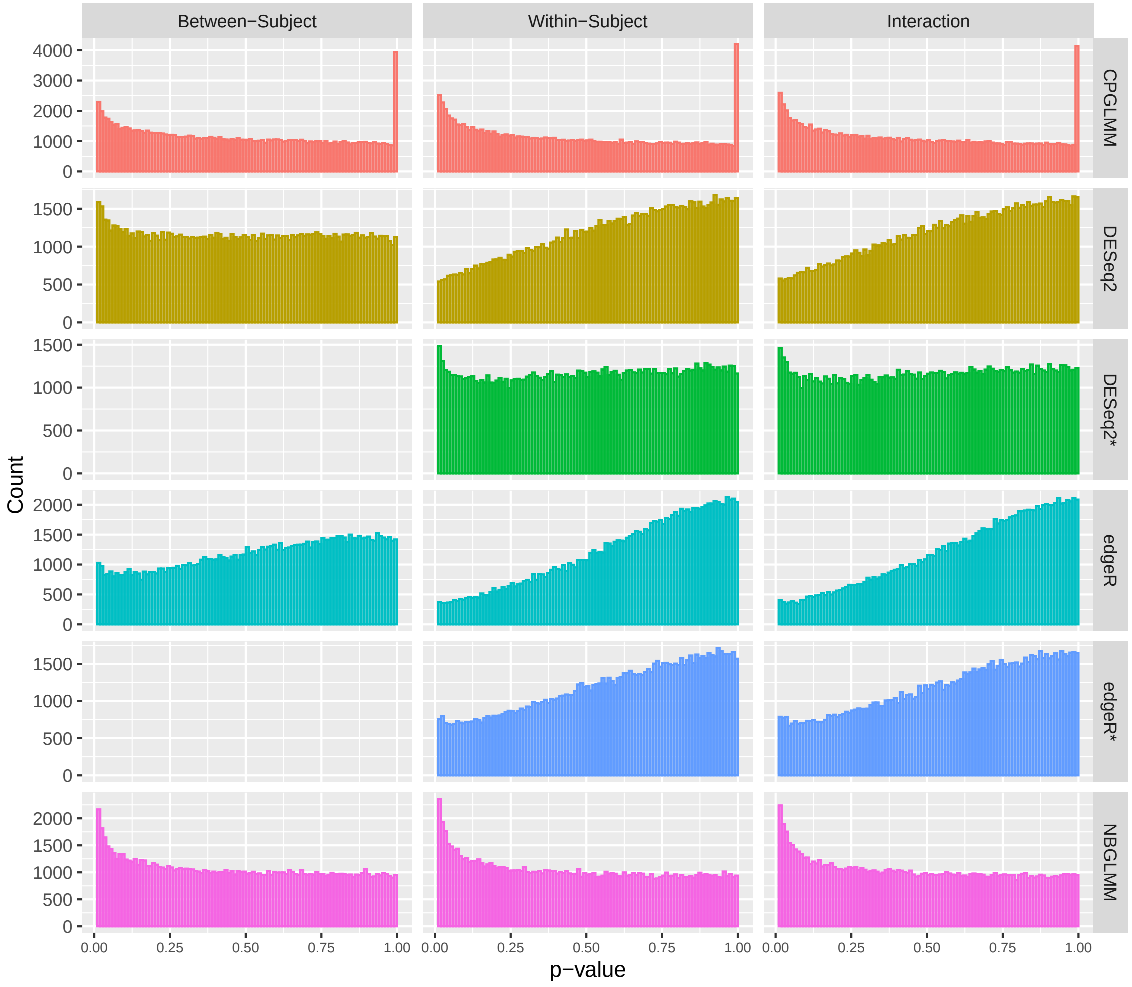

Figure 3: (a) Histograms of p-values for between-subject, within subject and interaction p-values for null features in the $n = 5$ simulated datasets by method. First 6 methods.

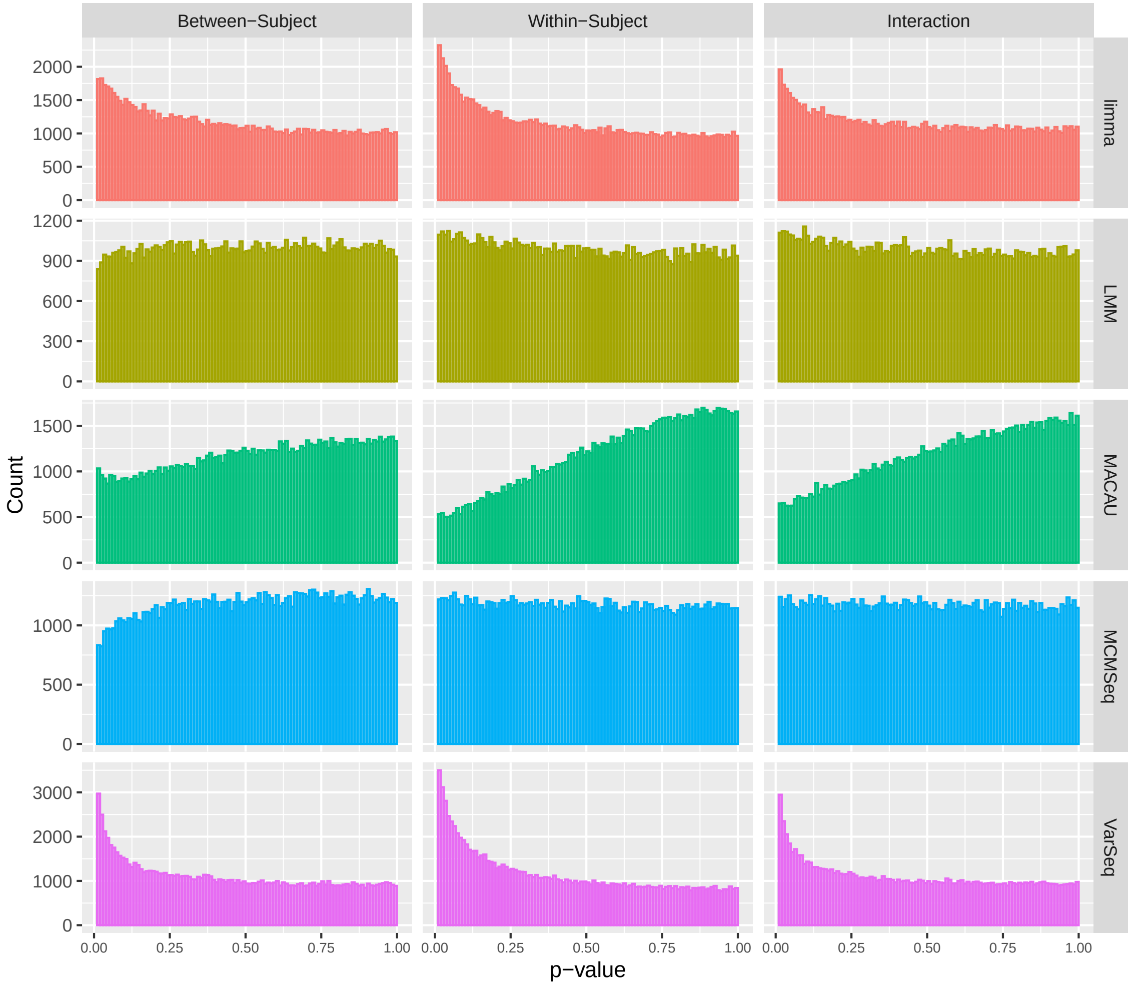

Figure 3: (b) Histograms of p-values for between-subject, within subject and interaction p-values for null features in the $n = 5$ simulated datasets by method. Last 5 methods.

Table 2: Comparison of observed versus expected/nominal FDRs when using the a Bayesian FDR control procedure similar to that presented in Morris et al. (2008) [3]. These results come from the $n = 5$ simulations.

	Nominal FDR	Observed FDR
**Interaction**		
	0.01	0.208
	0.05	0.449
	0.10	0.571
**Between-Subject**		
	0.01	0.169
	0.05	0.414
	0.10	0.545
**Within-Subject**		
	0.01	0.210
	0.05	0.448
	0.10	0.571

1.3 MCMSeq Diagnostics

To flag genes whose MCMC chain may have not converged, we focus on two main diagnostic quantities: acceptance rates and Geweke tests [4]. For the acceptance rates, we recommend a minimum of 10% for each of the model parameters. The Geweke tests essentially perform a t-test comparing the first 20% of the chain to the final 50% of the chain. This is again done for each model parameter, and then the resulting p-values are adjusted for multiple comparisons using the Benjamini-Hochberg method. The default in the `mcmseq` R package is to call any gene with an FDR < 0.05 for any model parameter a potential convergence

failure. More details about these diagnostics can be seen in the `mcmseq` package vignette.

2 Simulation Study

2.1 Data Generation

We simulated datasets using a two group design (e.g. treatment and control) with paired observations (e.g. baseline and follow-up) on each subject. Count data were simulated from a NB distribution as follows:

$$\begin{aligned} Y_{gij} &\sim \mathcal{NB}(\mu_{gij}, \alpha_g) \\ \log(\mu_{gij}) &= \beta_{g0} + \beta_{g1} I_{T_i} + \beta_{g2} I_{F_{ij}} + \\ &\quad \beta_{g3} I_{T_i} I_{F_{ij}} + b_{gi} \\ b_{gi} &\sim \mathcal{N}(0, \sigma^2_{gb}) \end{aligned} \tag{1}$$

where I_{T_i} is an indicator function that equals 1 if subject i is in the treatment group, I_{F_i} is a similar indicator for if observation j is the follow-up, and b_{gi} is the random intercept for gene g and subject i. We assume that for each gene, the subject-specific random effects are normally distributed with mean 0 and variance Σ_g. In this model, $e^{\beta_g 0}$ represents the mean expression of gene g in the control group at baseline, β_{g1} is the log(fold change) comparing the treatment group to the control group at baseline, β_{g2} is the log(fold change) comparing follow-up to baseline within the control group, and β_{g3} is the difference in the change in log expression over time between control and treatment groups.

Values for β_{g0}, α_g, and σ^2_{gb} were drawn from an empirical distribution for triplets of mean counts per million (CPM), dispersion (α_g), and random intercept variance (σ^2_{gb}) observed across human samples in several real RNA-Seq data sets that had repeated mea-

sures [5, 6]. We used an empirical distribution since we observed strong relationships between expression level, dispersion estimates, and random intercept variance estimates from NBGLMM fits performed in the real data, and thus we wanted to maintain this structure in our simulated data sets. The dispersions and random intercept variances were used as is. The 15k mean CPMs drawn for a particular simulated data set were renormalized to add up to 1 million, and were then multiplied by an expected library size (i.e. 30 million). The log of these values were then used for the β_{g0}.

Simulated data sets were generated with a mix of null genes and mixed effect size differential expression. For each of these scenarios, 10 data sets of $\approx 15,000$ genes each (slight deviations due to filtering out low count features) were simulated for three different sample sizes: $n = 3, 5$, and 10 subjects per group. In each dataset, 20% of genes were simulated to have differential expression between baseline and follow-up in the treatment group by setting $\beta_{g1} = \beta_{g2} = 0$ and $\beta_{g3} \neq 0$. In this scenario, there is no difference in expression between the control and the treatment groups at baseline, expression remains stable over time in the control group, expression changes over time in the treatment group, and there is differential expression between the control and treatment groups at follow-up. For these mixed effect size data sets, β_{g3} values were drawn from a Gamma distribution with mode of log(2) and a standard deviation of 0.5, and then each were randomly assigned to be positive or negative (see Supplementary Figure 4 for a histogram of interaction terms generated from this scheme). These were designed to investigate FDR and power in a more realistic setting where there are varying levels of differential expression.

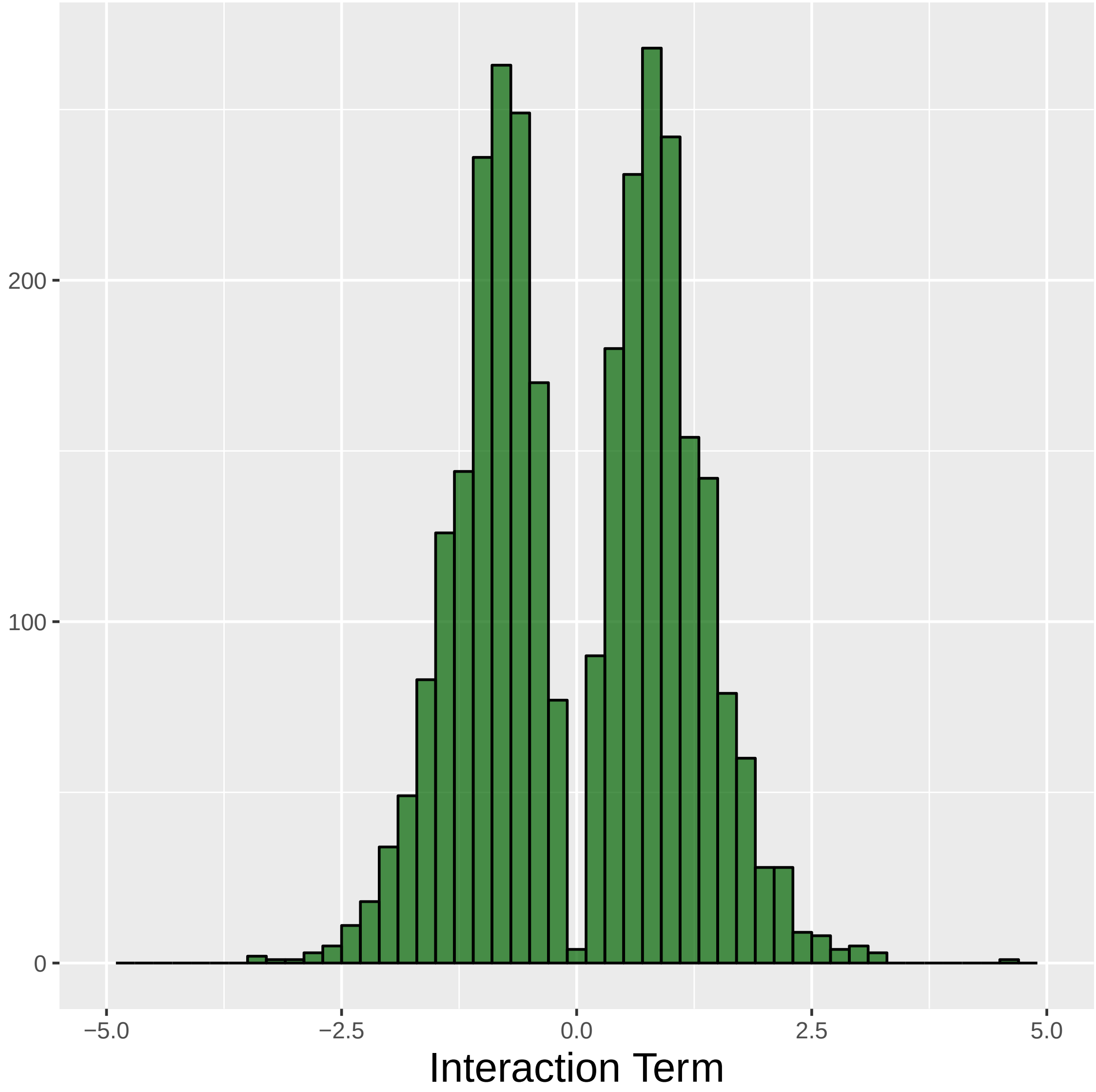

Figure 4: Simulation Study: Interaction Effect Sizes

2.2 Model Fitting Details

MCMSeq

We applied our proposed Bayesian hierarchical model using the implementation available in the `mcmcseq` R package with the following priors:

$$\begin{aligned}
\boldsymbol{\beta_g} &\sim \mathcal{MVN}\left(\begin{bmatrix} m_g \\ 0 \\ 0 \\ 0 \end{bmatrix}, 7^2 \cdot I_4\right) \\
b_{gi} &\sim \mathcal{N}(0, \sigma^2_{gb}) \\
\sigma^2_{gb} &\sim \mathcal{IG}(.01, .01) \\
\log(\alpha_g) &\sim \mathcal{N}(A_g, B).
\end{aligned}$$

where m_g is the log(mean counts) across all samples for gene g and I_4 is a 4 x 4 identity matrix.

To stabilize estimation of the dispersion parameter at small sample sizes, we share information across genes when specifying the log normal prior for the dispersion parameter. First, for each gene, we calculate the average log(CPM) across samples and a naïve method-of-moments estimate of the dispersion parameter. Locally weighted polynomial regression (LOESS) is then used to model the relationship between the log of the method-of-moments dispersion estimates and the average log(CPM). A_g is then set to the expected log(dispersion) at the average log(CPM) for gene g based on the LOESS regression. B is set to $(k \cdot \tau)^2$ where k is an inflation factor and τ is the residual standard deviation from the LOESS fit. The method-of-moments dispersion estimates are over-estimates since they do not take into account fixed or random effects that explain variability in the data. There-

fore, we typically choose $k > 1$ in order to include a larger range of potential dispersion values in our prior. In practice, we have found a k of 2 tends to work well and use $k = 2$ for all analyses presented in this paper.

Results were obtained from chains of length 30,000 (10% discarded as burn in). We investigated sensitivity to various choices of priors and found that this set of relatively non-informative priors gave robust results. To summarize the posterior distributions for each parameter and contrast of interest, we construct a pseudo two-sided p-value by taking 2 times the proportion of the posterior that lies on the opposite side of 0 as the posterior median (Figure 2). Features with poor mixing, identified by having fewer than 10% acceptance probabilities for the update of the fixed and random effects or for α_g, or had Benjamini-Hochberg adjusted p-values <0.05 from Geweke statistics were considered convergence failures.

Frequentist NBGLMM and CPGLMM

NBGLMMs were fit with a likelihood-based frequentest approach using the `glmmADMB` R package [7]. We also explored modeling counts with the compound Poisson distribution, as Rudra et al. found that the CPGLMM was a reasonable alternative to the NBGLMM when modeling clustered RNA-Seq counts in the context of heritability [8]. The compound Poisson distribution belongs to the family of Tweedie distributions and is characterized by three parameters: a mean μ, a dispersion α, and the Tweedie parameter p that satisfies $1 < p < 2$. The compound Poisson has variance equal to $\alpha\mu^p$, which is a slightly more flexible than the NB mean-variance relationship. CPGLMMs were fit using the `cplm` R package[9]. For both NBGLMMs and CPGLMMs, models including the main effects from Equation 1 and a random intercept for subject were fit to each gene. Features that produced warnings or errors during model fitting with the `glmmADMB` R package [7] and `cplm` R

package[9] were considered convergence failures.

DESeq2 and edgeR

We fit the simulated data using edgeR and DESeq2 under two different scenarios. In the first, we ignore the repeated measures and do not attempt to account for the correlation between the two samples from a given individual (i.e. the b_{gi} terms are dropped from Equation 1). In the second scenario, denoted as edgeR* and DESeq2*, we include subjects as fixed effects by replacing the random effects b_{gi} from Equation 1 with fixed effects. This requires removing both the intercept (β_{g0}) and the main effect of treatment (β_{g1}) from the model, as they are not estimable. However, we can still estimate β_{g2}, the main effect of time, and the interaction term β_{g3}, which measures a differential time effect between the two treatment groups. In all cases we use the standard pipeline for analyses in the two packages. Features reported as "NA" in edgeR or DESeq2 results were considered convergence failures.

LMMs on transformed counts

An alternative to working directly with RNA-Seq count data is to transform the integer counts into a continuous measure. Once the counts are transformed they can be used in linear mixed models (LMMs), which assume the outcome data are normally distributed. We explored this alternative using the variance stabilizing transformation (VST) that is implemented in the `DESeq2` R package. LMMs were fit to the transformed data using the `lmerTest` R package [10]. Models including the main effects from Equation 1 and a random intercept for subject were fit to each gene, and then p-values were obtained from t-tests using the Satterthwaite method to calculate degrees of freedom [11]. Features that produced warnings or errors during model fitting or that had singular Hessian matrices

were considered convergence failures.

VarSeq

The VarSeq method from the `tcgsaseq` R package was applied to each dataset, specifying the mean relationship in Equation 1 and using a random intercept for each subject. Raw counts were supplied and defaults were selected for all other parameters. The asymptotic test was used to generate p-values for each parameter of interest.

ShrinkBayes

We implemented the ShrinkBayes method as shown in section 9.5 of the ShrinkBayes vignette, “Code for running ShrinkBayes on very large data sets: simulated example.” While each of our simulated datasets was only 15,000 genes, we could not run the entire ShrinkBayes pipeline or wrapper function on any available personal computer or our high performance cluster (HPC) without first tuning and saving the prior distribution and then breaking the dataset into smaller groups of 5,000 genes for posterior estimation. We used a negative binomial distribution for count data and shrinkage priors for all model parameters involved in statistical tests. Otherwise parameters were set to their default values. In order to test all relevant contrasts, the model had to be re-parametrized and fit a second time on each dataset. Since ShrinkBayes returns Bayesian FDRs rather than p-values, we only evaluate ShrinkBayes in terms of FDR and power. Others have noted similar issues with installation, errors, and memory usage when attempting to run ShinkBayes [12].

limma

Linear regression models were fit using functions from the `limma` R package on transformed data [13] [14]. Counts were voom-transformed (supplying the fixed effects design matrix

from Equation 1), and then the duplicateCorrelation function was applied using subject ID as the blocking factor. The original counts were then voom transformed again, this time supplying the fixed effects design matrix, the duplicate correlation value obtained in the previous step, and the subject ID's as the blocking variable. The duplicateCorrelation function was again applied to the results of this second transformation, and then models were fit with the lmFit function using the second set of transformed counts, the second duplicateCorrelation estimate, and subject ID as the blocking variable. Finally, the empirical Bayes procedure for estimating variances was applied for each coefficient/contrast of interest, and both raw and FDR adjusted p-values were obtained.

MACAU

MACAU fits a Poisson GLMM with two random effects, one that accounts for correlation between individuals (based on population structure), and a second that effectively models overdispersion that is not accounted for by sample non-independence [15]. The general covariance structure of the random effects associated with population structure is denoted by the matrix K, and it is assumed to be fixed and known for the model fits. To use MACAU in this scenario, we followed what was done in Example 3 of the original MACAU paper where the relatedness matrix K is estimated using RNA-Seq data rather than using genotypes [15]. Here, expression for each gene was centered and scaled (i.e. mean 0 and unit Std. Dev.), and then the sample covariance matrix was estimated. This was then supplied to the fitting function in the `MACAU2` R package as the known relatedness matrix. Since MACAU only allows for testing of a single coefficient at a time, the model had to be re-fit (and potentially re-parameterized) multiple times to get estimates for all quantities of interest using the fixed effects implicit from Equation 1. The estimated p-values provided by the fitting function were then adjusted to obtain FDRs using the p.adjust function.

2.3 Detailed Results

2.3.1 Convergence

Table 3: Simulation Study: Comparison of Features that Failed to Converge vs. Features that Converged in the simulations. Results are presented as Median (Interquartile Range).

Method	N	Intercept		Dispersion		Random Intercept Variance		Proportion DE		Number of Zeros		Max. Number of Repeated Values		Number of Unique Values	
		Convergence		Convergence		Convergence		Convergence		Convergence		Convergence		Convergence	
		Yes	No	Yes	No	Yes	No	Yes	No	Yes	No	Yes	No	Yes	No
DESeq2	3	6.2	4.5	0.1	1	0	4.4	0.2	0.21	0	0	1	2	12	11
		(5, 7.2)	(3.9, 5.2)	(0, 0.2)	(0.4, 1.5)	(0, 0.1)	(1.1, 7.5)			(0, 0)	(0, 2)	(1, 1)	(1, 2)	(12, 12)	(11, 12)
DESeq2	5	6.2	4.4	0.1	1.1	0	6	0.2	0.18	0	2	1	2	20	18
		(4.9, 7.2)	(3.8, 4.8)	(0, 0.2)	(0.6, 1.6)	(0, 0.1)	(2.8, 8.3)			(0, 0)	(0, 3)	(1, 2)	(2, 3)	(19, 20)	(16, 19)
LMM	3	6.2	6.1	0.1	0.1	0.1	0	0.2	0.2	0	0	1	1	12	12
		(4.9, 7.2)	(4.9, 7.1)	(0, 0.1)	(0, 0.2)	(0, 0.1)	(0, 0.1)			(0, 0)	(0, 0)	(1, 1)	(1, 1)	(12, 12)	(12, 12)
LMM	5	6.2	6	0.1	0.1	0.1	0	0.2	0.2	0	0	1	1	20	20
		(4.9, 7.2)	(4.8, 7.1)	(0, 0.1)	(0.1, 0.3)	(0, 0.1)	(0, 0.1)			(0, 0)	(0, 0)	(1, 2)	(1, 2)	(19, 20)	(19, 20)
LMM	10	6.2	5.9	0.1	0.1	0.10	0	0.2	0.21	0	0	2	2	39	38
		(4.9, 7.2)	(4.7, 7)	(0, 0.2)	(0.1, 0.3)	(0, 0.1)	(0, 0.1)			(0, 0)	(0, 0)	(1, 2)	(1, 2)	(36, 40)	(35, 40)
NBGLMM	3	6.1	7.6	0.1	0	0.1	0	0.2	0.2	0	0	1	1	12	12
NBGLMM		(4.9, 7.1)	(6.9, 8.4)	(0, 0.2)	(0, 0.1)	(0, 0.1)	(0, 0)			(0, 0)	(0, 0)	(1, 1)	(1, 1)	(12, 12)	(12, 12)
NBGLMM	5	6	7.5	0.1	0	0.1	0	0.2	0.19	0	0	1	1	20	20
NBGLMM		(4.8, 7.1)	(6.8, 8.3)	(0, 0.2)	(0, 0.1)	(0, 0.1)	(0, 0)			(0, 0)	(0, 0)	(1, 2)	(1, 1)	(19, 20)	(20, 20)
NBGLMM	10	6	7.5	0.1	0	0.1	0	0.2	0.18	0	0	2	1	38	40
NBGLMM		(4.8, 7)	(6.9, 8.3)	(0, 0.2)	(0, 0)	(0, 0.1)	(0, 0)			(0, 0)	(0, 0)	(1, 2)	(1, 2)	(35, 40)	(39, 40)
MCMSeq	3	6.2	4.4	0.1	1.3	0	5.4	0.2	0.22	0	2	1	2	12	11
MCMSeq		(4.9, 7.2)	(3.8, 4.9)	(0, 0.2)	(0.8, 1.8)	(0, 0.1)	(0.8, 8.6)			(0, 0)	(0, 3)	(1, 1)	(1, 3)	(12, 12)	(9, 12)
MCMSeq	5	6.2	4.2	0.1	1.4	0	5.8	0.2	0.19	0	3	1	4	20	16
MCMSeq		(4.9, 7.2)	(3.5, 4.8)	(0, 0.2)	(0.8, 1.9)	(0, 0.1)	(1.9, 8.3)			(0, 0)	(1, 6)	(1, 2)	(2, 6)	(19, 20)	(14, 19)
MCMSeq	10	6.2	4.3	0.1	1.4	0	6.4	0.2	0.21	0	5	2	6	39	30
MCMSeq		(4.9, 7.2)	(3.7, 4.8)	(0, 0.2)	(1, 1.8)	(0, 0.1)	(3.5, 8.4)			(0, 0)	(3, 8)	(1, 2)	(4, 8)	(36, 40)	(27, 34)

2.3.2 Type 1 Error Rates

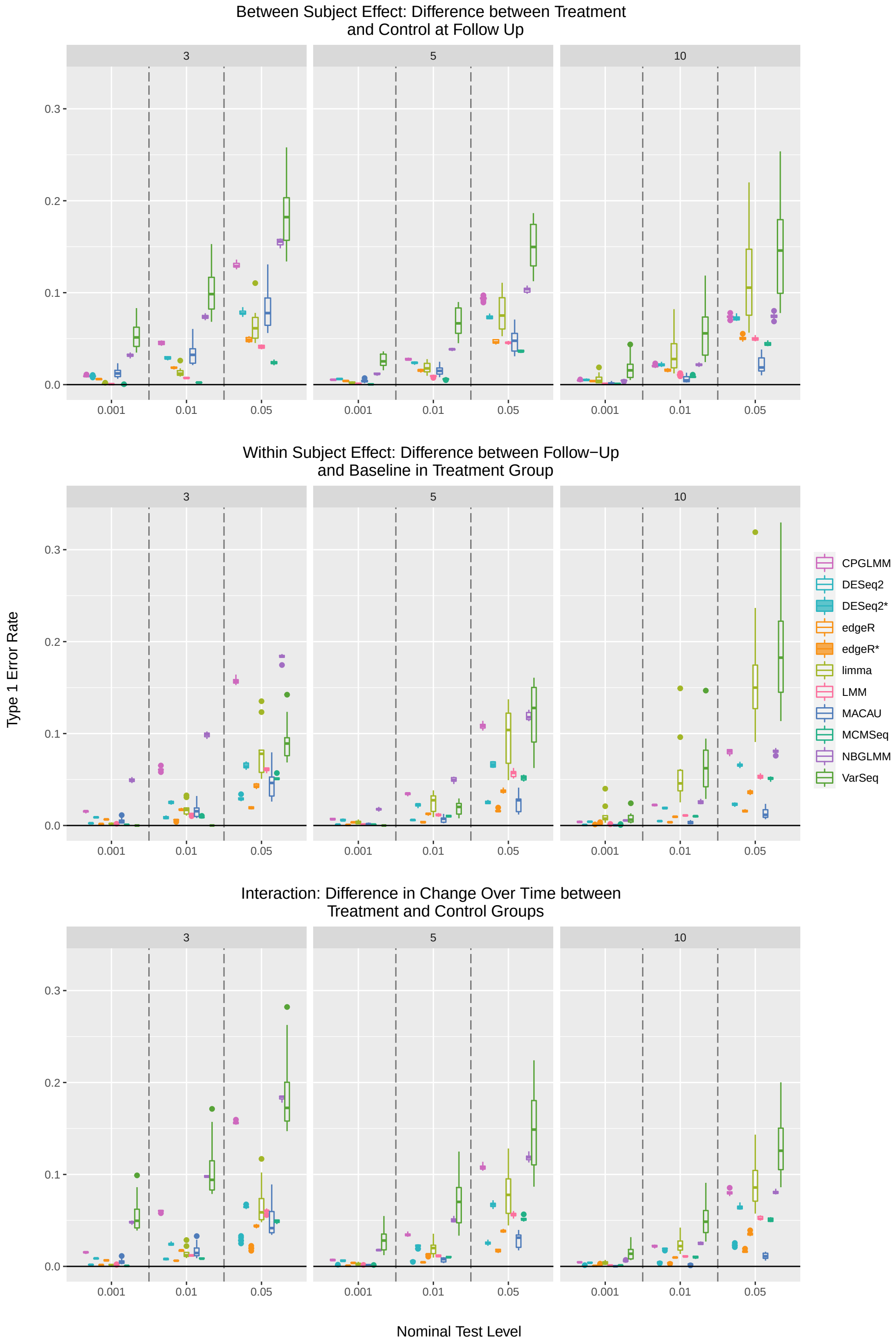

Figure 5: Simulation Study: Variability in type 1 error rates across the 10 simulated datasets

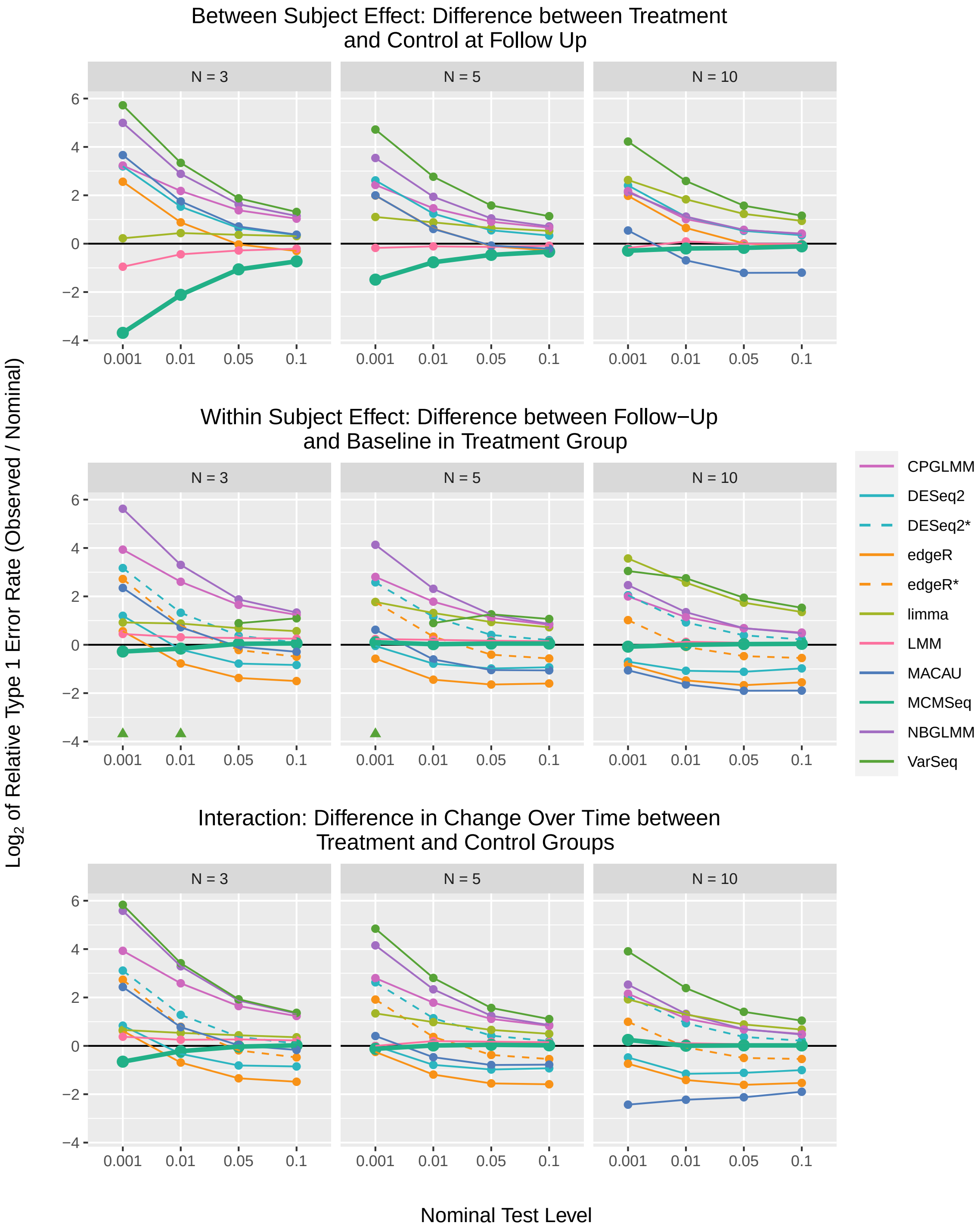

Figure 6: Simulation Study: Relative Type 1 Error Rates

Table 4: a) Simulation Study: Type 1 Error Rates by Contrast. Mean (Range) across the 10 simulated datasets.

Between Subject Effects

Difference between Treatment and Control at Baseline

	N = 3					N = 5					N = 10				
	# NA	0.001	0.01	0.05	0.1	# NA	0.001	0.01	0.05	0.1	# NA	0.001	0.01	0.05	0.1
CPGLMM	4	0.009 (0.008, 0.01)	0.044 (0.042, 0.048)	0.128 (0.125, 0.131)	0.202 (0.196, 0.211)	7	0.005 (0.005, 0.007)	0.028 (0.026, 0.033)	0.094 (0.088, 0.097)	0.159 (0.155, 0.164)	1	0.004 (0.003, 0.006)	0.019 (0.017, 0.022)	0.072 (0.069, 0.077)	0.131 (0.127, 0.141)
DESeq2	1295	0.009 (0.007, 0.01)	0.029 (0.027, 0.031)	0.079 (0.075, 0.083)	0.128 (0.123, 0.135)	979	0.006 (0.005, 0.007)	0.024 (0.021, 0.027)	0.074 (0.071, 0.079)	0.127 (0.123, 0.13)	0	0.005 (0.004, 0.006)	0.021 (0.019, 0.024)	0.07 (0.067, 0.075)	0.125 (0.121, 0.131)
edgeR	0	0.006 (0.005, 0.007)	0.019 (0.017, 0.02)	0.049 (0.046, 0.051)	0.082 (0.079, 0.085)	0	0.004 (0.003, 0.005)	0.016 (0.014, 0.018)	0.048 (0.044, 0.051)	0.084 (0.08, 0.087)	0	0.004 (0.003, 0.005)	0.015 (0.013, 0.017)	0.048 (0.045, 0.052)	0.087 (0.084, 0.092)
limma	0	0.001 (0.001, 0.001)	0.01 (0.007, 0.019)	0.051 (0.04, 0.088)	0.102 (0.08, 0.169)	0	0.001 (0.001, 0.002)	0.011 (0.007, 0.015)	0.054 (0.04, 0.071)	0.107 (0.084, 0.14)	0	0.001 (0.001, 0.002)	0.011 (0.007, 0.022)	0.052 (0.041, 0.089)	0.103 (0.088, 0.157)
LMM	32060	0.001 (0, 0.001)	0.007 (0.005, 0.008)	0.042 (0.038, 0.046)	0.086 (0.082, 0.093)	23200	0.001 (0, 0.001)	0.009 (0.008, 0.011)	0.047 (0.046, 0.049)	0.094 (0.092, 0.097)	14150	0.001 (0.001, 0.001)	0.01 (0.009, 0.011)	0.048 (0.045, 0.052)	0.097 (0.091, 0.107)
MACAU	35	0.015 (0.011, 0.031)	0.044 (0.032, 0.079)	0.102 (0.079, 0.164)	0.159 (0.128, 0.234)	4	0.007 (0.005, 0.01)	0.027 (0.02, 0.035)	0.082 (0.067, 0.103)	0.139 (0.118, 0.173)	0	0.003 (0.002, 0.007)	0.017 (0.013, 0.028)	0.065 (0.056, 0.095)	0.118 (0.108, 0.154)
MCMSeq	184	0 (0, 0)	0.002 (0.001, 0.003)	0.024 (0.021, 0.026)	0.059 (0.056, 0.063)	335	0 (0, 0.001)	0.006 (0.005, 0.007)	0.036 (0.033, 0.04)	0.078 (0.073, 0.081)	1526	0.001 (0, 0.001)	0.008 (0.007, 0.009)	0.043 (0.04, 0.046)	0.089 (0.085, 0.096)
NBGLMM	6113	0.031 (0.029, 0.034)	0.074 (0.071, 0.077)	0.152 (0.147, 0.156)	0.219 (0.212, 0.229)	9992	0.012 (0.01, 0.013)	0.038 (0.035, 0.043)	0.103 (0.098, 0.107)	0.165 (0.161, 0.168)	11995	0.004 (0.004, 0.005)	0.021 (0.018, 0.023)	0.073 (0.07, 0.078)	0.129 (0.123, 0.135)
VarSeq	0	0.046 (0.03, 0.085)	0.088 (0.059, 0.154)	0.161 (0.117, 0.256)	0.222 (0.17, 0.327)	0	0.015 (0.01, 0.02)	0.042 (0.031, 0.055)	0.106 (0.085, 0.133)	0.165 (0.141, 0.204)	0	0.005 (0.003, 0.012)	0.023 (0.018, 0.043)	0.075 (0.062, 0.116)	0.13 (0.114, 0.184)

Difference between Treatment and Control at Follow-Up

	N = 3					N = 5					N = 10				
	# NA	0.001	0.01	0.05	0.1	# NA	0.001	0.01	0.05	0.1	# NA	0.001	0.01	0.05	0.1
CPGLMM	4	0.009 (0.008, 0.011)	0.045 (0.042, 0.048)	0.13 (0.125, 0.136)	0.205 (0.2, 0.21)	7	0.005 (0.005, 0.007)	0.028 (0.026, 0.029)	0.094 (0.089, 0.097)	0.158 (0.152, 0.163)	1	0.005 (0.004, 0.006)	0.02 (0.018, 0.023)	0.074 (0.07, 0.078)	0.134 (0.127, 0.141)
DESeq2	1295	0.009 (0.008, 0.01)	0.029 (0.026, 0.031)	0.078 (0.074, 0.084)	0.129 (0.123, 0.135)	979	0.006 (0.005, 0.007)	0.024 (0.022, 0.025)	0.073 (0.071, 0.077)	0.126 (0.122, 0.132)	0	0.005 (0.004, 0.007)	0.022 (0.019, 0.025)	0.072 (0.069, 0.078)	0.127 (0.121, 0.136)
edgeR	0	0.006 (0.005, 0.007)	0.018 (0.016, 0.02)	0.049 (0.045, 0.053)	0.081 (0.078, 0.084)	0	0.004 (0.003, 0.005)	0.015 (0.013, 0.017)	0.047 (0.044, 0.049)	0.082 (0.077, 0.087)	0	0.004 (0.003, 0.005)	0.016 (0.013, 0.018)	0.05 (0.047, 0.055)	0.09 (0.087, 0.096)
limma	0	0.001 (0.001, 0.002)	0.014 (0.009, 0.026)	0.064 (0.045, 0.11)	0.124 (0.09, 0.197)	0	0.002 (0.001, 0.004)	0.018 (0.01, 0.028)	0.078 (0.053, 0.111)	0.144 (0.107, 0.187)	0	0.006 (0.001, 0.019)	0.036 (0.012, 0.082)	0.117 (0.056, 0.22)	0.192 (0.11, 0.32)
LMM	32060	0.001 (0, 0.001)	0.007 (0.006, 0.009)	0.041 (0.038, 0.044)	0.087 (0.083, 0.091)	23200	0.001 (0, 0.001)	0.009 (0.007, 0.011)	0.046 (0.043, 0.048)	0.094 (0.091, 0.098)	14150	0.001 (0, 0.001)	0.011 (0.009, 0.012)	0.05 (0.047, 0.054)	0.1 (0.095, 0.11)
MACAU	39	0.013 (0.006, 0.023)	0.034 (0.021, 0.061)	0.082 (0.056, 0.131)	0.129 (0.095, 0.195)	5	0.004 (0.002, 0.007)	0.015 (0.008, 0.025)	0.047 (0.031, 0.071)	0.087 (0.063, 0.122)	2	0.001 (0, 0.004)	0.006 (0.002, 0.013)	0.022 (0.01, 0.038)	0.044 (0.024, 0.072)
MCMSeq	184	0 (0, 0)	0.002 (0.002, 0.003)	0.024 (0.021, 0.027)	0.06 (0.057, 0.063)	335	0 (0, 0.001)	0.006 (0.005, 0.007)	0.036 (0.034, 0.038)	0.079 (0.077, 0.083)	1526	0.001 (0, 0.001)	0.009 (0.007, 0.011)	0.044 (0.042, 0.048)	0.093 (0.088, 0.099)
NBGLMM	6113	0.032 (0.028, 0.035)	0.074 (0.07, 0.078)	0.155 (0.148, 0.159)	0.221 (0.214, 0.227)	9992	0.012 (0.01, 0.013)	0.038 (0.036, 0.04)	0.103 (0.099, 0.108)	0.164 (0.159, 0.17)	11995	0.004 (0.003, 0.005)	0.022 (0.019, 0.024)	0.074 (0.069, 0.08)	0.132 (0.125, 0.137)
VarSeq	0	0.053 (0.035, 0.083)	0.101 (0.068, 0.153)	0.183 (0.134, 0.258)	0.248 (0.194, 0.334)	0	0.026 (0.016, 0.036)	0.068 (0.045, 0.09)	0.149 (0.112, 0.187)	0.22 (0.176, 0.266)	0	0.019 (0.005, 0.044)	0.06 (0.025, 0.119)	0.149 (0.078, 0.254)	0.223 (0.136, 0.343)

Table 4: b) Simulation Study: Type 1 Error Rates by Contrast. Mean (Range) across the 10 simulated datasets.

Within Subject Effects

Difference between Follow-Up and Baseline in the Control Group

	N = 3					N = 5					N = 10				
	# NA	0.001	0.01	0.05	0.1	# NA	0.001	0.01	0.05	0.1	# NA	0.001	0.01	0.05	0.1
CPGLMM	4	0.015 (0.013, 0.016)	0.06 (0.057, 0.065)	0.155 (0.152, 0.158)	0.235 (0.229, 0.237)	7	0.007 (0.005, 0.008)	0.035 (0.032, 0.036)	0.107 (0.104, 0.112)	0.175 (0.169, 0.182)	1	0.004 (0.003, 0.006)	0.022 (0.02, 0.024)	0.078 (0.077, 0.082)	0.139 (0.137, 0.141)
DESeq2	1295	0.002 (0.001, 0.002)	0.009 (0.008, 0.01)	0.029 (0.027, 0.031)	0.055 (0.052, 0.058)	979	0.001 (0.001, 0.001)	0.006 (0.005, 0.007)	0.026 (0.024, 0.028)	0.052 (0.049, 0.057)	0	0.001 (0, 0.001)	0.005 (0.003, 0.006)	0.023 (0.021, 0.025)	0.05 (0.046, 0.053)
DESeq2*	0	0.009 (0.007, 0.01)	0.025 (0.022, 0.027)	0.063 (0.06, 0.065)	0.104 (0.1, 0.106)	0	0.006 (0.005, 0.007)	0.022 (0.02, 0.024)	0.066 (0.063, 0.068)	0.113 (0.109, 0.117)	0	0.004 (0.003, 0.005)	0.019 (0.016, 0.02)	0.064 (0.061, 0.066)	0.116 (0.113, 0.119)
edgeR	0	0.001 (0.001, 0.002)	0.006 (0.004, 0.006)	0.019 (0.016, 0.021)	0.035 (0.031, 0.038)	0	0.001 (0, 0.001)	0.004 (0.003, 0.005)	0.017 (0.015, 0.019)	0.033 (0.03, 0.037)	0	0 (0, 0.001)	0.003 (0.003, 0.004)	0.016 (0.015, 0.019)	0.034 (0.031, 0.036)
edgeR*	0	0.006 (0.005, 0.008)	0.017 (0.015, 0.019)	0.043 (0.04, 0.046)	0.07 (0.066, 0.073)	0	0.004 (0.003, 0.005)	0.013 (0.011, 0.014)	0.038 (0.034, 0.041)	0.068 (0.063, 0.07)	0	0.002 (0.001, 0.002)	0.01 (0.008, 0.011)	0.036 (0.033, 0.038)	0.068 (0.066, 0.071)
limma	0	0.001 (0.001, 0.001)	0.011 (0.009, 0.012)	0.05 (0.045, 0.056)	0.099 (0.089, 0.108)	0	0.001 (0.001, 0.002)	0.012 (0.009, 0.017)	0.052 (0.047, 0.069)	0.099 (0.089, 0.13)	0	0.001 (0.001, 0.002)	0.012 (0.009, 0.015)	0.054 (0.048, 0.067)	0.102 (0.092, 0.123)
LMM	32060	0.001 (0.001, 0.002)	0.012 (0.01, 0.014)	0.059 (0.056, 0.063)	0.116 (0.111, 0.12)	23200	0.001 (0.001, 0.001)	0.011 (0.01, 0.013)	0.056 (0.053, 0.059)	0.109 (0.104, 0.113)	14150	0.001 (0.001, 0.002)	0.01 (0.01, 0.012)	0.053 (0.05, 0.057)	0.106 (0.103, 0.11)
MACAU	37	0.016 (0.011, 0.031)	0.044 (0.032, 0.079)	0.103 (0.079, 0.163)	0.159 (0.128, 0.234)	4	0.007 (0.005, 0.01)	0.027 (0.02, 0.035)	0.082 (0.067, 0.102)	0.139 (0.118, 0.173)	2	0.003 (0.002, 0.007)	0.017 (0.013, 0.028)	0.065 (0.057, 0.095)	0.119 (0.109, 0.154)
MCMSeq	184	0.001 (0, 0.001)	0.009 (0.007, 0.01)	0.049 (0.048, 0.051)	0.101 (0.097, 0.105)	335	0.001 (0.001, 0.001)	0.01 (0.009, 0.012)	0.051 (0.048, 0.055)	0.102 (0.098, 0.105)	1526	0.001 (0.001, 0.002)	0.01 (0.009, 0.011)	0.05 (0.048, 0.054)	0.101 (0.098, 0.108)
NBGLMM	6113	0.048 (0.045, 0.05)	0.097 (0.094, 0.099)	0.182 (0.179, 0.185)	0.251 (0.246, 0.255)	9992	0.017 (0.016, 0.019)	0.05 (0.046, 0.053)	0.118 (0.114, 0.122)	0.18 (0.174, 0.185)	11995	0.006 (0.005, 0.006)	0.025 (0.023, 0.027)	0.08 (0.077, 0.083)	0.137 (0.135, 0.14)
VarSeq	0	0 (0, 0)	0 (0, 0)	0.064 (0.058, 0.071)	0.157 (0.144, 0.172)	0	0 (0, 0)	0.009 (0.008, 0.012)	0.071 (0.064, 0.089)	0.138 (0.127, 0.165)	0	0.001 (0, 0.001)	0.012 (0.011, 0.015)	0.062 (0.056, 0.069)	0.12 (0.11, 0.129)

Difference between Follow-Up and Baseline in the Treatment Group

	N = 3					N = 5					N = 10				
	# NA	0.001	0.01	0.05	0.1	# NA	0.001	0.01	0.05	0.1	# NA	0.001	0.01	0.05	0.1
CPGLMM	4	0.015 (0.013, 0.017)	0.061 (0.058, 0.065)	0.157 (0.153, 0.164)	0.236 (0.228, 0.24)	7	0.007 (0.006, 0.008)	0.034 (0.032, 0.036)	0.108 (0.103, 0.114)	0.177 (0.172, 0.186)	1	0.004 (0.003, 0.005)	0.022 (0.021, 0.023)	0.08 (0.075, 0.083)	0.142 (0.136, 0.148)
DESeq2	1295	0.002 (0.001, 0.003)	0.009 (0.007, 0.011)	0.029 (0.026, 0.034)	0.056 (0.052, 0.062)	979	0.001 (0.001, 0.001)	0.006 (0.005, 0.006)	0.025 (0.023, 0.028)	0.052 (0.048, 0.056)	0	0.001 (0, 0.001)	0.005 (0.004, 0.005)	0.023 (0.02, 0.025)	0.051 (0.046, 0.054)
DESeq2*	0	0.009 (0.008, 0.01)	0.025 (0.023, 0.027)	0.065 (0.061, 0.07)	0.106 (0.102, 0.111)	0	0.006 (0.004, 0.008)	0.022 (0.019, 0.024)	0.066 (0.063, 0.07)	0.114 (0.109, 0.119)	0	0.004 (0.003, 0.005)	0.019 (0.018, 0.02)	0.066 (0.062, 0.069)	0.116 (0.113, 0.121)
edgeR	0	0.001 (0.001, 0.002)	0.006 (0.004, 0.007)	0.019 (0.017, 0.021)	0.035 (0.033, 0.039)	0	0.001 (0, 0.001)	0.004 (0.003, 0.005)	0.016 (0.014, 0.019)	0.033 (0.03, 0.036)	0	0.001 (0, 0.001)	0.004 (0.003, 0.004)	0.016 (0.014, 0.018)	0.034 (0.031, 0.036)
edgeR*	0	0.007 (0.006, 0.007)	0.017 (0.016, 0.019)	0.043 (0.04, 0.046)	0.071 (0.066, 0.076)	0	0.003 (0.003, 0.004)	0.013 (0.011, 0.014)	0.038 (0.035, 0.041)	0.068 (0.064, 0.07)	0	0.002 (0.001, 0.004)	0.009 (0.008, 0.01)	0.036 (0.033, 0.039)	0.068 (0.065, 0.074)
limma	0	0.002 (0.001, 0.004)	0.018 (0.011, 0.033)	0.08 (0.051, 0.135)	0.148 (0.103, 0.227)	0	0.003 (0.001, 0.007)	0.025 (0.009, 0.038)	0.096 (0.049, 0.137)	0.165 (0.096, 0.221)	0	0.012 (0.003, 0.04)	0.059 (0.025, 0.149)	0.167 (0.091, 0.319)	0.256 (0.16, 0.424)
LMM	32060	0.001 (0.001, 0.002)	0.012 (0.01, 0.014)	0.061 (0.057, 0.063)	0.119 (0.115, 0.124)	23200	0.001 (0, 0.002)	0.012 (0.009, 0.014)	0.056 (0.051, 0.063)	0.11 (0.104, 0.117)	14150	0.001 (0, 0.002)	0.011 (0.009, 0.012)	0.053 (0.05, 0.057)	0.105 (0.101, 0.111)
MACAU	37	0.005 (0.002, 0.011)	0.017 (0.008, 0.032)	0.047 (0.026, 0.08)	0.082 (0.05, 0.129)	5	0.002 (0.001, 0.003)	0.007 (0.002, 0.013)	0.024 (0.012, 0.041)	0.048 (0.024, 0.076)	0	0 (0, 0.001)	0.003 (0.001, 0.006)	0.013 (0.007, 0.024)	0.027 (0.014, 0.046)
MCMSeq	184	0.001 (0.001, 0.001)	0.009 (0.008, 0.01)	0.051 (0.049, 0.057)	0.104 (0.102, 0.11)	335	0.001 (0.001, 0.002)	0.01 (0.009, 0.012)	0.052 (0.048, 0.055)	0.104 (0.1, 0.109)	1526	0.001 (0.001, 0.002)	0.01 (0.009, 0.011)	0.051 (0.048, 0.053)	0.103 (0.096, 0.108)
NBGLMM	6113	0.049 (0.046, 0.053)	0.099 (0.094, 0.102)	0.183 (0.175, 0.187)	0.252 (0.247, 0.256)	9992	0.018 (0.015, 0.02)	0.05 (0.045, 0.053)	0.119 (0.113, 0.126)	0.182 (0.174, 0.191)	11995	0.006 (0.005, 0.006)	0.025 (0.023, 0.029)	0.08 (0.076, 0.084)	0.139 (0.133, 0.146)
VarSeq	0	0 (0, 0)	0 (0, 0)	0.093 (0.069, 0.142)	0.213 (0.163, 0.296)	0	0 (0, 0)	0.019 (0.008, 0.029)	0.12 (0.063, 0.161)	0.209 (0.125, 0.269)	0	0.008 (0.002, 0.024)	0.067 (0.029, 0.147)	0.193 (0.114, 0.33)	0.289 (0.186, 0.442)

Table 4: c) Simulation Study: Type 1 Error Rates by Contrast. Mean (Range) across the 10 simulated datasets.

Interaction Effect

Difference in Change Over Time between Treatment and Control Groups

	N = 3					N = 5					N = 10				
	# NA	0.001	0.01	0.05	0.1	# NA	0.001	0.01	0.05	0.1	# NA	0.001	0.01	0.05	0.1
CPGLMM	4	0.015 (0.013, 0.016)	0.06 (0.058, 0.062)	0.156 (0.154, 0.16)	0.235 (0.232, 0.239)	7	0.007 (0.006, 0.009)	0.034 (0.032, 0.038)	0.108 (0.104, 0.114)	0.178 (0.173, 0.183)	1	0.004 (0.004, 0.005)	0.022 (0.02, 0.024)	0.08 (0.076, 0.085)	0.141 (0.136, 0.147)
DESeq2	1295	0.002 (0.001, 0.002)	0.008 (0.006, 0.009)	0.028 (0.025, 0.033)	0.055 (0.052, 0.062)	979	0.001 (0.001, 0.002)	0.006 (0.005, 0.006)	0.025 (0.022, 0.029)	0.053 (0.05, 0.056)	0	0.001 (0, 0.001)	0.004 (0.003, 0.005)	0.023 (0.021, 0.025)	0.05 (0.047, 0.052)
DESeq2*	0	0.009 (0.008, 0.009)	0.024 (0.022, 0.027)	0.065 (0.062, 0.067)	0.107 (0.103, 0.11)	0	0.006 (0.005, 0.007)	0.022 (0.019, 0.024)	0.067 (0.062, 0.072)	0.114 (0.111, 0.121)	0	0.004 (0.004, 0.005)	0.019 (0.017, 0.02)	0.065 (0.062, 0.07)	0.116 (0.112, 0.119)
edgeR	0	0.002 (0.001, 0.002)	0.006 (0.005, 0.007)	0.02 (0.017, 0.022)	0.036 (0.033, 0.04)	0	0.001 (0, 0.001)	0.004 (0.004, 0.005)	0.017 (0.015, 0.019)	0.033 (0.031, 0.035)	0	0.001 (0, 0.001)	0.004 (0.003, 0.004)	0.016 (0.015, 0.019)	0.035 (0.032, 0.039)
edgeR*	0	0.007 (0.006, 0.008)	0.017 (0.016, 0.019)	0.044 (0.041, 0.046)	0.072 (0.069, 0.076)	0	0.004 (0.003, 0.004)	0.013 (0.011, 0.014)	0.039 (0.036, 0.041)	0.068 (0.063, 0.072)	0	0.002 (0.002, 0.003)	0.01 (0.009, 0.01)	0.035 (0.033, 0.039)	0.069 (0.067, 0.072)
limma	0	0.002 (0.001, 0.003)	0.014 (0.009, 0.029)	0.068 (0.048, 0.117)	0.128 (0.099, 0.204)	0	0.003 (0.001, 0.005)	0.02 (0.01, 0.036)	0.079 (0.045, 0.128)	0.141 (0.087, 0.212)	0	0.004 (0.002, 0.007)	0.025 (0.014, 0.042)	0.092 (0.057, 0.143)	0.159 (0.11, 0.232)
LMM	32060	0.001 (0.001, 0.002)	0.012 (0.011, 0.013)	0.06 (0.056, 0.063)	0.117 (0.113, 0.121)	23200	0.001 (0.001, 0.002)	0.011 (0.01, 0.013)	0.056 (0.052, 0.06)	0.111 (0.107, 0.117)	14150	0.001 (0.001, 0.002)	0.011 (0.009, 0.012)	0.052 (0.05, 0.056)	0.105 (0.101, 0.112)
MACAU	38	0.005 (0.002, 0.011)	0.017 (0.009, 0.033)	0.051 (0.034, 0.089)	0.089 (0.065, 0.145)	3	0.001 (0, 0.003)	0.007 (0.004, 0.01)	0.029 (0.017, 0.04)	0.058 (0.037, 0.077)	1	0 (0, 0)	0.002 (0.001, 0.003)	0.011 (0.006, 0.016)	0.027 (0.016, 0.036)
MCMSeq	184	0.001 (0, 0.001)	0.009 (0.008, 0.01)	0.049 (0.046, 0.051)	0.101 (0.099, 0.105)	335	0.001 (0.001, 0.002)	0.01 (0.009, 0.011)	0.051 (0.049, 0.057)	0.102 (0.096, 0.107)	1526	0.001 (0.001, 0.001)	0.01 (0.008, 0.011)	0.051 (0.048, 0.054)	0.101 (0.096, 0.104)
NBGLMM	6113	0.048 (0.045, 0.05)	0.098 (0.097, 0.099)	0.183 (0.178, 0.185)	0.252 (0.248, 0.255)	9992	0.018 (0.017, 0.019)	0.051 (0.048, 0.055)	0.118 (0.113, 0.125)	0.181 (0.178, 0.188)	11995	0.006 (0.005, 0.007)	0.025 (0.023, 0.027)	0.081 (0.078, 0.084)	0.138 (0.135, 0.144)
VarSeq	0	0.057 (0.039, 0.099)	0.107 (0.079, 0.171)	0.19 (0.147, 0.282)	0.258 (0.211, 0.359)	0	0.029 (0.012, 0.055)	0.07 (0.033, 0.125)	0.148 (0.087, 0.224)	0.215 (0.138, 0.303)	0	0.015 (0.007, 0.032)	0.052 (0.027, 0.091)	0.133 (0.086, 0.2)	0.206 (0.146, 0.29)

2.3.3 False Discovery Rates

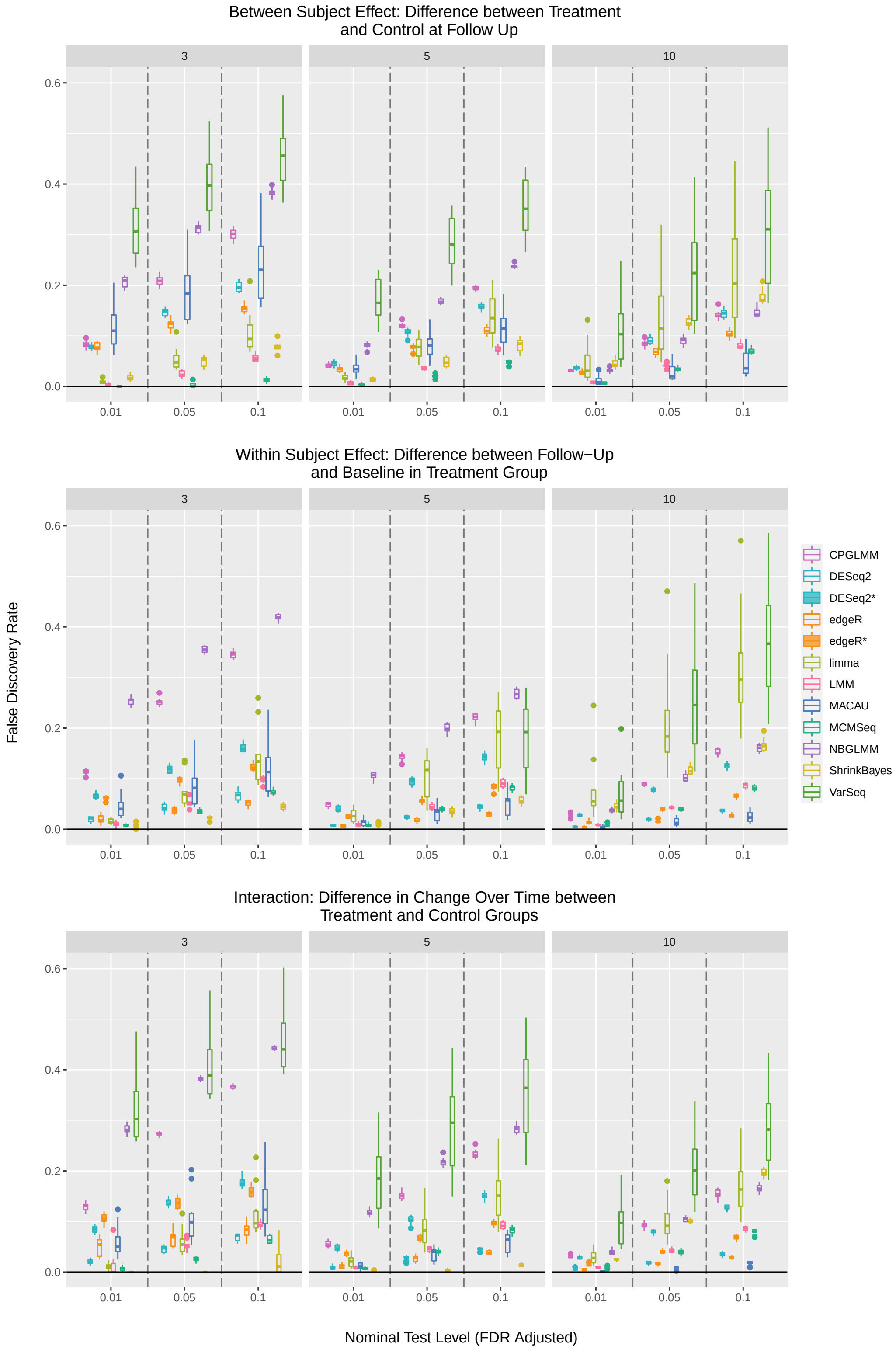

Figure 7: Simulation Study: Variability in false discovery rates across the 10 simulated datasets

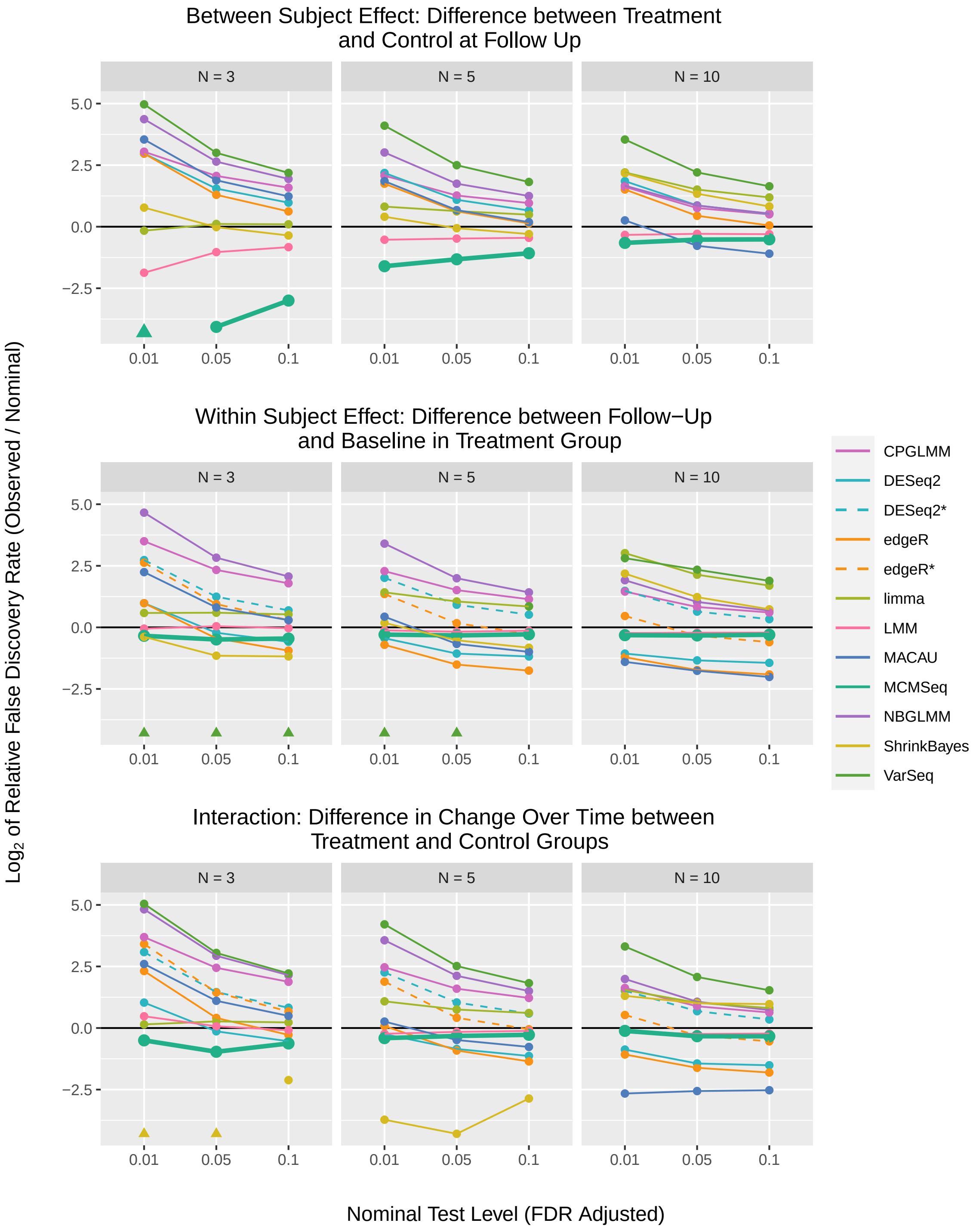

Figure 8: Simulation Study: Relative False Discovery Rates

Table 5: a) Simulation Study: False Discovery Rates by Contrast. Mean (Range) across the 10 simulated datasets.

Between Subject Effect: Difference between Treatment and Control at Follow Up

	N = 3				N = 5				N = 10			
	# NA	0.01	0.05	0.1	# NA	0.01	0.05	0.1	# NA	0.01	0.05	0.1
CPGLMM	4	0.083 (0.071, 0.096)	0.209 (0.193, 0.227)	0.3 (0.281, 0.317)	7	0.042 (0.037, 0.05)	0.12 (0.114, 0.133)	0.194 (0.187, 0.2)	1	0.031 (0.028, 0.034)	0.085 (0.073, 0.097)	0.142 (0.129, 0.163)
DESeq2	1295	0.078 (0.071, 0.087)	0.146 (0.135, 0.158)	0.197 (0.184, 0.212)	979	0.045 (0.036, 0.054)	0.107 (0.091, 0.116)	0.158 (0.146, 0.167)	0	0.036 (0.03, 0.043)	0.091 (0.082, 0.104)	0.143 (0.132, 0.159)
edgeR	0	0.078 (0.063, 0.09)	0.123 (0.103, 0.142)	0.154 (0.142, 0.17)	0	0.034 (0.026, 0.045)	0.077 (0.064, 0.086)	0.11 (0.098, 0.123)	0	0.028 (0.024, 0.036)	0.068 (0.056, 0.076)	0.104 (0.09, 0.117)
limma	0	0.009 (0.004, 0.018)	0.054 (0.033, 0.108)	0.107 (0.068, 0.208)	0	0.018 (0.006, 0.028)	0.078 (0.041, 0.112)	0.14 (0.077, 0.21)	0	0.046 (0.011, 0.131)	0.142 (0.048, 0.32)	0.228 (0.096, 0.445)
LMM	32060	0.003 (0, 0.008)	0.024 (0.015, 0.034)	0.056 (0.047, 0.07)	23200	0.007 (0.003, 0.012)	0.036 (0.031, 0.04)	0.073 (0.062, 0.085)	14150	0.008 (0.004, 0.011)	0.041 (0.033, 0.049)	0.081 (0.074, 0.094)
MACAU	39	0.116 (0.063, 0.205)	0.185 (0.123, 0.31)	0.235 (0.156, 0.382)	5	0.036 (0.015, 0.061)	0.08 (0.04, 0.133)	0.114 (0.062, 0.183)	2	0.012 (0.003, 0.033)	0.029 (0.013, 0.064)	0.047 (0.019, 0.094)
MCMSeq	184	0 (0, 0)	0.003 (0, 0.013)	0.012 (0.005, 0.021)	335	0.003 (0.001, 0.008)	0.02 (0.013, 0.026)	0.047 (0.039, 0.053)	1526	0.006 (0.003, 0.009)	0.035 (0.029, 0.042)	0.07 (0.063, 0.082)
NBGLMM	6113	0.206 (0.188, 0.22)	0.312 (0.3, 0.327)	0.384 (0.369, 0.399)	9992	0.081 (0.068, 0.089)	0.168 (0.16, 0.176)	0.237 (0.232, 0.247)	11995	0.032 (0.025, 0.04)	0.091 (0.077, 0.104)	0.145 (0.136, 0.166)
ShrinkBayes	0	0.017 (0.008, 0.028)	0.049 (0.033, 0.063)	0.078 (0.061, 0.099)	0	0.013 (0.008, 0.019)	0.048 (0.036, 0.061)	0.081 (0.059, 0.1)	0	0.045 (0.033, 0.063)	0.127 (0.111, 0.142)	0.177 (0.162, 0.208)
VarSeq	0	0.313 (0.235, 0.435)	0.4 (0.307, 0.525)	0.455 (0.363, 0.576)	0	0.172 (0.107, 0.23)	0.282 (0.199, 0.357)	0.352 (0.265, 0.434)	0	0.116 (0.038, 0.248)	0.23 (0.104, 0.414)	0.312 (0.164, 0.512)

Table 5: b) Simulation Study: False Discovery Rates by Contrast. Mean (Range) across the 10 simulated datasets.

Within Subject Effect: Difference between Follow-Up and Baseline in Treatment Group

	N = 3				N = 5				N = 10			
	# NA	0.01	0.05	0.1	# NA	0.01	0.05	0.1	# NA	0.01	0.05	0.1
CPGLMM	4	0.113 (0.102, 0.12)	0.252 (0.241, 0.269)	0.346 (0.335, 0.358)	7	0.049 (0.04, 0.054)	0.143 (0.128, 0.152)	0.221 (0.204, 0.23)	1	0.027 (0.021, 0.034)	0.089 (0.083, 0.092)	0.152 (0.142, 0.162)
DESeq2	1295	0.02 (0.01, 0.025)	0.043 (0.029, 0.054)	0.067 (0.051, 0.085)	979	0.007 (0.004, 0.01)	0.024 (0.018, 0.029)	0.044 (0.035, 0.05)	0	0.005 (0.003, 0.008)	0.02 (0.015, 0.024)	0.037 (0.031, 0.041)
DESeq2*	0	0.066 (0.058, 0.077)	0.119 (0.109, 0.132)	0.161 (0.152, 0.177)	0	0.04 (0.033, 0.049)	0.094 (0.082, 0.104)	0.143 (0.127, 0.156)	0	0.028 (0.024, 0.032)	0.078 (0.072, 0.084)	0.126 (0.115, 0.135)
edgeR	0	0.02 (0.007, 0.034)	0.037 (0.028, 0.046)	0.052 (0.04, 0.059)	0	0.006 (0.003, 0.01)	0.018 (0.011, 0.023)	0.03 (0.024, 0.036)	0	0.004 (0.002, 0.008)	0.015 (0.012, 0.021)	0.027 (0.023, 0.034)
edgeR*	0	0.062 (0.053, 0.067)	0.096 (0.084, 0.105)	0.123 (0.112, 0.137)	0	0.026 (0.02, 0.03)	0.056 (0.048, 0.065)	0.083 (0.069, 0.092)	0	0.014 (0.009, 0.023)	0.04 (0.034, 0.044)	0.066 (0.059, 0.072)
limma	0	0.015 (0.008, 0.024)	0.076 (0.043, 0.136)	0.144 (0.088, 0.26)	0	0.027 (0.009, 0.049)	0.104 (0.036, 0.16)	0.18 (0.074, 0.271)	0	0.081 (0.025, 0.245)	0.22 (0.102, 0.471)	0.324 (0.179, 0.57)
LMM	32060	0.01 (0, 0.019)	0.052 (0.039, 0.068)	0.098 (0.083, 0.107)	23200	0.009 (0.004, 0.016)	0.044 (0.035, 0.054)	0.091 (0.079, 0.102)	14150	0.009 (0.006, 0.012)	0.043 (0.039, 0.048)	0.086 (0.078, 0.093)
MACAU	37	0.047 (0.022, 0.106)	0.088 (0.044, 0.177)	0.123 (0.063, 0.236)	5	0.014 (0.004, 0.029)	0.031 (0.011, 0.062)	0.05 (0.018, 0.092)	0	0.004 (0, 0.01)	0.015 (0.005, 0.028)	0.025 (0.01, 0.044)
MCMSeq	184	0.008 (0.004, 0.011)	0.036 (0.03, 0.044)	0.073 (0.066, 0.084)	335	0.008 (0.005, 0.015)	0.04 (0.033, 0.046)	0.082 (0.072, 0.091)	1526	0.008 (0.005, 0.014)	0.04 (0.034, 0.045)	0.081 (0.074, 0.089)
NBGLMM	6113	0.253 (0.24, 0.267)	0.355 (0.345, 0.364)	0.419 (0.406, 0.428)	9992	0.106 (0.09, 0.113)	0.199 (0.182, 0.212)	0.268 (0.256, 0.282)	11995	0.038 (0.032, 0.045)	0.102 (0.094, 0.117)	0.16 (0.149, 0.171)
ShrinkBayes	0	0.008 (0, 0.015)	0.023 (0.014, 0.028)	0.044 (0.035, 0.054)	0	0.011 (0.008, 0.015)	0.035 (0.023, 0.046)	0.056 (0.044, 0.067)	0	0.045 (0.033, 0.059)	0.117 (0.108, 0.133)	0.166 (0.153, 0.195)
VarSeq	0	0 (0, 0)	0 (0, 0)	0 (0, 0)	0	0 (0, 0)	0 (0, 0)	0.181 (0.069, 0.28)	0	0.07 (0.02, 0.198)	0.253 (0.115, 0.486)	0.37 (0.208, 0.586)

Table 5: c) Simulation Study: False Discovery Rates by Contrast. Mean (Range) across the 10 simulated datasets.

Interaction: Difference in Change Over Time between Treatment and Control Groups

	N = 3				N = 5				N = 10			
	# NA	0.01	0.05	0.1	# NA	0.01	0.05	0.1	# NA	0.01	0.05	0.1
CPGLMM	4	0.129 (0.115, 0.142)	0.272 (0.265, 0.279)	0.367 (0.36, 0.373)	7	0.055 (0.046, 0.066)	0.151 (0.141, 0.167)	0.233 (0.223, 0.253)	1	0.031 (0.029, 0.036)	0.092 (0.082, 0.103)	0.155 (0.137, 0.167)
DESeq2	1295	0.02 (0.012, 0.029)	0.046 (0.036, 0.055)	0.069 (0.056, 0.076)	979	0.008 (0.004, 0.017)	0.028 (0.017, 0.034)	0.046 (0.039, 0.049)	0	0.005 (0.002, 0.01)	0.018 (0.013, 0.021)	0.035 (0.027, 0.042)
DESeq2*	0	0.085 (0.073, 0.096)	0.137 (0.126, 0.151)	0.177 (0.165, 0.2)	0	0.048 (0.039, 0.057)	0.103 (0.087, 0.113)	0.15 (0.136, 0.162)	0	0.029 (0.024, 0.035)	0.08 (0.071, 0.085)	0.127 (0.119, 0.133)
edgeR	0	0.05 (0.025, 0.076)	0.066 (0.046, 0.097)	0.082 (0.055, 0.11)	0	0.011 (0.006, 0.02)	0.027 (0.016, 0.037)	0.039 (0.034, 0.045)	0	0.005 (0.003, 0.008)	0.016 (0.011, 0.021)	0.029 (0.024, 0.034)
edgeR*	0	0.106 (0.087, 0.12)	0.136 (0.122, 0.153)	0.159 (0.147, 0.178)	0	0.037 (0.03, 0.044)	0.067 (0.058, 0.077)	0.097 (0.087, 0.105)	0	0.014 (0.012, 0.02)	0.041 (0.036, 0.047)	0.069 (0.059, 0.076)
limma	0	0.011 (0, 0.024)	0.06 (0.033, 0.116)	0.117 (0.079, 0.227)	0	0.021 (0.007, 0.043)	0.084 (0.039, 0.166)	0.153 (0.08, 0.264)	0	0.029 (0.012, 0.055)	0.102 (0.055, 0.18)	0.175 (0.099, 0.284)
LMM	32060	0.014 (0, 0.083)	0.053 (0.038, 0.072)	0.094 (0.084, 0.106)	23200	0.009 (0.006, 0.013)	0.045 (0.037, 0.052)	0.092 (0.084, 0.102)	14150	0.009 (0.005, 0.012)	0.042 (0.037, 0.051)	0.086 (0.078, 0.092)
MACAU	38	0.061 (0.025, 0.124)	0.108 (0.053, 0.202)	0.141 (0.07, 0.258)	3	0.012 (0.001, 0.021)	0.036 (0.015, 0.055)	0.059 (0.029, 0.083)	1	0.002 (0, 0.003)	0.008 (0.002, 0.012)	0.017 (0.009, 0.022)
MCMSeq	184	0.007 (0, 0.015)	0.026 (0.017, 0.031)	0.065 (0.056, 0.076)	335	0.008 (0.004, 0.012)	0.04 (0.032, 0.049)	0.083 (0.07, 0.093)	1526	0.009 (0.006, 0.013)	0.04 (0.031, 0.047)	0.079 (0.069, 0.085)
NBGLMM	6113	0.283 (0.267, 0.297)	0.382 (0.375, 0.39)	0.442 (0.437, 0.449)	9992	0.118 (0.108, 0.129)	0.217 (0.206, 0.237)	0.283 (0.271, 0.299)	11995	0.04 (0.034, 0.05)	0.105 (0.098, 0.113)	0.166 (0.153, 0.178)
ShrinkBayes	0	0 (0, 0)	0 (0, 0)	0.023 (0, 0.083)	0	0.001 (0, 0.004)	0.003 (0, 0.009)	0.014 (0.01, 0.019)	0	0.025 (0.02, 0.028)	0.101 (0.095, 0.108)	0.196 (0.183, 0.208)
VarSeq	0	0.33 (0.258, 0.476)	0.414 (0.343, 0.557)	0.465 (0.391, 0.602)	0	0.185 (0.086, 0.316)	0.286 (0.149, 0.443)	0.354 (0.211, 0.504)	0	0.099 (0.045, 0.193)	0.21 (0.119, 0.338)	0.289 (0.181, 0.433)

2.3.4 Power

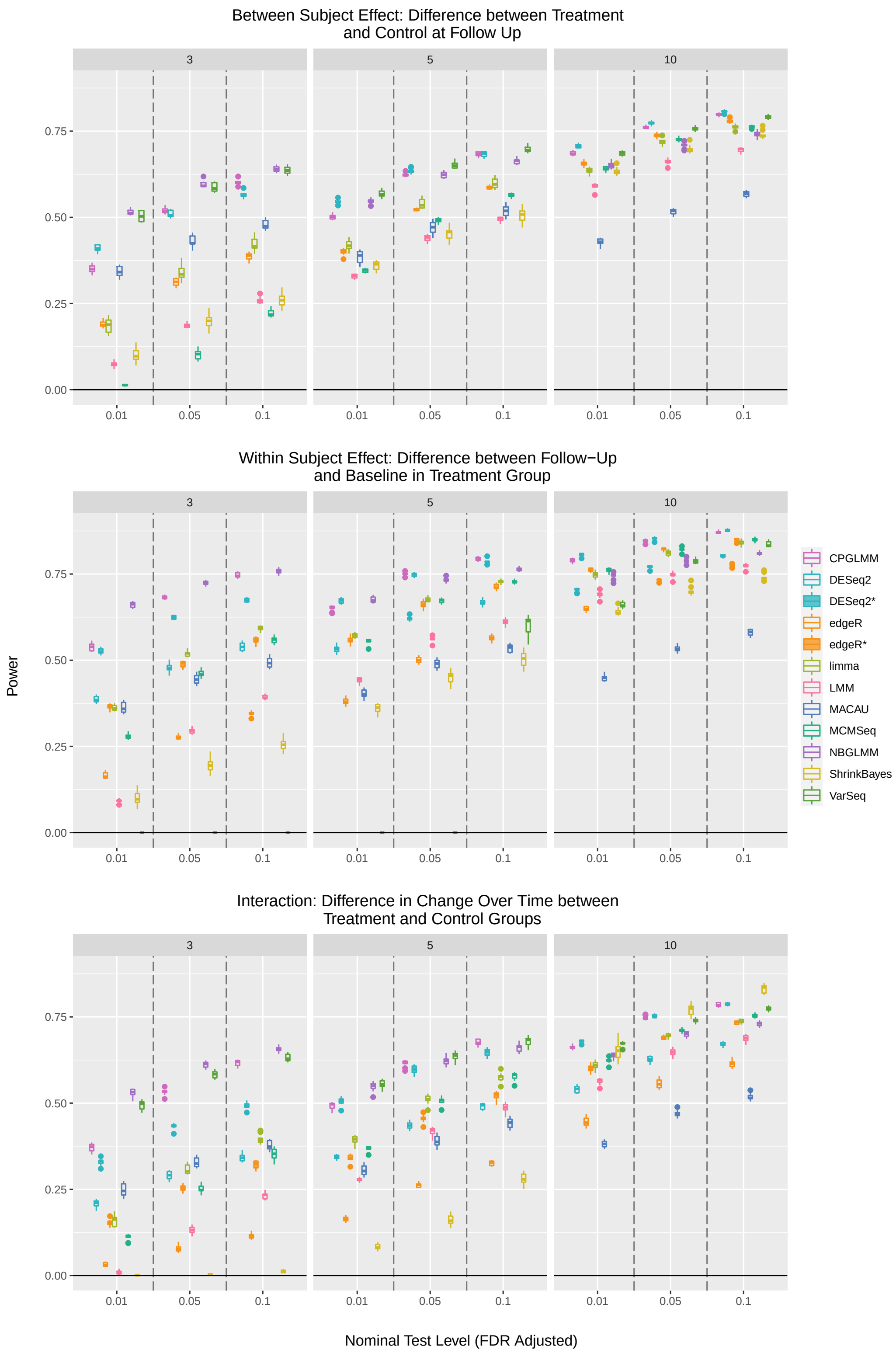

Figure 9: Simulation Study: Variability in power across the 10 simulated datasets

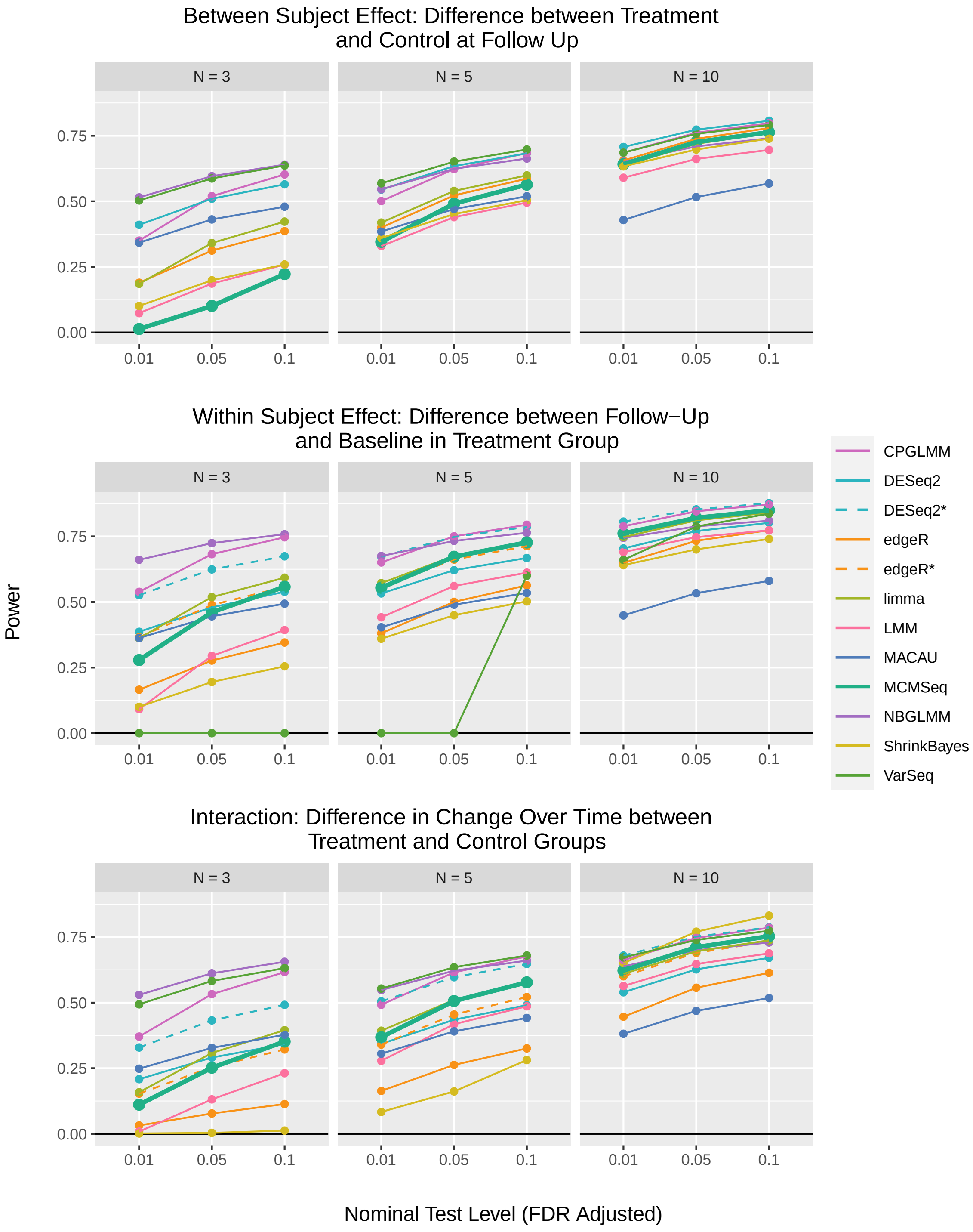

Figure 10: Simulation Study: Power in the Simulated Datasets

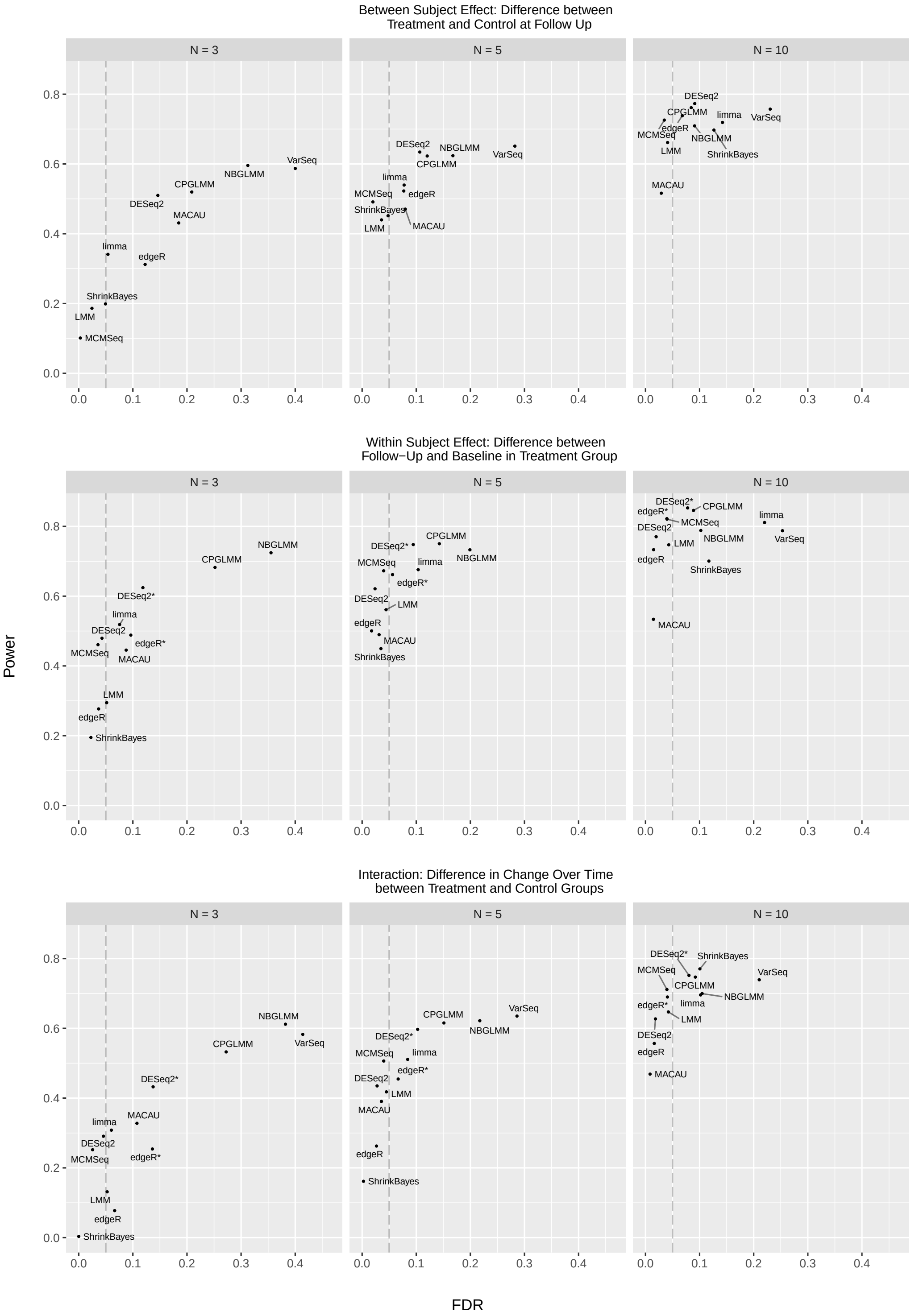

Figure 11: Simulation Study: Scatter plot of power versus observed FDR for the 3 contrasts of interest. Significance was determined at the 0.05 FDR level. Points that lie to the left of the dashed vertical line represent methods that have an observed FDR less than the nominal rate at 0.05, while points to the right represent methods with FDR inflation.

Table 6: Simulation Study: Power by Contrast. Mean (Range) across the 10 simulated datasets.

Between Subject Effect: Difference between Treatment and Control at Follow Up

	N = 3				N = 5				N = 10			
	# NA	0.01	0.05	0.1	# NA	0.01	0.05	0.1	# NA	0.01	0.05	0.1
CPGLMM	4	0.35 (0.332, 0.369)	0.52 (0.508, 0.536)	0.603 (0.589, 0.618)	7	0.501 (0.491, 0.515)	0.623 (0.616, 0.634)	0.684 (0.671, 0.691)	1	0.686 (0.677, 0.695)	0.761 (0.756, 0.769)	0.798 (0.79, 0.804)
DESeq2	1295	0.41 (0.393, 0.423)	0.51 (0.496, 0.524)	0.565 (0.552, 0.585)	979	0.545 (0.535, 0.557)	0.634 (0.624, 0.647)	0.683 (0.67, 0.692)	0	0.707 (0.7, 0.717)	0.773 (0.764, 0.782)	0.807 (0.799, 0.813)
edgeR	0	0.19 (0.178, 0.208)	0.312 (0.294, 0.325)	0.386 (0.366, 0.401)	0	0.4 (0.379, 0.411)	0.523 (0.518, 0.529)	0.586 (0.58, 0.595)	0	0.656 (0.642, 0.669)	0.738 (0.726, 0.748)	0.779 (0.772, 0.791)
limma	0	0.185 (0.155, 0.217)	0.341 (0.31, 0.383)	0.423 (0.394, 0.457)	0	0.419 (0.395, 0.442)	0.54 (0.524, 0.563)	0.599 (0.58, 0.622)	0	0.635 (0.618, 0.647)	0.719 (0.704, 0.738)	0.761 (0.748, 0.772)
LMM	32060	0.074 (0.06, 0.088)	0.186 (0.179, 0.199)	0.259 (0.249, 0.279)	23200	0.329 (0.319, 0.337)	0.44 (0.423, 0.451)	0.495 (0.48, 0.501)	14150	0.59 (0.565, 0.6)	0.661 (0.643, 0.674)	0.696 (0.682, 0.703)
MACAU	39	0.342 (0.319, 0.363)	0.431 (0.403, 0.456)	0.479 (0.461, 0.501)	5	0.385 (0.356, 0.407)	0.471 (0.441, 0.496)	0.519 (0.494, 0.544)	2	0.429 (0.408, 0.441)	0.516 (0.5, 0.527)	0.568 (0.555, 0.579)
MCMSeq	184	0.013 (0.009, 0.016)	0.101 (0.082, 0.126)	0.223 (0.209, 0.242)	335	0.345 (0.337, 0.353)	0.491 (0.478, 0.501)	0.563 (0.553, 0.572)	1526	0.642 (0.629, 0.649)	0.726 (0.717, 0.737)	0.763 (0.756, 0.769)
NBGLMM	6113	0.515 (0.505, 0.53)	0.596 (0.587, 0.618)	0.64 (0.628, 0.653)	9992	0.546 (0.533, 0.558)	0.624 (0.611, 0.637)	0.663 (0.651, 0.677)	11995	0.651 (0.639, 0.669)	0.709 (0.694, 0.722)	0.74 (0.724, 0.756)
ShrinkBayes	0	0.101 (0.07, 0.137)	0.199 (0.163, 0.238)	0.259 (0.229, 0.297)	0	0.36 (0.337, 0.376)	0.452 (0.42, 0.485)	0.504 (0.47, 0.538)	0	0.633 (0.62, 0.657)	0.697 (0.685, 0.725)	0.739 (0.727, 0.766)
VarSeq	0	0.503 (0.485, 0.522)	0.587 (0.57, 0.604)	0.636 (0.619, 0.654)	0	0.569 (0.553, 0.586)	0.651 (0.64, 0.671)	0.697 (0.686, 0.716)	0	0.685 (0.676, 0.694)	0.757 (0.747, 0.768)	0.791 (0.783, 0.8)

Table 6: Simulation Study: Power by Contrast. Mean (Range) across the 10 simulated datasets.

Within Subject Effect: Difference between Follow-Up and Baseline in Treatment Group

	N = 3				N = 5				N = 10			
	# NA	0.01	0.05	0.1	# NA	0.01	0.05	0.1	# NA	0.01	0.05	0.1
CPGLMM	4	0.539 (0.524, 0.557)	0.682 (0.674, 0.69)	0.747 (0.736, 0.759)	7	0.651 (0.636, 0.659)	0.75 (0.74, 0.759)	0.794 (0.786, 0.802)	1	0.789 (0.778, 0.798)	0.846 (0.836, 0.853)	0.871 (0.867, 0.879)
DESeq2	1295	0.386 (0.372, 0.401)	0.479 (0.455, 0.502)	0.539 (0.523, 0.557)	979	0.533 (0.515, 0.551)	0.621 (0.612, 0.634)	0.668 (0.653, 0.683)	0	0.704 (0.693, 0.71)	0.77 (0.759, 0.776)	0.802 (0.795, 0.807)
DESeq2*	0	0.526 (0.514, 0.539)	0.624 (0.617, 0.632)	0.674 (0.667, 0.683)	0	0.672 (0.661, 0.684)	0.748 (0.738, 0.756)	0.786 (0.777, 0.802)	0	0.806 (0.795, 0.812)	0.853 (0.842, 0.86)	0.877 (0.872, 0.883)
edgeR	0	0.166 (0.155, 0.181)	0.277 (0.27, 0.29)	0.345 (0.331, 0.355)	0	0.381 (0.365, 0.398)	0.5 (0.487, 0.514)	0.563 (0.549, 0.577)	0	0.649 (0.637, 0.658)	0.733 (0.724, 0.738)	0.774 (0.768, 0.78)
edgeR*	0	0.366 (0.349, 0.375)	0.488 (0.472, 0.498)	0.558 (0.538, 0.569)	0	0.558 (0.54, 0.577)	0.662 (0.642, 0.678)	0.713 (0.7, 0.725)	0	0.762 (0.753, 0.77)	0.822 (0.813, 0.827)	0.85 (0.84, 0.855)
limma	0	0.362 (0.352, 0.375)	0.519 (0.509, 0.535)	0.593 (0.579, 0.601)	0	0.572 (0.562, 0.582)	0.676 (0.668, 0.689)	0.728 (0.718, 0.737)	0	0.747 (0.733, 0.763)	0.811 (0.799, 0.822)	0.841 (0.827, 0.853)
LMM	32060	0.092 (0.08, 0.1)	0.295 (0.284, 0.309)	0.393 (0.384, 0.403)	23200	0.442 (0.426, 0.45)	0.561 (0.542, 0.572)	0.612 (0.594, 0.626)	14150	0.69 (0.67, 0.706)	0.747 (0.727, 0.76)	0.773 (0.757, 0.782)
MACAU	37	0.363 (0.343, 0.385)	0.445 (0.424, 0.467)	0.493 (0.473, 0.518)	5	0.404 (0.381, 0.421)	0.49 (0.469, 0.509)	0.535 (0.519, 0.551)	0	0.449 (0.437, 0.466)	0.534 (0.519, 0.55)	0.58 (0.564, 0.592)
MCMSeq	184	0.279 (0.268, 0.294)	0.461 (0.446, 0.48)	0.558 (0.543, 0.575)	335	0.554 (0.533, 0.562)	0.673 (0.661, 0.682)	0.727 (0.719, 0.737)	1526	0.761 (0.748, 0.769)	0.821 (0.808, 0.831)	0.85 (0.839, 0.858)
NBGLMM	6113	0.661 (0.649, 0.671)	0.724 (0.714, 0.733)	0.758 (0.745, 0.77)	9992	0.675 (0.664, 0.69)	0.733 (0.722, 0.745)	0.764 (0.756, 0.774)	11995	0.744 (0.723, 0.756)	0.788 (0.776, 0.8)	0.81 (0.803, 0.819)
ShrinkBayes	0	0.1 (0.069, 0.138)	0.195 (0.163, 0.235)	0.255 (0.227, 0.288)	0	0.36 (0.334, 0.375)	0.45 (0.416, 0.478)	0.502 (0.467, 0.537)	0	0.64 (0.629, 0.665)	0.7 (0.687, 0.731)	0.74 (0.73, 0.761)
VarSeq	0	0 (0, 0)	0 (0, 0)	0 (0, 0)	0	0 (0, 0)	0 (0, 0)	0.6 (0.545, 0.632)	0	0.661 (0.648, 0.675)	0.788 (0.778, 0.801)	0.837 (0.828, 0.851)

Table 6: Simulation Study: Power by Contrast. Mean (Range) across the 10 simulated datasets.

Interaction: Difference in Change Over Time between Treatment and Control Groups

	N = 3				N = 5				N = 10			
	# NA	0.01	0.05	0.1	# NA	0.01	0.05	0.1	# NA	0.01	0.05	0.1
CPGLMM	4	0.371 (0.351, 0.386)	0.532 (0.512, 0.548)	0.616 (0.6, 0.626)	7	0.492 (0.471, 0.503)	0.615 (0.593, 0.625)	0.676 (0.661, 0.687)	1	0.662 (0.653, 0.673)	0.747 (0.74, 0.757)	0.785 (0.777, 0.793)
DESeq2	1295	0.208 (0.187, 0.222)	0.291 (0.271, 0.306)	0.342 (0.328, 0.364)	979	0.343 (0.332, 0.352)	0.435 (0.418, 0.452)	0.49 (0.476, 0.501)	0	0.54 (0.526, 0.555)	0.627 (0.611, 0.638)	0.671 (0.659, 0.679)
DESeq2*	0	0.329 (0.309, 0.346)	0.432 (0.411, 0.442)	0.492 (0.473, 0.508)	0	0.505 (0.478, 0.52)	0.597 (0.577, 0.612)	0.647 (0.628, 0.662)	0	0.679 (0.669, 0.684)	0.752 (0.745, 0.76)	0.787 (0.78, 0.794)
edgeR	0	0.032 (0.025, 0.041)	0.077 (0.064, 0.098)	0.113 (0.102, 0.13)	0	0.164 (0.153, 0.175)	0.263 (0.255, 0.274)	0.326 (0.316, 0.334)	0	0.446 (0.426, 0.468)	0.557 (0.538, 0.578)	0.614 (0.598, 0.634)
edgeR*	0	0.153 (0.139, 0.172)	0.254 (0.237, 0.268)	0.322 (0.302, 0.335)	0	0.34 (0.315, 0.354)	0.455 (0.431, 0.473)	0.521 (0.495, 0.535)	0	0.601 (0.582, 0.619)	0.69 (0.683, 0.699)	0.734 (0.728, 0.739)
limma	0	0.158 (0.139, 0.186)	0.308 (0.293, 0.33)	0.395 (0.379, 0.42)	0	0.393 (0.366, 0.407)	0.511 (0.479, 0.528)	0.575 (0.548, 0.599)	0	0.61 (0.587, 0.626)	0.696 (0.684, 0.704)	0.738 (0.728, 0.744)
LMM	32060	0.008 (0, 0.02)	0.131 (0.113, 0.148)	0.231 (0.218, 0.249)	23200	0.278 (0.269, 0.286)	0.418 (0.391, 0.431)	0.486 (0.459, 0.504)	14150	0.563 (0.542, 0.573)	0.647 (0.628, 0.663)	0.688 (0.669, 0.701)
MACAU	38	0.248 (0.222, 0.274)	0.328 (0.31, 0.35)	0.377 (0.357, 0.396)	3	0.305 (0.284, 0.329)	0.39 (0.364, 0.415)	0.442 (0.42, 0.463)	1	0.381 (0.367, 0.395)	0.469 (0.455, 0.488)	0.518 (0.505, 0.537)
MCMSeq	184	0.111 (0.094, 0.121)	0.252 (0.232, 0.272)	0.352 (0.322, 0.374)	335	0.368 (0.35, 0.375)	0.506 (0.48, 0.522)	0.577 (0.55, 0.59)	1526	0.623 (0.604, 0.636)	0.711 (0.701, 0.72)	0.753 (0.745, 0.763)
NBGLMM	6113	0.53 (0.505, 0.542)	0.612 (0.597, 0.624)	0.656 (0.643, 0.67)	9992	0.548 (0.517, 0.564)	0.622 (0.604, 0.645)	0.66 (0.642, 0.681)	11995	0.638 (0.621, 0.646)	0.7 (0.686, 0.709)	0.729 (0.718, 0.74)
ShrinkBayes	0	0.001 (0, 0.003)	0.003 (0.001, 0.007)	0.012 (0.007, 0.018)	0	0.083 (0.07, 0.096)	0.162 (0.138, 0.186)	0.281 (0.251, 0.304)	0	0.651 (0.612, 0.704)	0.77 (0.744, 0.796)	0.831 (0.814, 0.849)
VarSeq	0	0.494 (0.472, 0.511)	0.583 (0.568, 0.599)	0.632 (0.618, 0.649)	0	0.554 (0.533, 0.571)	0.635 (0.61, 0.653)	0.68 (0.654, 0.698)	0	0.673 (0.655, 0.681)	0.739 (0.728, 0.749)	0.774 (0.763, 0.784)

2.3.5 Influence of Inverse Gamma Prior on Testing Characteristics

We evaluate the influence of the choice of U and V in the inverse Gamma prior for the random effect variance on testing characteristics of the MCMSeq method in our N=5 simulated datasets. Smaller values of U and V result in a more diffuse, less informative prior. Common choices are $U = V = 0.01$ and $U = V = 0.001$. We also evaluate $U = V = 0.1$. The $U = V = 0.1$ has slightly inflated type 1 error rates for within subject

and interaction tests, but is overly conservative for between subject tests; $U = V = 0.01$ approximately achieves nominal type 1 error rates for within subject and interaction tests, while $U = V = 0.001$ is slightly conservative. $U = V = 0.01$ and $U = V = 0.001$ are both conservative for between subject tests. All $U = V = 0.1$, $U = V = 0.01$, and $U = V = 0.001$ had FDRs below the nominal level for all tests. Power was similar for all 3 settings for within subject and interaction tests, but was reduced for $U = V = 0.1$ for the between subject tests.

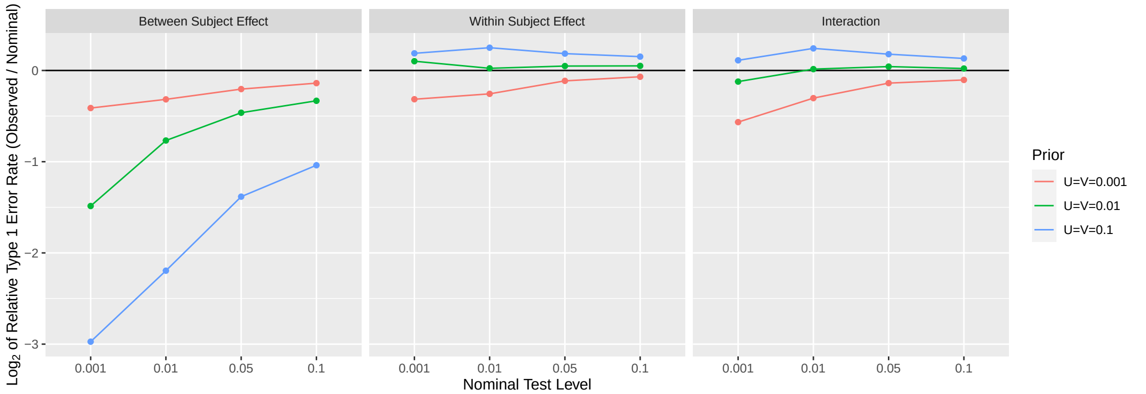

Figure 12: Simulation Study: Relative Type 1 Error Rates for Various Random Effect Variance Priors

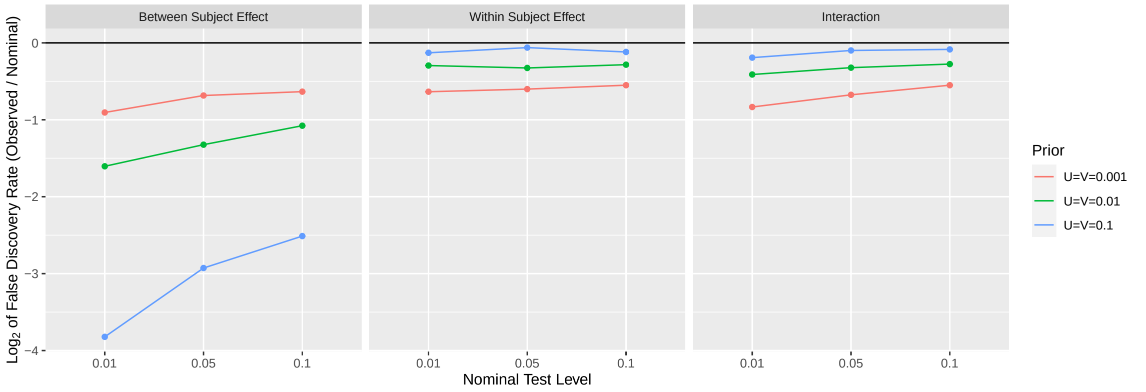

Figure 13: Simulation Study: False Discovery Rates for Various Random Effect Variance Priors

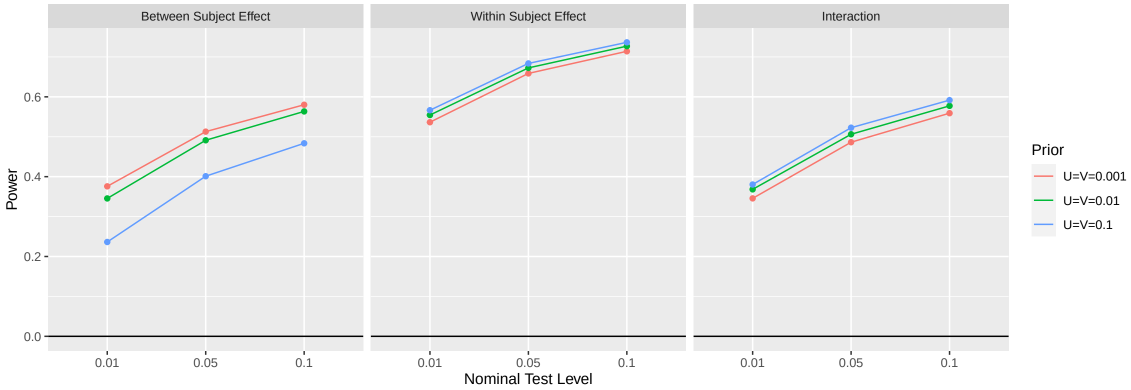

Figure 14: Simulation Study: Power for Various Random Effect Variance Priors

2.3.6 Influence of Dispersion Prior on Testing Characteristics

We evaluate the influence of the choice of k in the prior for the dispersion parameter $(\log(\alpha_g) \sim N(A_g, (k\tau^2))$ on testing characteristics of the MCMSeq method in our Null + Mixed Effect Size simulated datasets. We also compare to a constant dispersion prior for all genes, $N(7, 7^2)$. As A_g are larger than the the true mean dispersions since the method-of-moments dispersion estimates do not take into account fixed or random effects that explain variability in the data, tests become more conservative as k decreases. While $k = 1$ tends to be overly conservative, using large k or an untrended prior for the dispersion with large variance tends to have slightly inflated type 1 error and false discovery rates. We use $k = 2$ in analyses presented in this paper, which typically maintains type 1 error rates without being overly conservative. As sample size increases, results for all k become more similar.

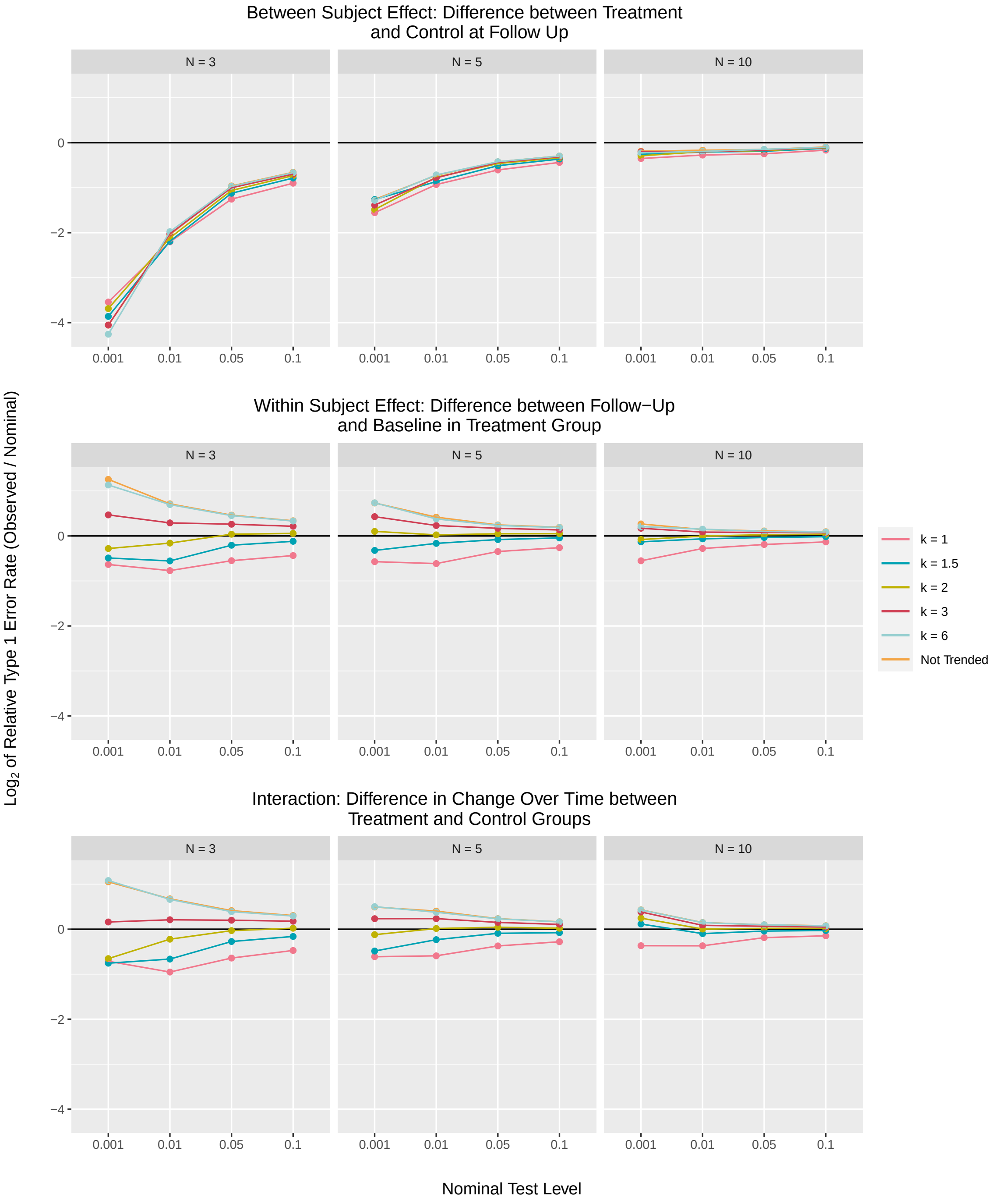

Figure 15: Simulation Study: Relative Type 1 Error Rates for Various Dispersion Priors

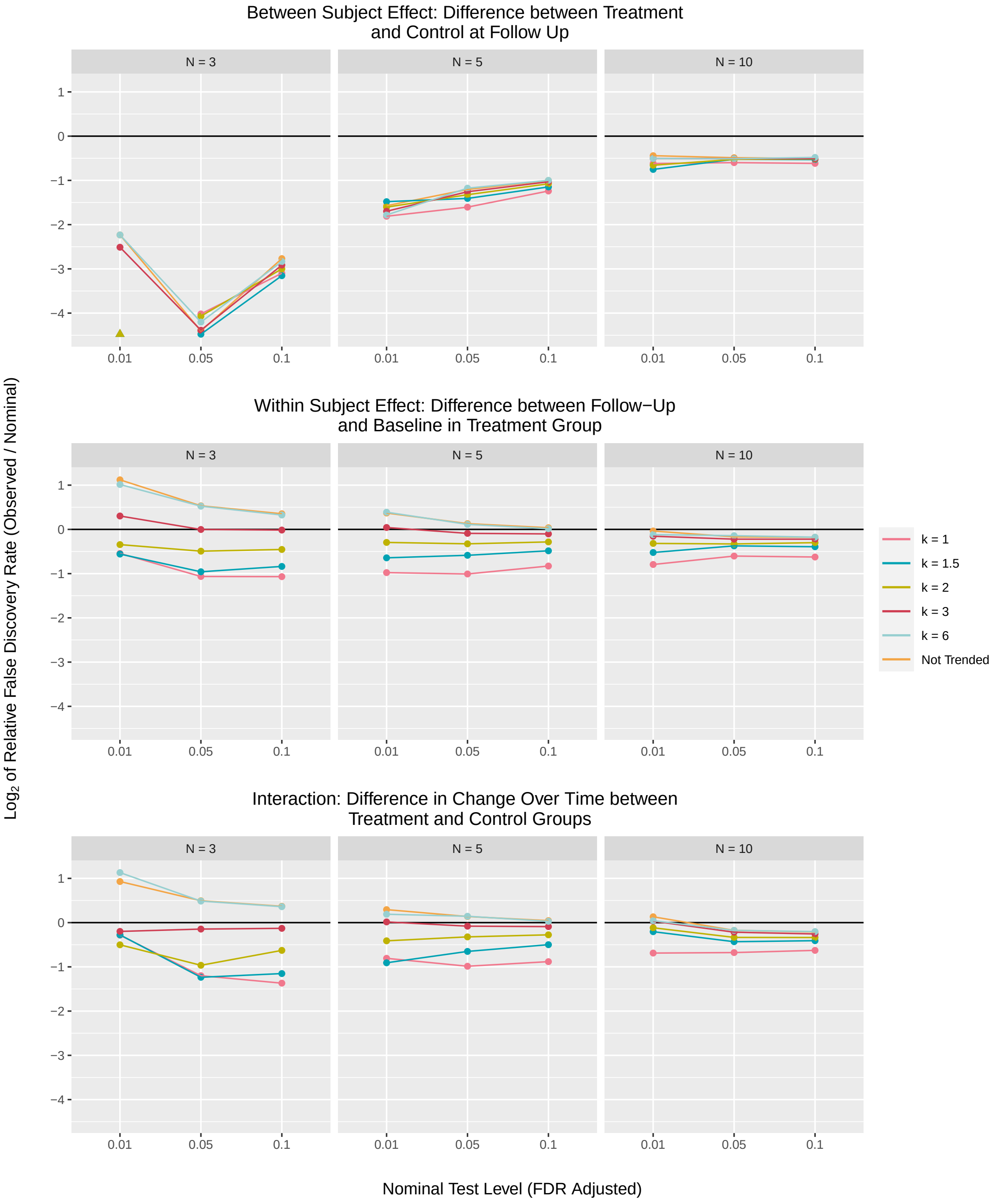

Figure 16: Simulation Study: False Discovery Rates for Various Dispersion Priors

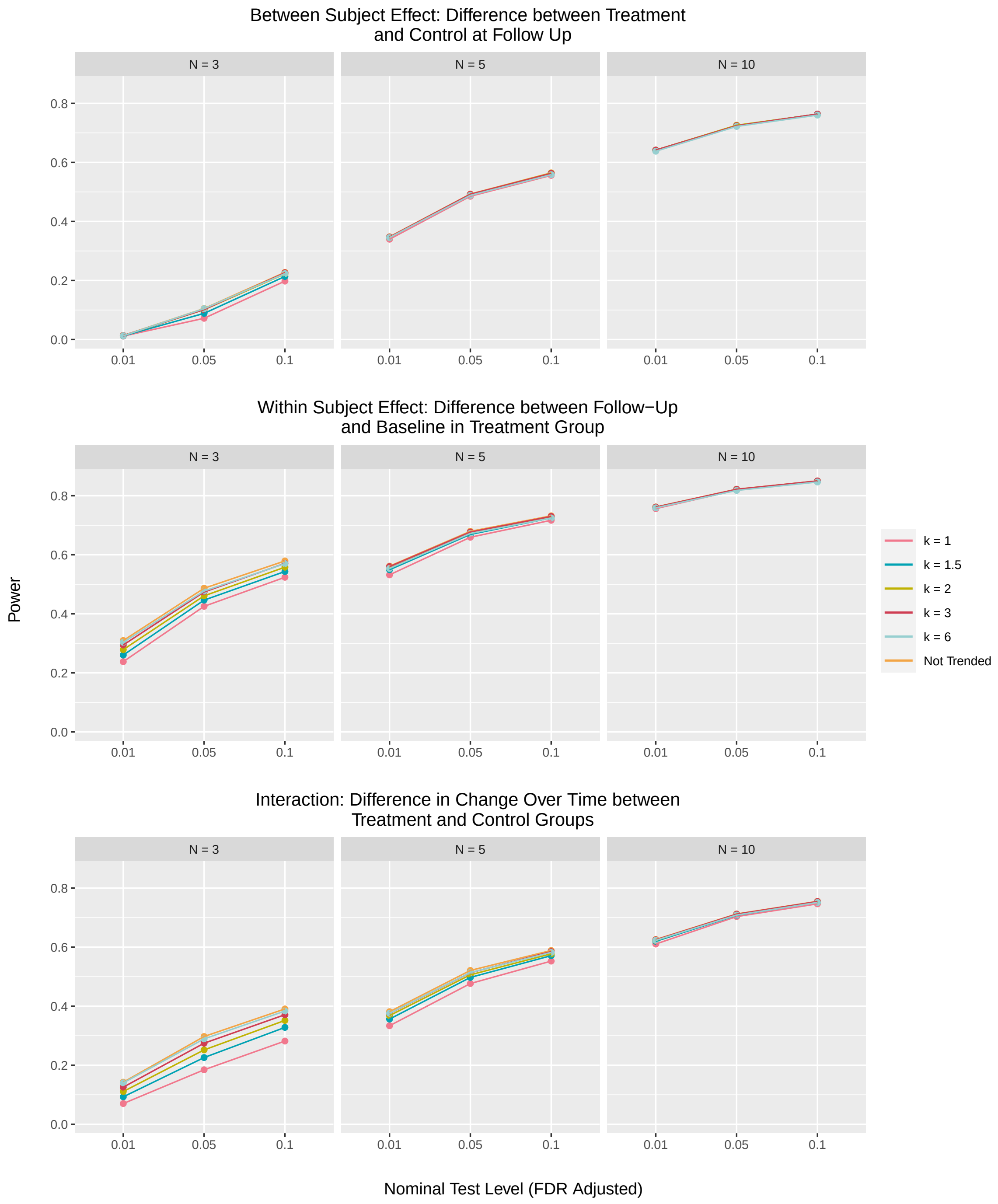

Figure 17: Simulation Study: Power for Various Dispersion Priors

2.3.7 Influence of Filtering on Testing Characteristics

To test the sensitivity of the results of our simulation study to filtering criteria, we recomputed type 1 erorr rates, false discovery rates and power using two more restrictive filtering criteria for the N=5 datasets. Our original simulation study removed features that did not have at least 1 count per million in 5 (25%) of the samples. We evaluated the following additional filtering criteria, which we call "more restrictive" and "most restrictive". For the "more restrictive" filtering, we removed features that did not have at least 1 count per million in 10 (50%) of the samples. For the "most restrictive" filtering, we removed features that did not have at least 5 counts per million in 5 samples. This resulted in approximately 5% and 20% reductions in the number of features included in the simulation compared to the original filtering. Results are presented in Figures 18-20. We see that results are consistent across the filtering scenarios. As expected, power increases as fewer low count features are included for all methods.

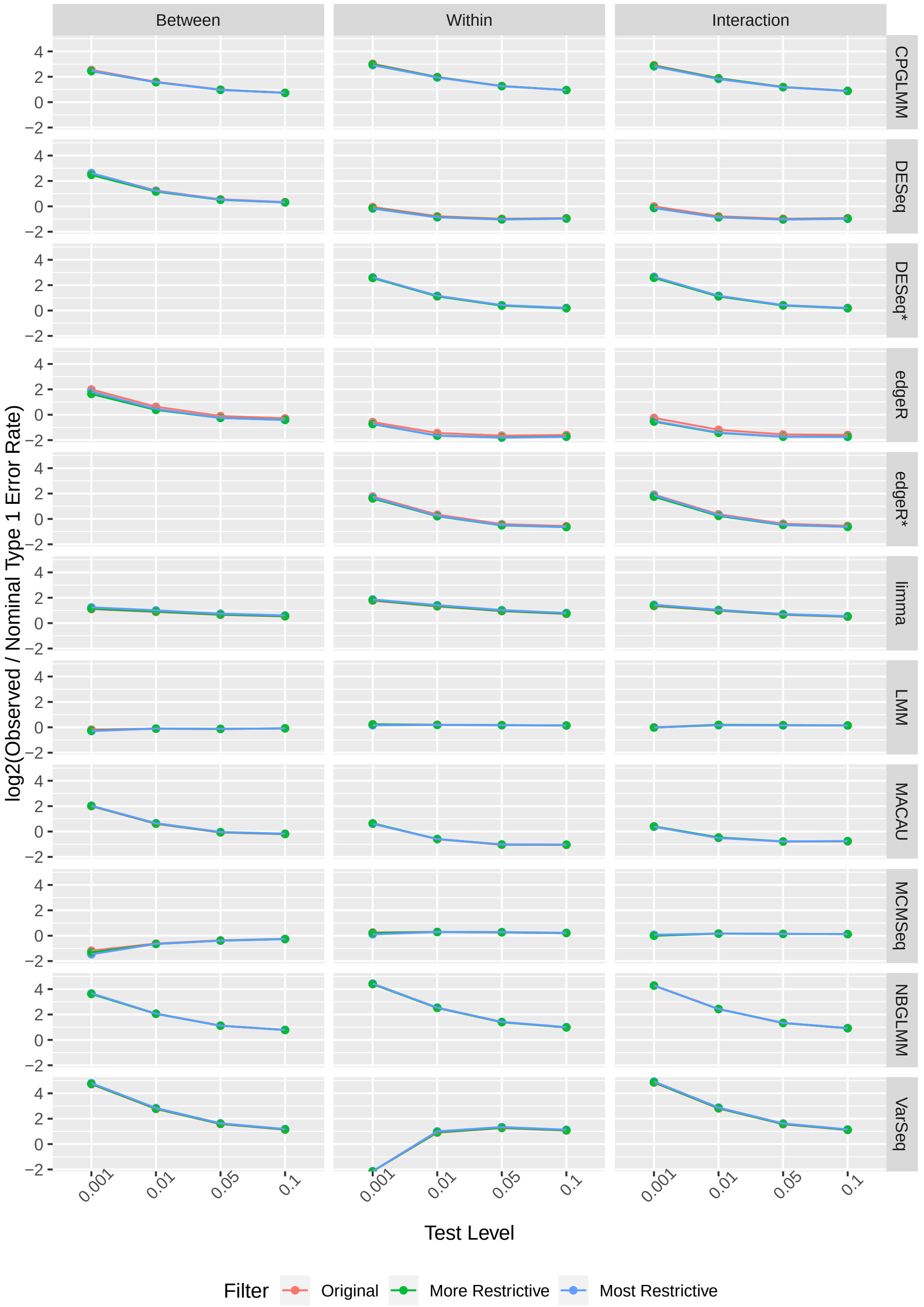

Figure 18: Simulation Study: Comparison of Relative Type 1 Error Rates Using Various Filtering Criteria

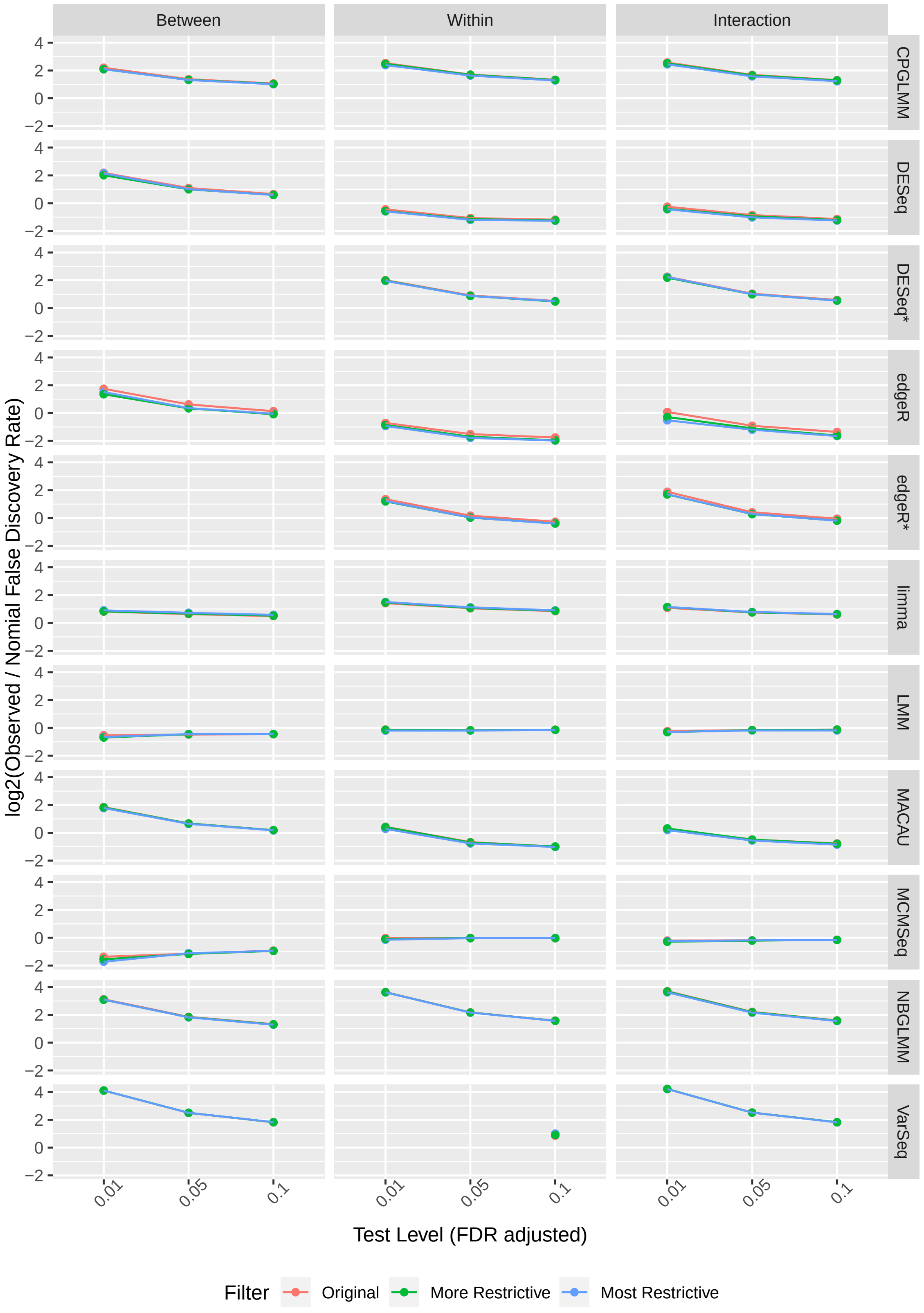

Figure 19: Simulation Study: Comparison of Relative False Discovery Rates Using Various Filtering Criteria

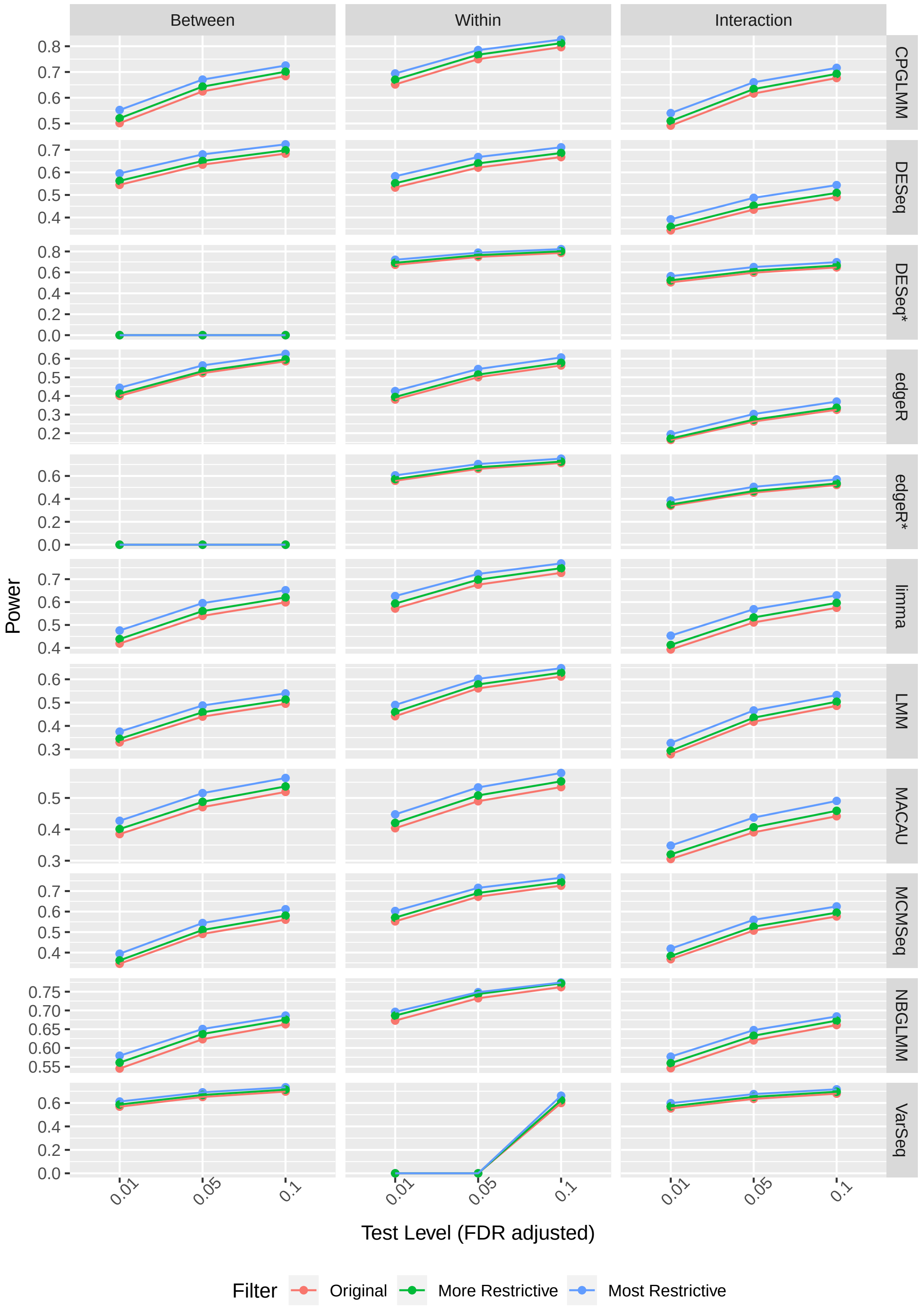

Figure 20: Simulation Study: Comparison of Power Using Various Filtering Criteria

2.3.8 MCMSeq Run Times and Computing Resources

Table 7 shows the MCMSeq run times for a single simulated dataset of each sample size. These were run using 6 threads on a MacPro desktop with a 3.5 GHz 6-core Intel Xenon E5 processor. Because of the way the parallelization and summarization of the chain for each gene is implemented in the `mcmseq` R package, MCMSeq fits do not require large amounts of RAM. Indeed, fitting the TB data, which has 112 samples and 15,993 genes only required approximately 300 MB of RAM.

Sample Size	Time (minutes)
3	36.86
5	68.62
10	258.70

Table 7: Run times for MCMSeq on the simulated datasets

3 Real Data Analysis

3.1 Full Results

Table 8: Tuberculosis Dataset: Number of Significant Genes by Contrast. 1. In order to test all contrasts of interest, ShrinkBayes models were fit three times, using a different reference group each time. This resulted in some contrasts being evaluated twice. As ShrinkBayes is a stochastic method, the number of significant genes can differ between model fits. We present the results from both models when tests were performed multiple times.

	CPGLMM	DESeq2	DESeq2*	edgeR	edgeR*	limma	LMM	MACAU	MCMSeq	NBGLMM	ShrinkBayes[1]	VarSeq
Number Failed to Converge	250	12	0	0	0	0	392	1	132	1348	0	0
**Baseline Differences**												
Latent vs. Control	3	327	-	279	-	11	2	1	3	9	416, 407	0
Progressor vs. Control	1199	2503	-	2221	-	1009	752	45	650	1130	1821, 1013	417
Progressor vs. Latent	549	1842	-	1643	-	552	299	42	256	667	1369, 708	3
**Changes Over Time**												
Control	12	1	0	1	0	0	0	0	0	0	2977	0
Latent	8	1	0	2	0	0	0	0	3	2	3171	0
Progressor	2031	1003	1084	664	790	2460	1413	0	1463	1878	7848	0
**Differences in Changes Over Time**												
Latent vs. Control	7	0	0	0	0	0	0	0	3	0	3331, 3230	0
Progressor vs. Control	1068	94	570	16	272	310	537	0	635	794	7199, 6429	0
Progressor vs. Latent	1189	331	556	170	278	1852	825	0	852	1328	6681, 7390	0

3.2 Comparison of Significant Genes Between Methods

Table 9: Tuberculosis Dataset: Agreement in differential expression calls for select contrasts. Pairwise Jaccard indices and Kappa agreement statistics are presented as Jaccard, Kappa.

Baseline: Progressor vs. Latent

	CPGLMM	DESeq2	DESeq2*	edgeR	edgeR*	limma	LMM	MACAU	MCMSeq	NBGLMM	ShrinkBayes	VarSeq
CPGLMM	-	0.02, -0.01	-	0.31, 0.45	-	0.44, 0.60	0.54, 0.69	0.08, 0.14	0.46, 0.62	0.67, 0.80	0.19, 0.28	0.01, 0.01
DESeq2	0.02, -0.01	-	-	0.04, 0	-	0.03, 0.02	0.01, -0.01	0, -0.01	0.01, -0.01	0.03, 0	0.04, 0.02	0, 0
DESeq2*	-	-	-	-	-	-	-	-	-	-	-	-
edgeR	0.31, 0.45	0.04, 0	-	-	-	0.32, 0.46	0.18, 0.28	0.03, 0.04	0.15, 0.24	0.44, 0.59	0.23, 0.32	0, 0
edgeR*	-	-	-	-	-	-	-	-	-	-	-	-
limma	0.44, 0.60	0.03, 0.02	-	0.32, 0.46	-	-	0.38, 0.54	0.07, 0.13	0.34, 0.50	0.49, 0.64	0.11, 0.16	0.01, 0.01
LMM	0.54, 0.69	0.01, -0.01	-	0.18, 0.28	-	0.38, 0.54	-	0.12, 0.22	0.73, 0.84	0.40, 0.56	0.12, 0.19	0.01, 0.02
MACAU	0.08, 0.14	0, -0.01	-	0.03, 0.04	-	0.07, 0.13	0.12, 0.22	-	0.12, 0.21	0.06, 0.10	0.02, 0.03	0.05, 0.09
MCMSeq	0.46, 0.62	0.01, -0.01	-	0.15, 0.24	-	0.34, 0.50	0.73, 0.84	0.12, 0.21	-	0.34, 0.49	0.10, 0.15	0.01, 0.03
NBGLMM	0.67, 0.80	0.03, 0	-	0.44, 0.59	-	0.49, 0.64	0.40, 0.56	0.06, 0.10	0.34, 0.49	-	0.23, 0.33	0, 0.01
ShrinkBayes	0.19, 0.28	0.04, 0.02	-	0.23, 0.32	-	0.11, 0.16	0.12, 0.19	0.02, 0.03	0.10, 0.15	0.23, 0.33	-	0, 0
VarSeq	0.01, 0.01	0, 0	-	0, 0	-	0.01, 0.01	0.01, 0.02	0.05, 0.09	0.01, 0.03	0, 0.01	0, 0	-

Change Over Time: Progressor

	CPGLMM	DESeq2	DESeq2*	edgeR	edgeR*	limma	LMM	MACAU	MCMSeq	NBGLMM	ShrinkBayes	VarSeq
CPGLMM	-	0.39, 0.53	0.47, 0.61	0.30, 0.42	0.37, 0.50	0.24, 0.29	0.66, 0.77	0, 0	0.70, 0.81	0.69, 0.79	0.23, 0.21	0, 0
DESeq2	0.39, 0.53	-	0.26, 0.38	0.64, 0.77	0.24, 0.35	0.19, 0.26	0.44, 0.58	0, 0	0.44, 0.59	0.39, 0.53	0.12, 0.11	0, 0
DESeq2*	0.47, 0.61	0.26, 0.38	-	0.27, 0.39	0.72, 0.83	0.17, 0.21	0.58, 0.71	0, 0	0.58, 0.71	0.44, 0.57	0.13, 0.12	0, 0
edgeR	0.30, 0.42	0.64, 0.77	0.27, 0.39	-	0.27, 0.4	0.16, 0.23	0.37, 0.52	0, 0	0.38, 0.52	0.29, 0.41	0.08, 0.07	0, 0
edgeR*	0.37, 0.50	0.24, 0.35	0.72, 0.83	0.27, 0.4	-	0.17, 0.23	0.49, 0.64	0, 0	0.48, 0.63	0.35, 0.49	0.09, 0.09	0, 0
limma	0.24, 0.29	0.19, 0.26	0.17, 0.21	0.16, 0.23	0.17, 0.23	-	0.21, 0.27	0, 0	0.21, 0.27	0.35, 0.44	0.27, 0.25	0, 0
LMM	0.66, 0.77	0.44, 0.58	0.58, 0.71	0.37, 0.52	0.49, 0.64	0.21, 0.27	-	0, 0	0.83, 0.9	0.61, 0.73	0.16, 0.15	0, 0
MACAU	0, 0	0, 0	0, 0	0, 0	0, 0	0, 0	0, 0	-	0, 0	0, 0	0, 0	NaN, NaN
MCMSeq	0.70, 0.81	0.44, 0.59	0.58, 0.71	0.38, 0.52	0.48, 0.63	0.21, 0.27	0.83, 0.9	0, 0	-	0.65, 0.76	0.17, 0.16	0, 0
NBGLMM	0.69, 0.79	0.39, 0.53	0.44, 0.57	0.29, 0.41	0.35, 0.49	0.35, 0.44	0.61, 0.73	0, 0	0.65, 0.76	-	0.25, 0.24	0, 0
ShrinkBayes	0.23, 0.21	0.12, 0.11	0.13, 0.12	0.08, 0.07	0.09, 0.09	0.27, 0.25	0.16, 0.15	0, 0	0.17, 0.16	0.25, 0.24	-	0, 0
VarSeq	0, 0	0, 0	0, 0	0, 0	0, 0	0, 0	0, 0	NaN, NaN	0, 0	0, 0	0, 0	-

Differences in Changes Over Time: Progressor vs. Latent

	CPGLMM	DESeq2	DESeq2*	edgeR	edgeR*	limma	LMM	MACAU	MCMSeq	NBGLMM	ShrinkBayes	VarSeq
CPGLMM	-	0.25, 0.39	0.38, 0.53	0.14, 0.23	0.22, 0.35	0.17, 0.22	0.60, 0.74	0, 0	0.65, 0.78	0.62, 0.74	0.14, 0.14	0, 0
DESeq2	0.25, 0.39	-	0.24, 0.37	0.50, 0.66	0.22, 0.35	0.10, 0.16	0.33, 0.49	0, 0	0.33, 0.48	0.20, 0.31	0.04, 0.04	0, 0
DESeq2*	0.38, 0.53	0.24, 0.37	-	0.18, 0.29	0.51, 0.67	0.10, 0.13	0.49, 0.64	0, 0	0.49, 0.64	0.32, 0.45	0.07, 0.07	0, 0
edgeR	0.14, 0.23	0.50, 0.66	0.18, 0.29	-	0.21, 0.34	0.06, 0.10	0.20, 0.32	0, 0	0.19, 0.31	0.11, 0.18	0.02, 0.02	0, 0
edgeR*	0.22, 0.35	0.22, 0.35	0.51, 0.67	0.21, 0.34	-	0.07, 0.11	0.32, 0.47	0, 0	0.32, 0.47	0.18, 0.28	0.03, 0.04	0, 0
limma	0.17, 0.22	0.10, 0.16	0.10, 0.13	0.06, 0.10	0.07, 0.11	-	0.15, 0.21	0, 0	0.15, 0.21	0.31, 0.42	0.22, 0.22	0, 0
LMM	0.60, 0.74	0.33, 0.49	0.49, 0.64	0.20, 0.32	0.32, 0.47	0.15, 0.21	-	0, 0	0.8, 0.88	0.52, 0.66	0.10, 0.10	0, 0
MACAU	0, 0	0, 0	0, 0	0, 0	0, 0	0, 0	0, 0	-	0, 0	0, 0	0, 0	NaN, NaN
MCMSeq	0.65, 0.78	0.33, 0.48	0.49, 0.64	0.19, 0.31	0.32, 0.47	0.15, 0.21	0.8, 0.88	0, 0	-	0.54, 0.68	0.10, 0.10	0, 0
NBGLMM	0.62, 0.74	0.20, 0.31	0.32, 0.45	0.11, 0.18	0.18, 0.28	0.31, 0.42	0.52, 0.66	0, 0	0.54, 0.68	-	0.19, 0.19	0, 0
ShrinkBayes	0.14, 0.14	0.04, 0.04	0.07, 0.07	0.02, 0.02	0.03, 0.04	0.22, 0.22	0.10, 0.10	0, 0	0.10, 0.10	0.19, 0.19	-	0, 0
VarSeq	0, 0	0, 0	0, 0	0, 0	0, 0	0, 0	0, 0	NaN, NaN	0, 0	0, 0	0, 0	-

3.3 Agreement Between Repeated MCMSeq Analyses

Since Markov-chain Monte Carlo is a stochastic model fitting method, MCMSeq p-values and lists of differentially expressed genes may vary slightly between repeated MCMSeq analyses on the same dataset. To investigate agreement between multiple MCMSeq runs, we fit MCMSeq models to the TB dataset 4 times and compared results in terms of the number of differntially expressed genes by contrast, Spearman correlation of p-values and Kappa-Fleiss agreement statistics. In Table 10 we see that MCMSeq p-values are highly correlated across all 4 runs. Similar numbers of significant and non-significant genes were found across the 4 runs, typically with over 99% agreement among calls across the 4 runs. Kappa-Fleiss scores for contrasts with appreciable numbers of differentially expressed genes are over 0.85, which indicates near perfect agreement (Table 11).

Table 10: Tuberculosis Dataset: Spearman correlation of p-values between 4 MCMSeq runs on the TB dataset.

	1 vs. 2	1 vs. 3	1 vs. 4	2 vs. 3	2 vs. 4	3 vs. 4
**Baseline Differences**						
Latent vs. Control	0.9996	0.9996	0.9997	0.9996	0.9996	0.9996
Progressor vs. Control	0.9997	0.9997	0.9997	0.9997	0.9997	0.9997
Progressor vs. Latent	0.9997	0.9997	0.9997	0.9997	0.9997	0.9997
**Changes Over Time**						
Control	0.9996	0.9996	0.9996	0.9996	0.9996	0.9996
Latent	0.9996	0.9996	0.9996	0.9996	0.9996	0.9996
Progressor	0.9997	0.9997	0.9997	0.9997	0.9997	0.9997
**Differences in Changes Over Time**						
Latent vs. Control	0.9996	0.9996	0.9996	0.9996	0.9996	0.9996
Progressor vs. Control	0.9997	0.9997	0.9997	0.9997	0.9997	0.9997
Progressor vs. Latent	0.9997	0.9997	0.9997	0.9997	0.9997	0.9997

Table 11: Tuberculosis Dataset: Number of Significant Genes by Contrast in the 4 MCMSeq Runs. The number of differentially expressed genes (DEG) and equally expressed genes (EEG) common to all 4 runs and the percent of calls in agreement are also presented. Kappa-Fleiss agreement statistics were calculated across the 4 runs.

	Run 1	Run 2	Run 3	Run 4	Common DEGs	Common EEGs	Percent Calls in Agreement	Kappa-Fleiss Agreement
**Baseline Differences**								
Latent vs. Control	3	4	7	4	2	15792	99.96	0.59
Progressor vs. Control	650	667	679	650	583	15056	98.98	0.93
Progressor vs. Latent	256	258	284	272	207	15478	99.27	0.88
**Changes Over Time**								
Control	0	0	0	1	0	15881	> 99.9	0.00
Latent	5	3	3	1	1	15876	> 99.9	0.56
Progressor	1463	1511	1483	1513	1380	14214	98.70	0.96
**Differences in Changes Over Time**								
Latent vs. Control	3	3	2	3	0	15793	99.96	0.36
Progressor vs. Control	635	611	628	637	529	15078	98.78	0.91
Progressor vs. Latent	852	906	869	874	772	14817	98.66	0.93

References

[1] Box, G.E., Tiao, G.C.: Bayesian Inference in Statistical Analysis vol. 40. John Wiley & Sons, Hoboken (2011)

[2] Casella, G., Berger, R.L.: Reconciling bayesian and frequentist evidence in the one-sided testing problem. Journal of the American Statistical Association **82**(397), 106–111 (1987)

[3] Morris, J.S., Brown, P.J., Herrick, R.C., Baggerly, K.A., Coombes, K.R.: Bayesian analysis of mass spectrometry proteomic data using wavelet-based functional mixed models. Biometrics **64**(2), 479–489 (2008)

[4] Geweke, J., *et al.*: Evaluating the Accuracy of Sampling-based Approaches to the Calculation of Posterior Moments vol. 196. Federal Reserve Bank of Minneapolis, Research Department Minneapolis, MN, Minneapolis (1991)

[5] Singhania, A., Verma, R., Graham, C.M., Lee, J., Tran, T., Richardson, M., Lecine, P., Leissner, P., Berry, M.P., Wilkinson, R.J., *et al.*: A modular transcriptional signature identifies phenotypic heterogeneity of human tuberculosis infection. Nature Communications **9**(1), 2308 (2018)

[6] Rosenberg, B.R., Depla, M., Freije, C.A., Gaucher, D., Mazouz, S., Boisvert, M., Bédard, N., Bruneau, J., Rice, C.M., Shoukry, N.H.: Longitudinal transcriptomic characterization of the immune response to acute hepatitis c virus infection in patients with spontaneous viral clearance. PLoS pathogens **14**(9), 1007290 (2018)

[7] Fournier, D.A., Skaug, H.J., Ancheta, J., Ianelli, J., Magnusson, A., Maunder, M.N., Nielsen, A., Sibert, J.: AD Model Builder: using automatic differentiation for statistical inference of highly parameterized complex nonlinear models. Optim. Methods Softw. **27**, 233–249 (2012)

[8] Rudra, P., Shi, W.J., Vestal, B., Russell, P.H., Odell, A., Dowell, R.D., Radcliffe, R.A., Saba, L.M., Kechris, K.: Model based heritability scores for high-throughput sequencing data. BMC bioinformatics **18**(1), 143 (2017)

[9] Zhang, W.: cplm: Monte carlo em algorithms and bayesian methods for fitting tweedie compound poisson linear models. R package version 0.2-1 (2011)

[10] Kuznetsova, A., Brockhoff, P.B., Christensen, R.H.B.: lmerTest package: Tests in linear mixed effects models. Journal of Statistical Software **82**(13), 1–26 (2017). doi:10.18637/jss.v082.i13

[11] Satterthwaite, F.E.: An approximate distribution of estimates of variance components. Biometrics bulletin **2**(6), 110–114 (1946)

[12] Bian, Y., He, C., Hou, J., Cheng, J., Qiu, J.: Pairedfb: a full hierarchical bayesian model for paired rna-seq data with heterogeneous treatment effects. Bioinformatics (2018)

[13] Law, C.W., Chen, Y., Shi, W., Smyth, G.K.: voom: Precision weights unlock linear model analysis tools for rna-seq read counts. Genome biology **15**(2), 29 (2014)

[14] Smyth, G.K.: Limma: linear models for microarray data. In: Bioinformatics and Computational Biology Solutions Using R and Bioconductor, pp. 397–420. Springer, New York (2005)

[15] Sun, S., Hood, M., Scott, L., Peng, Q., Mukherjee, S., Tung, J., Zhou, X.: Differential expression analysis for rnaseq using poisson mixed models. Nucleic acids research **45**(11), 106–106 (2017)
